# Ultra-High Purity and Productivity Separation of CO_2_ and C_2_H_2_ from CH_4_ in Rigid Layered Ultramicroporous Material

**DOI:** 10.1021/acscentsci.4c01125

**Published:** 2024-09-20

**Authors:** Yuanyuan Jin, Tian Ke, Guihong Xu, Jinjian Li, Zhixin Jiang, Rongrong Fan, Zhiguo Zhang, Zongbi Bao, Qilong Ren, Qiwei Yang

**Affiliations:** † Key Laboratory of Biomass Chemical Engineering of Ministry of Education, College of Chemical and Biological Engineering, Zhejiang University, 310027 Hangzhou, Zhejiang, China; ‡ Institute of Zhejiang University-Quzhou, 324000 Quzhou, Zhejiang, China

## Abstract

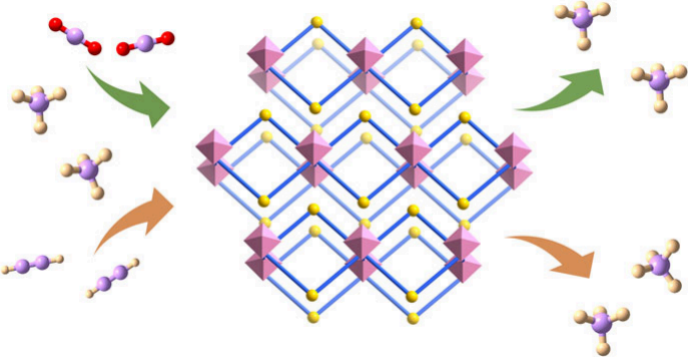

Efficiently obtaining both high-purity gas-phase and adsorbed-phase products in a single physisorption process presents the challenge of simultaneously achieving high selectivity and uptake and rapid diffusion in adsorbents. With a focus on natural gas purification and high-purity acetylene production, we report for the first time that the synergistic ligand/anion binding mode and multiple diffusion pathways in a robust 2D layered ultramicroporous framework (ZUL-100) enable unprecedented carbon dioxide/methane and acetylene/methane separation performance. Taking advantage of its rich anion, functional ligand ,and rigid 3D interpenetrated ultramicroporous channels, ZUL-100 achieved record IAST selectivities for equimolar carbon dioxide/methane (3.2 × 10^5^) and acetylene/methane (1.7 × 10^10^) mixtures, accompanied by record dynamic uptakes of carbon dioxide (3.10 mmol/g) and acetylene (4.79 mmol/g), respectively. The strong affinity and fast mass transfer of carbon dioxide and acetylene on ZUL-100 were systematically elucidated by a combination of in situ FTIR, single-crystal XRD, kinetic tests, and DFT-D adsorption/diffusion modeling. In particular, high-purity (≥99.999%) methane and carbon dioxide (acetylene) can both be obtained on ZUL-100 through a single adsorption–desorption cycle, with exceptional productivity (2.81–4.22 mmol/g of methane, 2.96 mmol/g of carbon dioxide, and 4.31 mmol/g of acetylene) and high yield (95.5% for carbon dioxide and 90.0% for acetylene).

## Abstract 2

The rigid metal−organic framework ZUL-100 with 3D interpenetrated ultramicroporous channels obtains both unprecedented carbon dioxide/methane and acetylene/methane separation performance through the synergy between ligands and anions, and both high-purity/productivity methane and carbon dioxide (acetylene) can be obtained via a single adsorption−desorption cycle.

## Introduction

As society increasingly prioritizes the reduction of air pollution and greenhouse gas emissions, natural gas (NG) is emerging as a preferred alternative to traditional fossil fuels, due to the fact that NG produces 25–30% less carbon dioxide (CO_2_) per joule of energy delivered than oil and 40–45% less than coal.^1−4^ Significantly, global consumption of NG exceeded 3.9 trillion cubic meters (Tcm) in 2023. The International Energy Agency (IEA) projects that this figure will rise to 4.19 Tcm by 2024. Methane (CH_4_) is the main component of NG, which is accompanied by gaseous impurities such as CO_2_, which can cause pipeline corrosion during NG transportation as well as affect the calorific value of CH_4_ and its chemical conversion process, so it must be efficiently separated from CH_4_ to meet the various industrial requirements.^5−7^ On the other hand, the separation of acetylene (C_2_H_2_) from CH_4_ is another important industrial process. As a basic chemical feedstock and combustion gas, C_2_H_2_ is widely used in metal cutting, welding, and the manufacture of synthetic rubbers and plastics.^8,9^ In particular, electronic-grade (≥99.99%) and ultra-high purity (≥99.999%) C_2_H_2_ is essential for the production of many high-end chemicals, and its value far exceeds that of industrial- and polymer-grade C_2_H_2_ (90–99.9%).^10−12^ However, since C_2_H_2_ derived from the CH_4_ oxidation process (a significant source of C_2_H_2_) coexists with unreacted CH_4_, efficient recovery of high-purity C_2_H_2_ from CH_4_ remains a real-world challenge.^13−15^

Solvent absorption is commonly used for CO_2_/CH_4_ and C_2_H_2_/CH_4_ separations.^16−18^ For example, organic amine solvents are commonly used to absorb CO_2_, but this chemical absorption process encounters an issue of the high energy consumption required for solvent regeneration, while the corrosive nature of amine solvents on equipment is also a serious consideration; acetone and *N*-methylpyrrolidone are commonly used as absorbents for C_2_H_2_, but the process still involves concerns of large solvent consumption and solvent volatilization.^19−21^ Therefore, the development of energy-saving and green separation methods has become a common requirement for the separation of CO_2_/CH_4_ and C_2_H_2_/CH_4_. Physisorption based on solid adsorbents is a promising candidate for CH_4_ purification and separation due to its advantages of low energy consumption and environmental compatibility. And the separation efficiency depends on the performance of the adsorbent. However, the kinetic diameters of CO_2_, C_2_H_2_, and CH_4_ molecules are 3.3, 3.3, and 3.8 Å, respectively, with intermolecular differences within the subangstrom scale (only 0.5 Å for both C_2_H_2_/CH_4_ and CO_2_/CH_4_), and the dipole moments of these molecules are all zero, posing a challenge for the development of efficient adsorbents.^22−26^

**Figure 1:**
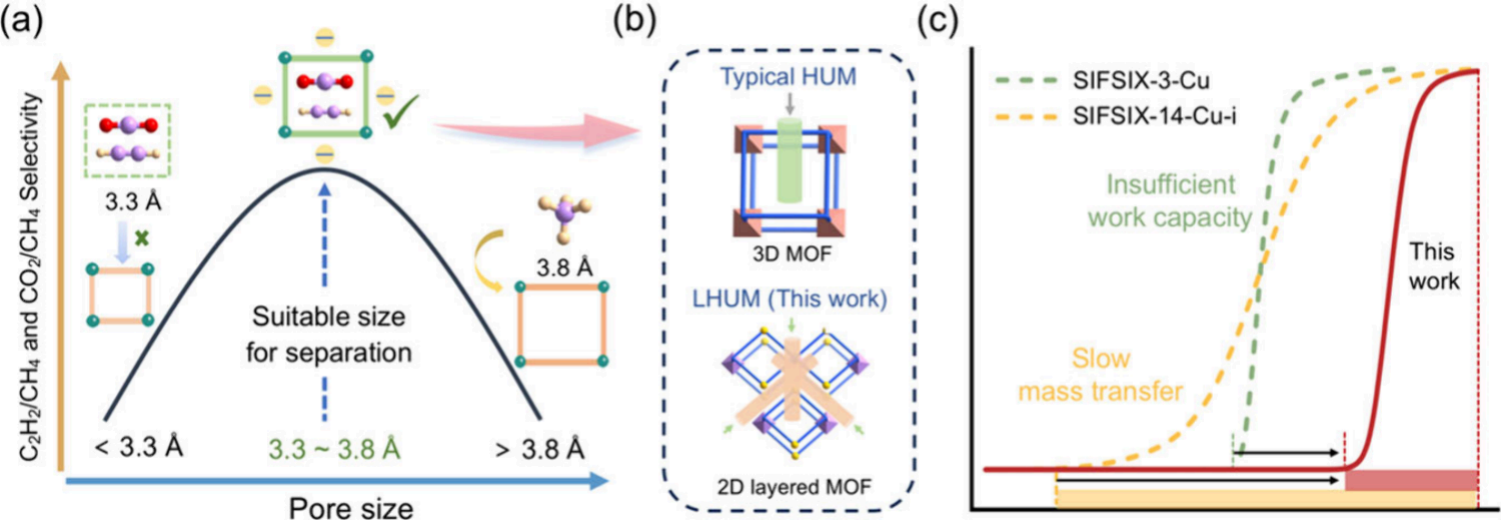
(a) Relationship between pore size and selectivity of CO_2_/CH_4_ and C_2_H_2_/CH_4_. (b) Topology types and pore channels in typical HUM and LHUM. (c) Three profiles of CO_2_ breakthrough curves in typical HUMs and LHUM.

Metal–organic frameworks are porous adsorbents that have emerged in the last two decades, with a rich variety of topologies with hierarchically structured motifs and higher structural tunability compared to conventional porous materials.^27−30^ At present, more than 40 kinds of MOFs have been investigated for the selective adsorption of CO_2_ and C_2_H_2_ from CH_4_.^31−34^ The pore sizes of the MOFs with high selectivity are in the range of 3.3–3.8 Å, which lie between the molecular sizes of CO_2_/C_2_H_2_ and CH_4_ (Figure 1a). Among these MOFs, the inorganic anion hybrid ultramicroporous MOFs (HUMs) represented by the SIFSIX platform have a strong affinity for CO_2_ and C_2_H_2_ thanks to their finely tunable ultramicropores and high-density electronegative binding sites.^35−38^ For example, pyrazine-based SIFSIX-3-Cu/Zn/Ni (with pore sizes of ∼3.5 Å) is one of the current benchmark adsorbents for CO_2_/CH_4_ separation due to its high CO_2_ affinity. Unfortunately, despite the high CO_2_ uptake at an ultralow pressure range, its adsorption capacity is not advantageous at higher pressures, and the high adsorption heat of CO_2_ hinders regeneration.^24,25,36^ Another typical HUM, 4,4′-azopyridine-based SIFSIX-14-Cu-i^39−41^ (UTSA-200, with a pore size of ∼3.4 Å), exhibits high selectivity for both CO_2_/CH_4_ and C_2_H_2_/CH_4_ separations. Nevertheless, its constrained 1D channel and structural flexibility restrict the kinetic diffusion, resulting in a wide mass transfer zone (corresponding to low adsorbent utilization efficiency) during the fixed-bed separations. Furthermore, SIFSIX-14-Cu-i suffers from poor water and air stability, limiting its applicability in real-world separation scenarios.^42,43^ Therefore, the development of an ideal adsorbent for the CH_4_ purification and separation, which should combine high uptake, excellent separation selectivity, rapid diffusion, and mild regeneration conditions, remains an unsolved and very challenging issue.

2D layered HUMs (LHUMs) are composed of stacked metal–organic layers by supramolecular interactions.^44,45^ These materials retain the properties of electronegative anions and ultramicropores of the conventional 3D HUMs, while exhibiting the unique layer-to-layer ordered supramolecular structure that enhances the structural stability and possessing 3D interconnected pore space within the intra/interlayer structure that provides multiple diffusion channels (Figure 1b). Taking advantage of these characteristics, LHUMs offer new opportunities to overcome the aforementioned bottlenecks. However, to the best of our knowledge, none of the current studies on layered 2D ionic hybrid materials focus on their application for the CH_4_ purification and separation. It remains an unknown challenge whether these materials can simultaneously achieve both high CO_2_/C_2_H_2_ uptake and effective CH_4_ exclusion, i.e., ultra-high selectivity.

Here, we report TIFSIX-dpso_2_-Cu (TIFSIX = hexafluorotitanate anion, TiF_6_^2–^; dpso_2_ = 4,4′-dipyridylsulfone) (also termed as ZUL-100) as a typical LHUM with excellent water/air stability and a rare rigid permanent pore structure among reported LHUMs, which addresses the multitarget challenge of separating CO_2_ and C_2_H_2_ from CH_4_ based on its unique mass transfer pore structure and surface chemistry. The intra- and interlayer three-dimensional interpenetrated pore channels in ZUL-100, along with the abundant functional sulfone groups and anions, are capable of providing multiple diffusion paths/suitable surface electrostatic environments for CO_2_ and C_2_H_2_, but with only a low affinity for CH_4_ which has a homogeneous near-neutral surface electrostatic potential (ESP). The notably higher adsorption enthalpies (derived from both experimental and simulated results) of CO_2_/C_2_H_2_ than CH_4_ proved the large affinity difference between them. A combination of guest-loaded single-crystal X-ray diffraction (SCXRD), in situ Fourier transform infrared (FTIR) spectroscopy, kinetic tests, and dispersion-corrected density functional theory (DFT-D) adsorption/diffusion calculations confirmed the synergistic binding mode and the rapid diffusion of CO_2_ (C_2_H_2_) in ZUL-100. Fixed-bed breakthrough tests validate the exceptional dynamic separation performance of ZUL-100: record high-purity (≥99.999%) CH_4_ production time and record CO_2_/C_2_H_2_ breakthrough uptakes for both CO_2_/CH_4_ and C_2_H_2_/CH_4_ mixtures, along with the ability to obtain high-purity CO_2_ and C_2_H_2_ under mild regeneration temperatures, which demonstrates the ultra-high selectivity of ZUL-100 on CO_2_/CH_4_ and C_2_H_2_/CH_4_ separation.

## Results and Discussion

**Figure 2:**
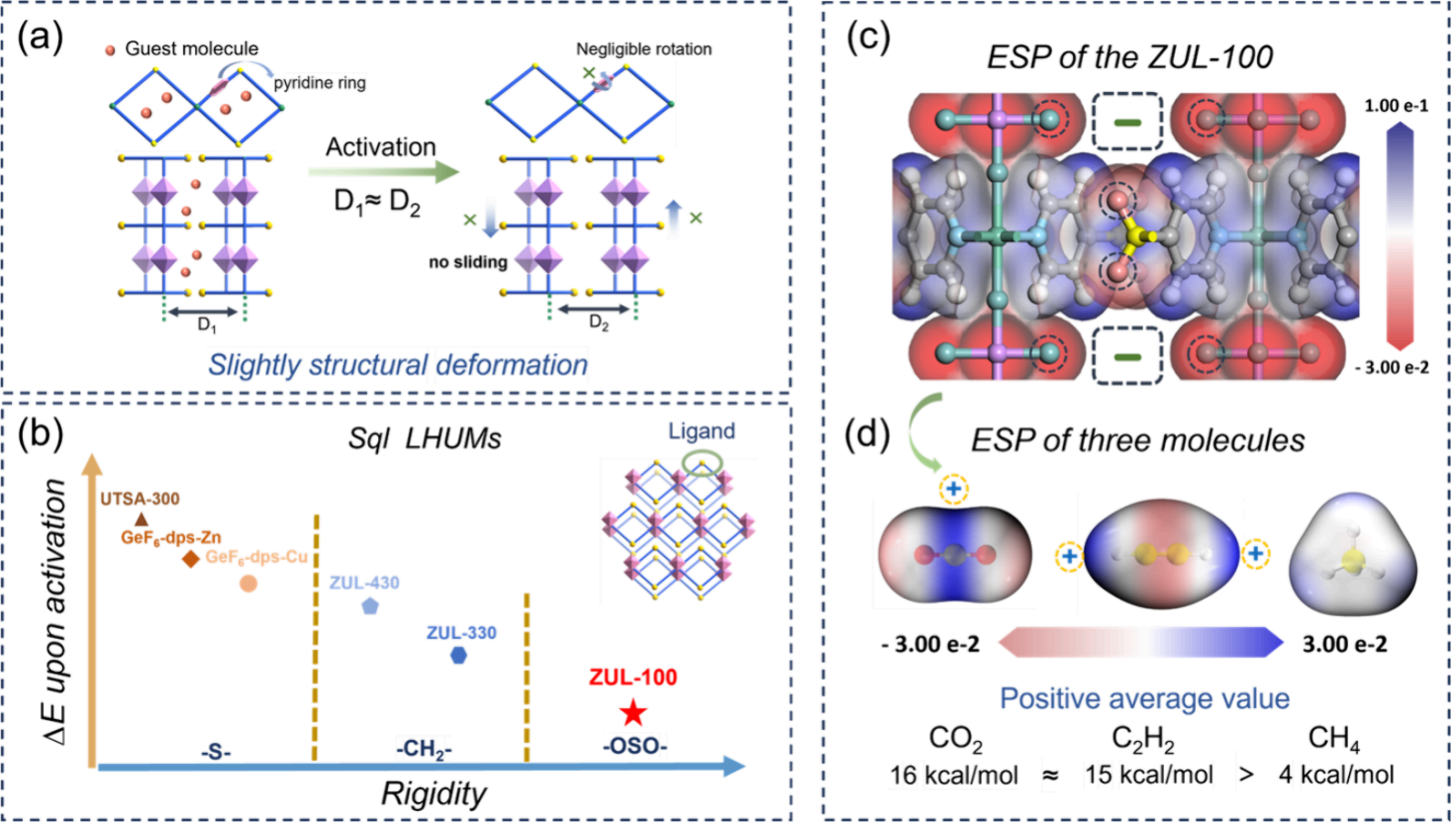
(a) Structure changes of ZUL-100 after activation. (b) Energy difference of frameworks (Δ*E*_framework_) upon activation of ZUL-100 and representative materials with the same sql topology. (c) Local surface electrostatic potential (ESP) of activated ZUL-100. (d) ESP of guest molecules. Color code: F, cyan; C, light gray in ZUL-100 and yellow in molecules; H, white; N, blue; Cu, green; O, pink in ZUL-100 and red in CO_2_; S, bright yellow; Ti, purple. Scale of the molecule mapped spans: −0.03 (red) through 0 (white) to 0.1 (blue) au of ZUL-100, −0.03 (red) through 0 (white) to 0.03 (blue) au of molecules.

ZUL-100 single-crystal samples can be synthesized by the interfacial slow diffusion method (the detailed synthesis procedure is provided in the Supporting Information). It is noted that our previous work reported the application of ZUL-100 in the purification of polymer-grade ethylene,^46^ but failed to obtain the crystal samples that retained their single-crystal morphology after activation (thus it was difficult to obtain the guest-loaded samples). In this work, we successfully obtained robust ZUL-100 single-crystal samples capable of maintaining single-crystal quality after activation and guest loading for SCXRD analysis, which is crucial for investigating the framework rigidity and the adsorption/diffusion mechanism. In as-synthesized and activated ZUL-100 crystals, single-layer networks with open pore windows are stacked through supramolecular interactions between adjacent layers, forming a porous network with 3D mass transfer channels. Interestingly, by analyzing the degree of pyridine ring rotation and the relative positions between layers before and after activation,we found that the framework undergoes negligible deformation upon activation (Figure 2a). In comparison, an examination of representative materials with the same sql topology (Figure 2b), including ZUL-330/430,^10^ GeFSIX-dps-Zn/Cu,^44^ and UTSA-300,^45^ revealed that the energy difference of frameworks (Δ*E*_framework_) upon activation of ZUL-100 is lower than that of those materials.

This implies that ZUL-100 has a higher structural rigidity, which is advantageous for reducing the diffusion resistance of guest molecules and extending the working pressure range. PXRD (Figure S1) results show that the positions and relative intensities of the characterized diffraction peaks remain consistent before and after activation, aligning with its single-crystal structure. Thermogravimetric (TGA) results show that the decomposition temperature of ZUL-100 exceeds 476 K (Figure S3), confirming its excellent thermal stability. 77 K N_2_ adsorption results reveal the permanent porosity of the activated ZUL-100 with a specific BET surface area of 492 m^2^/g. We quantitatively analyzed the surface electrostatic potentials (ESP) of ZUL-100 and CO_2_, C_2_H_2_, and CH_4_. As shown in Figure 2c,d, the ESP results indicate significantly larger positive potential areas for CO_2_ and C_2_H_2_ with their positive average value of 16 and 15 kcal/mol, respectively, much higher than the 4 kcal/mol of CH_4_. This suggests that compared to the relatively electrically neutral CH_4_, CO_2_ and C_2_H_2_ can achieve stronger affinity through electrostatic interactions with the structure, facilitating the achievement of high thermodynamic selectivity.

**Figure 3:**
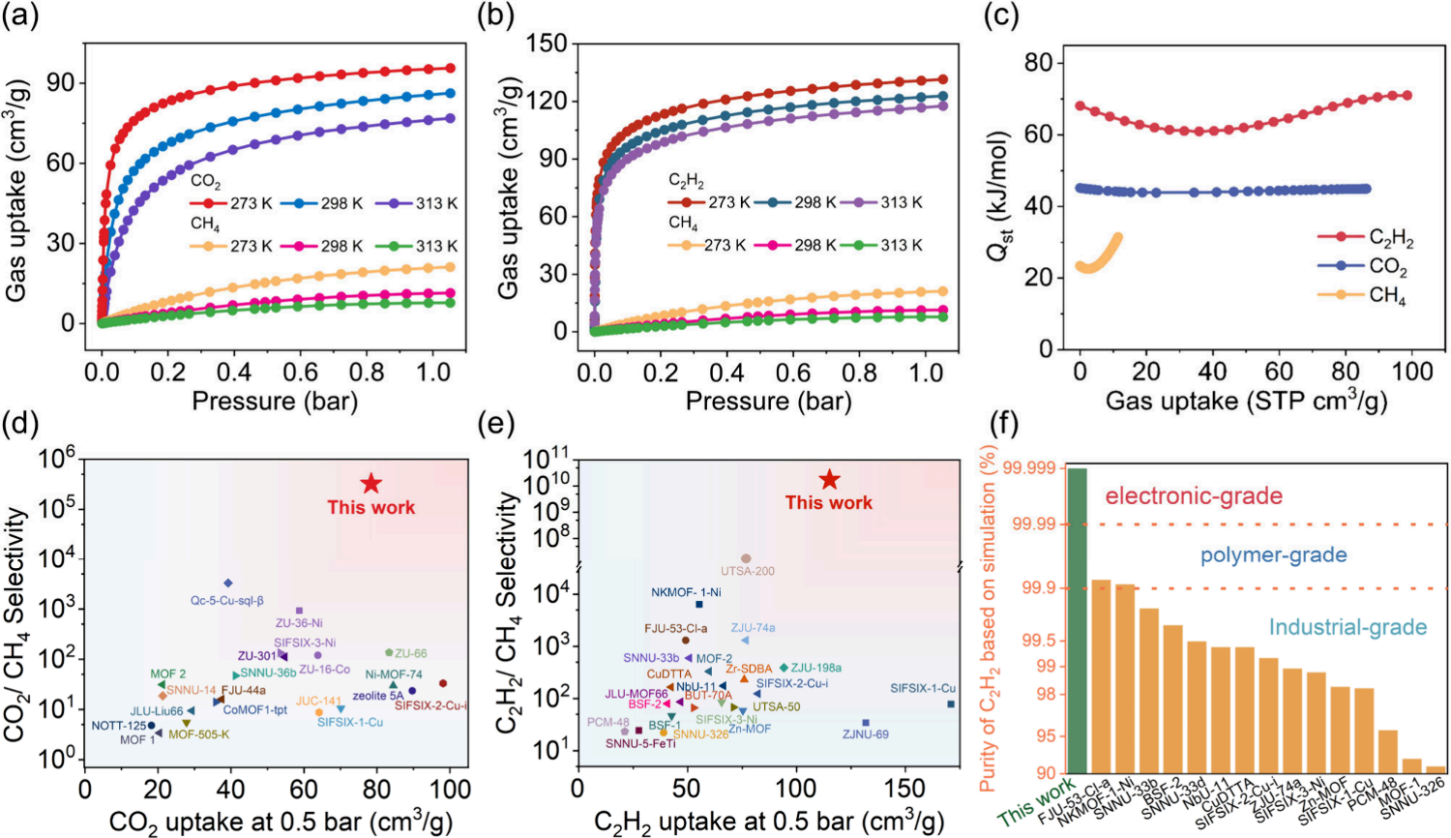
(a) CO_2_ and CH_4_ adsorption isotherms on ZUL-100 at 273, 298, and 313 K. (b) C_2_H_2_ and CH_4_ adsorption isotherms on ZUL-100 at 273, 298, and 313 K. (c) *Q*_st_ of C_2_H_2_, CO_2_, and CH_4_ of ZUL-100. (d,e) Comparison of the IAST selectivities and gas adsorption uptakes at 0.5 bar and 298 K of ZUL-100 with representative porous materials. (f) Comparison of the IAST simulated C_2_H_2_ purity in the adsorbed phase of ZUL-100 and representative porous materials for adsorption of an equimolar C_2_H_2_/CH_4_ mixture.

The adsorption isotherms of CO_2_, C_2_H_2_, and CH_4_ on ZUL-100 were determined at 273, 298, and 313 K. As shown in Figure 3a,b, ZUL-100 exhibits a type I isotherm shape for CO_2_ and C_2_H_2_ with high adsorption uptakes. For example, the CO_2_ adsorption uptake reached 78.4 cm^3^/g at 0.5 bar and 298 K, exceeding those of typical HUMs SIFSIX-3-Cu (56.5 cm^3^/g),^24^ SIFSIX-14-Cu-i (76.6 cm^3^/g),^41^ Qc-5-Cu-sql-β (39.2 cm^3^/g),^31^ and SIFSIX-1-Cu (70.1 cm^3^/g)^23^ used for CO_2_ adsorption and separation, respectively. Additionally, the C_2_H_2_ uptake of ZUL-100 under the same conditions was 115.1 cm^3^/g (this value is 3.60% higher than the literature isotherm data,^46^ 111.1 cm^3^/g). In contrast, the CH_4_ adsorption isotherm exhibited a relatively flat linear shape, with an adsorption uptake of only 8.1 cm^3^/g at 0.5 bar. The significant uptake/profile differences of gas adsorption isotherms suggest that ZUL-100 may have much higher CO_2_/C_2_H_2_ affinities than CH_4_. The adsorption heats (*Q*_st_) of ZUL-100 for the three gases were further calculated by fitting isotherms at different temperatures by using the virial equation. As shown in Figure 3c, the *Q*_st_ values of ZUL-100 for CO_2_ and C_2_H_2_ are 45.2 and 68.1 kJ/mol, respectively, significantly higher than that of CH_4_ (23.4 kJ/mol), which confirms the potential of ZUL-100 in separating CO_2_/CH_4_ and C_2_H_2_/CH_4_. The IAST selectivities of ZUL-100 for equimolar CO_2_/CH_4_ and C_2_H_2_/CH_4_ mixtures were calculated by fitting the experimental isotherms using the dual-site Langmuir–Freundlich (DSLF, for CO_2_ and C_2_H_2_) and single-site Langmuir–Freundlich (SSLF, for CH_4_) equations, respectively. Notably, at 298 K and 1 bar, ZUL-100 achieves an unprecedented CO_2_/CH_4_ selectivity of 3.2 × 10^5^ (Figure 3d), which is significantly higher than those of all previously reported adsorbents. Similarly, the selectivity of ZUL-100 for C_2_H_2_/CH_4_ also reaches a record value of 1.7 × 10^10^ (Figure 3e). At the same time, the IAST selectivities for the CO_2_/CH_4_ and C_2_H_2_/CH_4_ mixtures at different ratios (relevant to different industrial scenarios) remained excellent on ZUL-100 (Figures S13 and S14). Furthermore, ZUL-100 exhibits both high uptake and selectivity among all porous materials, including HUMs and other MOFs for CO_2_/CH_4_ and C_2_H_2_/CH_4_ separations, confirming its potential to break the trade-off between adsorption capacity and selectivity.

**Figure 4:**
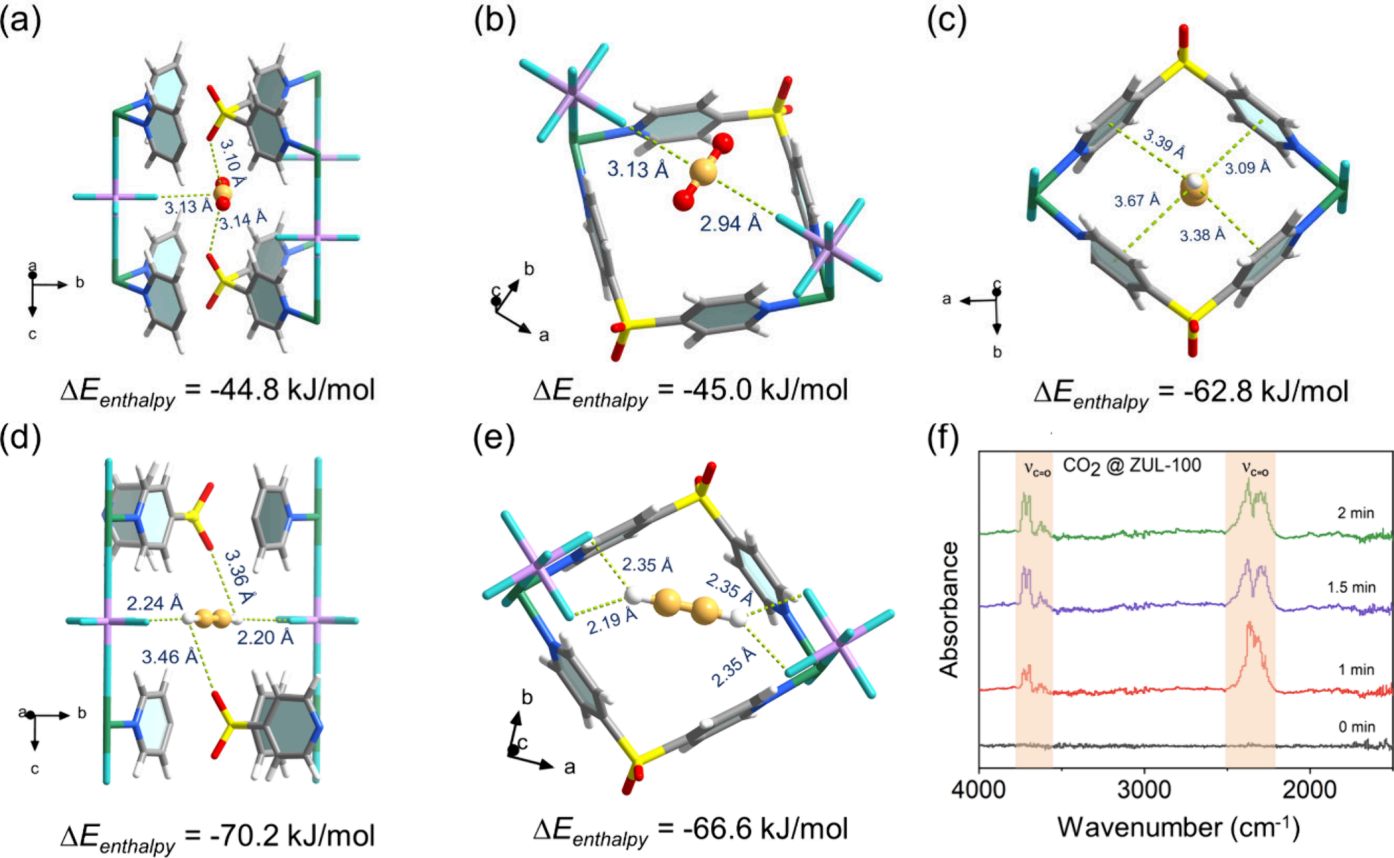
(a–e) Different binding sites in the single-crystal structures of CO_2_/C_2_H_2_-loaded ZUL-100: (a) CO_2_ binding site I, (b) CO_2_ binding site II, (c) C_2_H_2_ binding site I, (d) C_2_H_2_ binding site II, and (e) C_2_H_2_ binding site III. (f) In situ FTIR of activated ZUL-100 under a CO_2_ atmosphere. Color code: F, cyan; C, light gray in ZUL-100 and orange in CO_2_/C_2_H_2_; H, white; N, blue; Cu, green; O, red; S, bright yellow; Ti, purple.

The IAST uptakes of CO_2_, C_2_H_2_, and CH_4_ on ZUL-100 for the adsorption of equimolar CO_2_/CH_4_ or C_2_H_2_/CH_4_ mixtures at 298 K were calculated, respectively. The result implied the strong competitive adsorption effects of CO_2_ and C_2_H_2_ over CH_4_, that the CH_4_ uptake for the CO_2_/CH_4_ mixture is only 1.1 × 10^–5^ mmol/g and even less than 1 × 10^–6^ mmol/g for the C_2_H_2_/CH_4_ mixture (Figures S9 and S10), indicating that ZUL-100 is expected to achieve dynamic exclusion of CH_4_ in actual separation processes. In particular, we evaluated the C_2_H_2_ purity in the adsorbed phase of different adsorbents (Figure 3f). The results indicated that the C_2_H_2_ purity of ZUL-100 reached over 99.999%, highlighting its potential for recovering high-purity C_2_H_2_ from CH_4_. In addition, we tested the CO_2_ adsorption isotherms on ZUL-100 after immersion in water or exposure to air for 7 days (Figure S8), and the results showed that the isotherm profile and the CO_2_ uptake were hardly affected, indicating the excellent water–air stability of ZUL-100. In order to further elucidate the adsorption mechanism behind the strong affinity of CO_2_ and C_2_H_2_, we conducted SCXRD analysis of the CO_2_-loaded and C_2_H_2_-loaded ZUL-100 single-crystals. The results indicate that there are two possible binding sites for CO_2_ in ZUL-100. As shown in Figure 4a,b, site I involves strong interactions between the C atom from the CO_2_ molecule with positive ESP and the O atom from the sulfonyl group and the F atom from the TIF_6_^2–^ anion with negative ESP, which strongly reflects the synergy of the ligand and anion with CO_2_. The C···O and C···F distances were measured as 3.14 and 3.13 Å, respectively. Site II for CO_2_ binding is located in the interlayer channel, involving strong interactions between the C atom from the CO_2_ molecule and the F atom from the TIF_6_^2–^ anion, with C···F distances of 3.13 and 2.94 Å. Such a multiple binding mode of CO_2_ in different channels within ZUL-100 results in a strong CO_2_ affinity, as evidenced by the high simulated adsorption enthalpies (Δ*E*_enthalpy_) of 44.8 (site I) and 45.0 (site II) kJ/mol calculated from DFT-D simulations. For a C_2_H_2_-loaded ZUL-100 single crystal (Figure 4c–e), it was noted that C_2_H_2_ in ZUL-100 can be adsorbed at three possible sites: site I and site II for C_2_H_2_ binding are similar to the results of CO_2_-loaded ZUL-100, where the two H atoms of C_2_H_2_ were both captured by two TIF_6_^2–^ anions through strong hydrogen bonds (with C–H···F distances of 2.19–2.35 Å); site III C_2_H_2_ binding was located within the intralayer space surrounded by two dpso_2_ ligands, and multiple van der Waals interactions were observed with the distances between C_2_H_2_ molecule and pyridine rings ranging from 3.09 to 3.39 Å. The Δ*E*_enthalpy_ values of C_2_H_2_ at the three sites also reached 70.2, 66.6, and 62.8 kJ/mol, respectively. However, the Δ*E*_enthalpy_ value of CH_4_ is only 25.1 kJ/mol, which is notably lower than those of CO_2_ and C_2_H_2_ (Figure S27). Overall, the simulated Δ*E*_enthalpy_ values of CO_2_, C_2_H_,2_ and CH_4_ are consistent with the experimental results of *Q*_st_, confirming the significant binding affinity difference of these gases on ZUL-100. In situ FTIR spectroscopy was conducted for the adsorption of CO_2_ on ZUL-100. As shown in Figure 4f, it is observed that the characteristic peak of CO_2_ in the range of 2270–2370 cm^–1^ is splitting into two peaks during adsorption compared with pure that of CO_2_ (2345 cm^–1^), suggesting that there exist two different CO_2_–framework interactions which correspond to the SCXRD and DFT-D simulation results. Furthermore, we observed that IR vibration peaks reach equilibrium within 2 min, implying the rapid diffusion of CO_2_ in ZUL-100.

**Figure 5:**
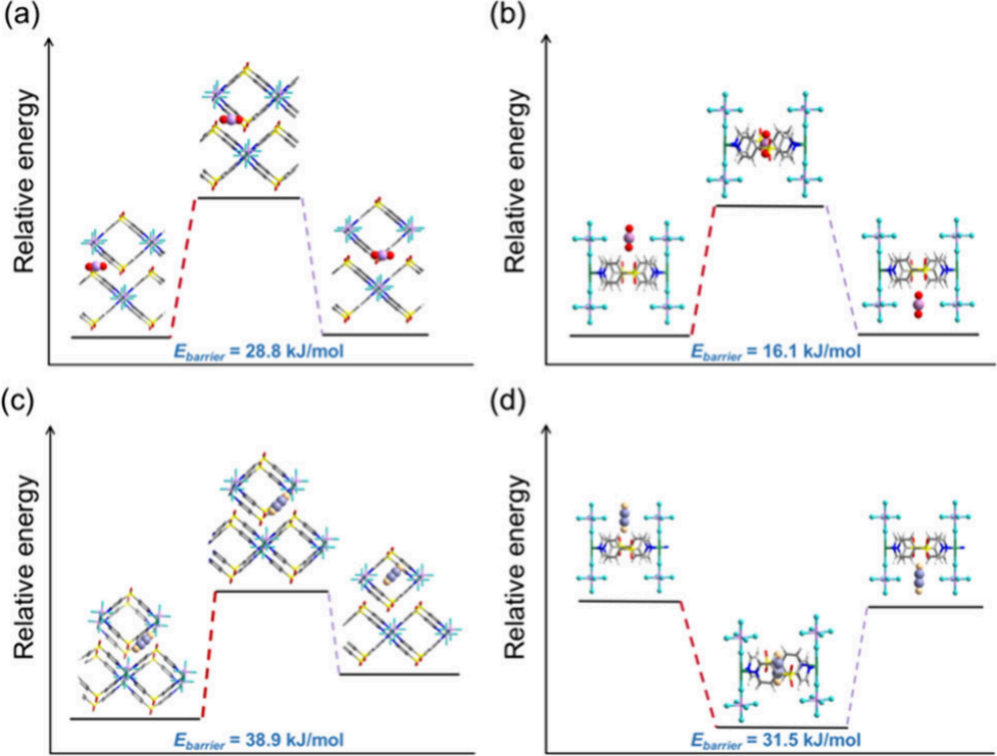
Diffusion barriers of CO_2_ and C_2_H_2_ by DFT-D calculation based on the activated ZUL-100 crystal structure. (a) CO_2_ diffusion through the interlayer channel. (b) CO_2_ diffusion through the intralayer channel. (c) C_2_H_2_ diffusion through the interlayer channel. (d) C_2_H_2_ diffusion through the intralayer channel.

Kinetic adsorption tests of CO_2_ and C_2_H_2_ on ZUL-100 at 298 K were performed. The results (Figures S15 and S16) showed that ZUL-100 reached adsorption equilibrium in both gases within 2 min at 0.5 bar, demonstrating its excellent mass transfer rate. In addition, in situ PXRD patterns of ZUL-100 loaded with CO_2_ and CH_4_ showed that the PXRD patterns were almost the same under different gas loadings, while a similar phenomenon was observed in the PXRD pattern of ZUL-100 after loading of C_2_H_2_ at 0.25, 0.5, 0.75, and 1 bar, indicating the structural rigidity of ZUL-100. Diffusion energy barriers (*E*_barrier_) for CO_2_ and C_2_H_2_ within the channels of the activated ZUL-100 framework were calculated to elucidate the mass transfer behavior of these gases. As shown in Figure 5 and Figures S29–S32, guest molecules were moved incrementally (with a step size of 0.85/1 Å) along both the intralayer (along the *c*-axis direction) and the interlayer (within the ab plane) directions. The energy calculation results showed that the *E*_barrier_ values for CO_2_ were only 28.8 (Figure 5a) and 16.1 kJ/mol (Figure 5b) for passing through the interlayer and intralayer channels, respectively, while the *E*_barrier_ values of C_2_H_2_ were also only 38.9 (Figure 5c) and 31.5 kJ/mol (Figure 5d). Furthermore, a comparative analysis of the *E*_barrier_ values on the robust ZUL-100 framework with another isostructural but flexible framework (ZUL-330,^10^ which undergoes structural deformation upon activation) was performed to evaluate the influence of the structural rigidity and flexibility on gas diffusion in LHUMs. The results show that while *E*_barrier_ for C_2_H_2_ in the gate-opened ZUL-330 (27.0 kJ/mol) was comparable to that in ZUL-100, this value increased by 89.2% (51.1 kJ/mol) in the activated ZUL-330 (before gate-opening). This result further supports the premise that the rigid structure of ZUL-100 with multiple mass transfer channels facilitates rapid diffusion even at ultralow concentrations.

**Figure 6:**
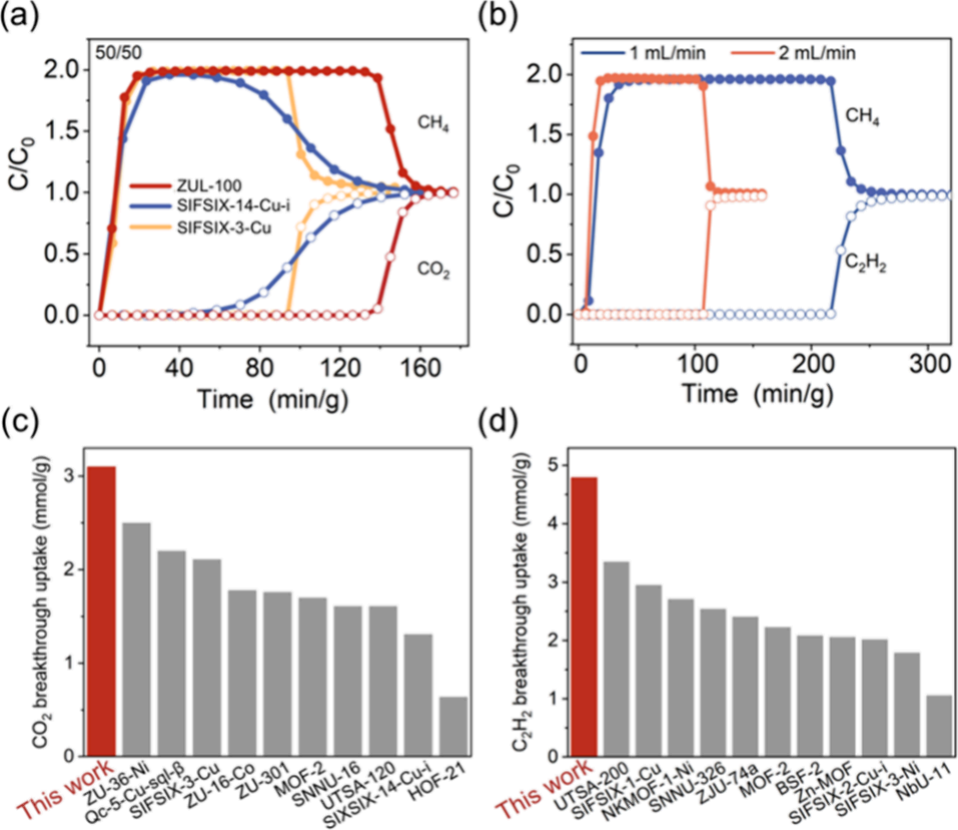
(a) Breakthrough curves of equimolar CO_2_/CH_4_ mixture on ZUL-100 (red), SIFSIX-3-Cu (yellow), and SIFSIX-14-Cu-i (blue) at 298 K. (b) Breakthrough curves of equimolar C_2_H_2_/CH_4_ mixture on ZUL-100 under different flow rates. (c,d) Comparison of the breakthrough uptake of ZUL-100 with those of representative MOFs for equimolar CO_2_/CH_4_ and C_2_H_2_/CH_4_ mixtures at 298 K.

Dynamic breakthrough experiments were conducted on ZUL-100 for the CO_2_/CH_4_ (50/50, *v*/*v*) and C_2_H_2_/CH_4_ (50/50, *v*/*v*) mixtures, respectively. For the CO_2_/CH_4_ (50/50, *v*/*v*) mixture, at 298 K with a flow rate of 1 mL/min, the CH_4_ broke through the ZUL-100 column and reached its equilibrium concentration rapidly (Figure 6a), indicating a dynamic exclusion of CH_4_ which was consistent with the calculated IAST uptake. However, CO_2_ was retained in the ZUL-100 column for a much longer time of 138.7 min/g, corresponding to an unprecedented breakthrough uptake of 3.10 mmol/g (calculation based on the first point for CO_2_ being detected), which surpassed all previously reported adsorbents (Figure 6c). Additionally, CH_4_ with a purity exceeding 99.999% can be recovered at the column outlet with a productivity of 2.81 mmol/g. Moreover, it is worth noting that the mass transfer zone of CO_2_ is narrow, consistent with the diffusion energy barrier analysis, confirming its rapid diffusion. At the same time, the CO_2_/CH_4_ separation performance was not affected under 1000 ppm of H_2_O (Figure S37). For comparison, the breakthrough curves of the gas mixture on SIFSIX-3-Cu and SIFSIX-14-Cu-i (Figure 6a) were also evaluated under identical conditions. The results showed that the retention times of CO_2_ on both of these materials were notably shorter than that on ZUL-100, 94.6 and 58.5 min/g, corresponding to CO_2_ breakthrough uptakes of 2.11 and 1.31 mmol/g, respectively. Moreover, a much wider mass transfer zone was observed in SIFSIX-14-Cu-i, implying its slow diffusion. The dynamic CO_2_/CH_4_ separation performance of these three materials was also evaluated with a 15/85 CO_2_/CH_4_ ratio, and the results showed that ZUL-100 still exhibited much higher CO_2_ uptake (2.68 mmol/g) and CH_4_ productivity (14.6 mmol/g) than SIFSIX-14-Cu-i (0.64 and 3.64 mmol/g) and SIFSIX-3-Cu (2.02 and 10.1 mmol/g) despite the lower CO_2_ concentration. Similarly, ZUL-100 also exhibited excellent separation performance for the C_2_H_2_/CH_4_ (50/50, *v*/*v*) mixture, where the dynamic breakthrough uptake of C_2_H_2_ and the 99.999% CH_4_ productivity reached 4.79 and 4.22 mmol/g at 298 K (Figure 6b).

**Figure 7:**
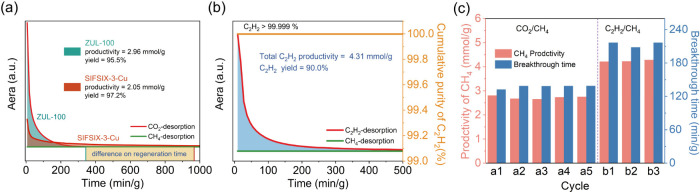
(a) Desorption curves for saturated ZUL-100 and SIFSIX-3-Cu columns of equimolar CO_2_/CH_4_ mixture with He purge at 313 K. (b) Desorption curves for saturated ZUL-100 of an equimolar C_2_H_2_/CH_4_ mixture with He purge at 353 K. (c) Breakthrough time and high-purity CH_4_ productivity for five consecutive cycles for the equimolar CO_2_/CH_4_ mixture and three cycles for the equimolar C_2_H_2_/CH_4_ mixture at 298 K.

The regeneration performance of ZUL-100 was evaluated by fixed-bed desorption experiments on a gas-saturated column. As shown in Figure 7a, at 313 K with a helium purge flow rate of 10 mL/min, ZUL-100 can be almost completely regenerated within 346.9 min/g, achieving a recovery of 99.999% CO_2_ at the column outlet with a desorption amount of 2.96 mmol/g, which corresponds to a high yield of 97.8% relative to the equilibrium dynamic uptake. This indicates that the ZUL-100 can be easily regenerated under mild conditions. For comparison, a desorption experiment was also performed on SIFSIX-3-Cu under the same condition, which showed that SIFSIX-3-Cu exhibited a significantly longer regeneration time (>967.2 min/g) compared to ZUL-100, due to its excessively strong CO_2_ binding affinity. In addition, as shown in Figure 7b, ZUL-100 achieved an exceptional 99.999% C_2_H_2_ productivity (4.31 mmol/g) from regeneration, corresponding to a high yield of 90.0% relative to the equilibrium dynamic uptake, at 353 K with a helium purge flow rate of 10 mL/min. These fixed-bed adsorption/desorption results indicate that ZUL-100 can simultaneously produce 99.999% CH_4_ and CO_2_/C_2_H_2_ products through an adsorption–desorption coupling process. Moreover, the results from eight cycles of breakthrough experiments (including three cycles of C_2_H_2_/CH_4_ and five cycles of CO_2_/CH_4_ separations) showed consistent curve shapes and the corresponding gas breakthrough times of ZUL-100 remained consistent, confirming its excellent cyclic regeneration capability (Figure 7c).

## Conclusion

Adsorption technologies using porous adsorbents hold promise for replacing traditional solvent absorption, which entail significant material and energy consumption issues for the CO_2_/CH_4_ and C_2_H_2_/CH_4_ separations. The challenge lies in overcoming the trade-off between adsorption capacity and selectivity, while simultaneously achieving rapid diffusion and excellent stability. In response to this challenge, a comprehensive study on the adsorptive separation of CO_2_/CH_4_ and C_2_H_2_/CH_4_ mixtures was conducted, showcasing the effectiveness of a robust LHUM, i.e., ZUL-100 in this work, as a highly efficient adsorbent. Characterized by its 3D inter- and intralayer porous channels, along with the synergistic interaction of ligand and anion, ZUL-100 demonstrated high-capacity and rapid-diffusion molecular sieving performance for CO_2_/CH_4_ and C_2_H_2_/CH_4_ mixtures, exhibiting superiority over conventional HUMs. The separation efficacy of ZUL-100 was further demonstrated by guest-loaded SCXRD, DFT-D calculations, and in situ FTIR analysis, which revealed its inimitable structure synergy and 3D guest diffusion mode. Overall, this study not only establishes a new benchmark for CH_4_ purification and separation via adsorption technology but also paves the way for the innovative design of efficient porous materials.

## Supporting Information Available

Experimental procedures, characterization data, and structural simulation information (PDF)
CO_2_-loaded ZUL-100 (CIF)
Activated ZUL-100 (CIF)
Activated ZUL-100(PDF)
C_2_H_2_-loaded ZUL-100 (PDF)
CO_2_-loaded ZUL-100 (PDF)
C_2_H_2_-loaded ZUL-100 (CIF)
Transparent Peer Review report available (PDF)

## Supplementary Material

oc4c01125_si_001.pdf

oc4c01125_si_002.cif

oc4c01125_si_003.cif

oc4c01125_si_006.pdf

oc4c01125_si_007.cif
