# Supplementary material for: Ultra-High Purity and Productivity Separation of CO2 and C2H2 from CH4 in Rigid Layered Ultramicroporous Material
Source: ACS Cent Sci. 2024 Sep 20;10(10):1885–93. doi: 10.1021/acscentsci.4c01125 (PMC11503503; doi:10.1021/acscentsci.4c01125)
Supplement: Supplementary file 1 — oc4c01125_si_001.pdf [file oc4c01125_si_001.pdf]

Supporting Information

Ultra-High Purity and Productivity Separation of CO_2 and C_2H_2 from CH_4 in Rigid Layered Ultramicroporous Material

Yuanyuan Jin[1], Tian Ke[1]*, Guihong Xu[1], Jinjian Li[1], Zhixin Jiang[1], Rongrong Fan[1], Zhiguo Zhang[1,2], Zongbi Bao[1,2], Qilong Ren[1,2], and Qiwei Yang[1,2*]

[1]Key Laboratory of Biomass Chemical Engineering of Ministry of Education, College of Chemical and Biological Engineering, Zhejiang University, 310027 Hangzhou, Zhejiang, China.

[2]Institute of Zhejiang University-Quzhou, 324000 Quzhou, Zhejiang, China.

*Correspondence: yangqw@zju.edu.cn (Q.Y.), ketian@zju.edu.cn (T.K.)

Experimental Section

Synthesis of materials

Preparation of ZUL-100 single crystal:
As-syntheszied ZUL-100 single crystal: A dimethyl sulfoxide (DMSO) solution (5 mL) of $Cu(BF_4)_2$•xH_2O and $(NH_4)_2TiF_6$ was prepared and added into a test tube with the size of Φ 2 cm × 10 cm. Then 1 mL methanol was dropped above the solution carefully as buffer layer. After that, a methanol solution (5 mL) of 4,4'-dipyridylsulfone ($dpso_2$) ligand (0.5 mmol) was prepared and added above the buffer layer slowly, then sealed the test tube for standing reaction, and after a month the purple crystals were harvested.
Activated ZUL-100 single crystal: The as-synthesized ZUL-100 single crystals were washed with methanol three times, then the crystal samples were degassed at 373 K (below 10 μmHg) for 6 h to obtain the activated ZUL-100 single crystals.

Preparation of ZUL-100 powder sample:
Scale up for powder sample of ZUL-100 was synthesized by a simple mixed stirring of aqueous solution (10 mL) of $Cu(BF_4)_2$•xH_2O (0.5 mmol) and $(NH_4)_2TiF_6$ with methanol solution (10 mL) of $dpso_2$ ligand (1 mmol) at 268 K for 24 h.

Preparation of SIFSIX-3-Cu powder sample:
The powder sample of SIFSIX-3-Cu was synthesized as the reported reference[1], through layering a methanol solution of pyrazine in a glass tube onto a methanol solution of $CuSiF_6 \cdot H_2O$. On layering, an extremely fast formation of light violet powder was observed, which was left for 24 h in the mother solution.

Preparation of SIFSIX-14-Cu-i powder sample:
The powder sample of SIFSIX-14-Cu-i was synthesized as the reported reference[2], through dropping an ethylene glycol solution (4.0 mL) of $Cu(BF_4)_2$•xH_2O (0.26 mmol) and $(NH_4)_2SiF_6$ (0.26 mmol) into a preheated ethanol solution (4.0 mL) of 4,4'-azopyridine (0.286 mmol), then the mixture were stirred and heated at 75 °C for 12 h. The obtained sample was exchanged with methanol for a week.

Single-crystal X-ray diffraction

Single crystal X-ray diffraction data for the activated and the CO_2/C_2H_2-loaded samples of ZUL-100 were collected on a Bruker-AXS D8 VENTURE diffractometer equipped with a PHOTON II detector. Indexing was performed using APEX3. Data integration and reduction were completed using SaintPlus 6.01. Absorption correction was performed by the multi-scan method implemented in SADABS. The space group was determined using XPREP implemented in APEX 3. The structure was solved with SHELXS-97 contained in APEX 3, WinGX v1.70.01, and refined on F2 (nonlinear least-squares method), and OLEX2 v1.1.5 program packages. All non-hydrogen atoms were refined anisotropically. The contribution of disordered solvent molecules was treated as diffuse using the Squeeze routine implemented in Platon. The crystal data are summarized in Table S2-S3.

Powder X-ray diffraction (PXRD)

Powder X-ray diffraction and C_2H_2-loaded data of the samples were collected on a SHIMADZU XRD-6000 diffractometer (Cu Kαλ = 1.540598 Å) with an operating power of 40 KV, 30 mA and a scan speed of 4.0°/min. The range of 2θ was from 5° to 60°. CO_2 and CH_4 atmosphere in-situ X-ray powder diffraction experiments were conducted on the same instrument using corresponding gases at a rate of 2 mL/min and a scan speed of 30°/min.

Thermogravimetric Analysis (TGA)

The thermal gravimetric analysis was performed on SDT650. Experiments were going on an alumina pan under nitrogen flow with a heating rate of 10 K/min from 303 K to 800 K.

Gas adsorption measurements

Before adsorption isotherms test, the synthesized samples were exchanged with methanol 3 times daily for 3 days. After that, ZUL- 100 was evacuated at 373 K for 24 h until the pressure below 10 μmHg. Degassing and gas adsorption measurements were conducted on a Micromeritics ASAP 2460 surface area analyzer. The adsorption isotherms were collected at 273 ~ 313 K on activated samples.

Adsorption kinetics tests

The adsorption kinetics curves at 298 K for ZUL-100 were measured by the Vacuum Vapor/Gas Sorption Analyzer (BDS-VVS, Beishide Instrument Technology (Beijing) Co., Ltd). The material was evacuated by vacuum heating at 100 °C overnight before measurement. The assessment criteria for the adsorption equilibrium was set at 0.1 mg/60 min.

Fitting of pure component isotherms

The adsorption isotherms of CO_2 and C_2H_2 in employed MOFs were fitted using a dual-site Langmuir-Freundlich model.

$$q = q_{A,\ sat}\frac{b_A p^{v_A}}{1+b_A v_A} + q_{B,\ sat}\frac{b_B p^{v_B}}{1+b_B v_B} \qquad (1)$$

The adsorption isotherms of CH_4 in employed MOFs were fitted using a single-site Langmuir-Freundlich model.

$$q = q_{sat}\frac{bp^{v}}{1+bv} \qquad (2)$$

Here, p is the pressure of the bulk gas at equilibrium with the adsorbed phase (kPa), q is the adsorbed amount per mass of adsorbent (mmol/g), $q_{A,sat}$ and $q_{B,sat}$ are the saturation capacities of site A and B (mmol/g), b_A and b_B are the affinity coefficients of site 1 and 2 (kPa^{-1}), and ν_A and ν_B represent the deviations from an ideal homogeneous surface. The parameters that were obtained from the fitting isotherms are provided in Table S4-S5, respectively.

Isosteric heat of adsorption

The experimental isosteric heat of adsorption (Q_{st}) values for CO_2, C_2H_2 and CH_4 in ZUL-100 were calculated using Virial type expression:

$$\ln P = \ln P + 1/T\sum_{i=0}^{m} a_i N^i + \sum_{i=0}^{m}\binom{n}{k} b_i N^i \quad (3)$$

Then the experimental isosteric heat of adsorption (Q_{st}) values for CO_2, C_2H_2 and CH_4 in ZUL-100 were calculated using equation[3]:

$$Q_{st} = -R\sum_{i=0}^{m} a_i N^i \qquad (4)$$

Where P is the pressure described in kPa, N is the adsorption capacity in mmol g^{-1}, T is the temperature in K, a_i and bi are Virial coefficients, and m and n are the numbers of coefficients used to describe the isotherms. Q_{st} is the coverage dependent enthalpy of adsorption, and R is the universal gas constant.

The related parameters were derived from the fitting of experimental gas adsorption isotherms at different temperatures (as shown in Fig. S14-16). The fitting was carried out by using the Nonlinear Curve Fit with the Multi-Data Fit Mode of global fit in the OriginPro software, Fitting code. The parameters are provided in Table S6-S8, respectively.

IAST calculation of adsorption selectivities

The Ideal Adsorbed Solution Theory (IAST) selectivity for CO_2/CH_4 and C_2H_2/CH_4 separation is defined by[4]

$$S_{ads} = \frac{q_1/q_2}{p_1/p_2} \qquad (5)$$

q_1 and q_2 are the molar loadings in the adsorbed phase in equilibrium with the bulk gas phase with partial pressures p_1 and p_2, which are calculated by fitting the experimental gas adsorption isotherms

using the dual-site Langmuir-Freundlich model (as shown in the Fitting of pure component isotherms), based on the IAST of Myers and Prausnitz using the equations as follows:

$$x_1 p_1(\pi) = P y_1 \quad (6)$$

$$x_2 p_2(\pi) = P y_2 \quad (7)$$

$$\frac{1}{q} = \frac{x_1}{q_1} + \frac{x_2}{q_2} \quad (8)$$

where x_1 and x_2 are the molar fractions of component 1 and 2 in the adsorbed phase, y_1 and y_2 are the molar fractions of component 1 and 2 in the bulk gas phase, at spreading pressure π and temperature T. Here, the parameters of q_1 and q_2 in the equations are corresponding to the IAST predicted CO_2, C_2H_2 and CH_4 uptakes of 50/50 (*v/v*) CO_2/CH_4 and C_2H_2/CH_4 co-adsorption. (Fig. S17)

Density-functional theory calculations

The static binding energy was calculated using the combination of first-principle density functional theory (DFT) and plane-wave ultrasoft pseudopotential implemented in the Materials Studio, CASTEP code[5]. A semi-empirical addition of dispersive forces to conventional DFT was included in the calculation to account for van der Waals interactions[6]. Calculations were performed under the generalized gradient approximation (GGA) with Perdew-Burke-Ernzerhof (PBE) exchange correlation. A cutoff energy of 544 eV while a 2×1×2 k-point mesh for ZUL-100 with smearing 0.5 eV were found to be enough for the total energy to converge within 1×10-6 eV atom^{-1}, the calculation error are within 0.001 Å. The structure of these materials would be first optimized by the Geometry Optimization implemented in the Materials Studio, CASTEP code. Then the isolated gas molecule was placed in the same cell dimensions as every sample crystal and was optimized and relaxed as references. CO_2, C_2H_2 and CH_4 gas molecules were finally introduced to different locations of the channel pore, followed by a full structural relaxation. The static adsorption enthalpy (at T = 0 K) was then calculated: ΔE = E (MOF) + E (gas) - E (MOF + gas).
The surface electrostatic potential of ZUL-100 was calculated using the Energy task in DMol3 code of Materials Studio, the Electron density and Electrostatics properties of the frame were calculated, with the density field being the total density and the potential field being the electrostatic potential field. The molecular surface electrostatic potential was calculated in Gaussian for CO_2, C_2H_2 and CH_4, respectively, using Multiwfn[7] for result analysis and the visualization was performed by VMD[8].

Breakthrough tests

The breakthrough experiments were carried out in a dynamic gas breakthrough equipment. All experiments were conducted using a stainlesss teel column (10 mm inner diameter × 50 mm) and the mass packed in the sample holder was: ZUL-100 (346.1 mg), SIFSIX-3-Cu (202.2 mg), SIFSIX-14-Cu-i (265.3 mg). Activated MOF powder was packed into stainless steel column under pure He atmosphere. The breakthrough experiments were carried out at flow rates as follows: 1 and 2 mL/min for CO_2/CH_4 50/50 (*v/v*) and C_2H_2/CH_4 50/50 (*v/v*) mixtures, respectively. Moreover, 2 mL/min for CO_2/CH_4 50/50 (*v/v*) mixture under 1000 ppm H_2O and 4 mL/min for CO_2/CH_4 15/85 (*v/v*) mixture. Outlet gas from the column was monitored using gas chromatography (GC-2010, SHIMADZU) with a thermal conductivity detector (TCD).
The captured gases during the breakthrough experiment can be recovered by desorption. When the breakthrough experiments were finished, the column was heated to 313/353 K and a flow rate of 10 mL min^{-1} He was introduced, and the outlet gas from the column was monitored using gas chromatography (GC-2010, SHIMADZU) with a thermal conductivity detector (TCD). The CO_2/C_2H_2 productivity from desorption was calculated as equation (10), and the cumulative purity p of CO_2/C_2H_2 obtained by desorption process was calculated using the equation (12) and equation (13):

$$n_i = \int_{t_1}^{t_2} F C_i dt \quad (9)$$

$$F = F_{He}/(1 - \sum_{i=1}^{n} c_i) \quad (10)$$

Where n_i was the eluted amount of the gas component i, F was the flow rate of the gas mixture which was calculated by the equation (11), n was the number of the gas component, C_i was the molar fraction of gas i in the outlet gas mixture.

$$p = \frac{n_{CO_2}}{n_{CO_2} + n_{CH_4}} \times 100\% \qquad (11)$$

$$p = \frac{n_{C_2H_2}}{n_{C_2H_2} + n_{CH_4}} \times 100\% \qquad (12)$$

Where n_{CO_2}, $n_{C_2H_2}$ and n_{CH_4} were calculated from the given time period corresponding to the desorption curve.

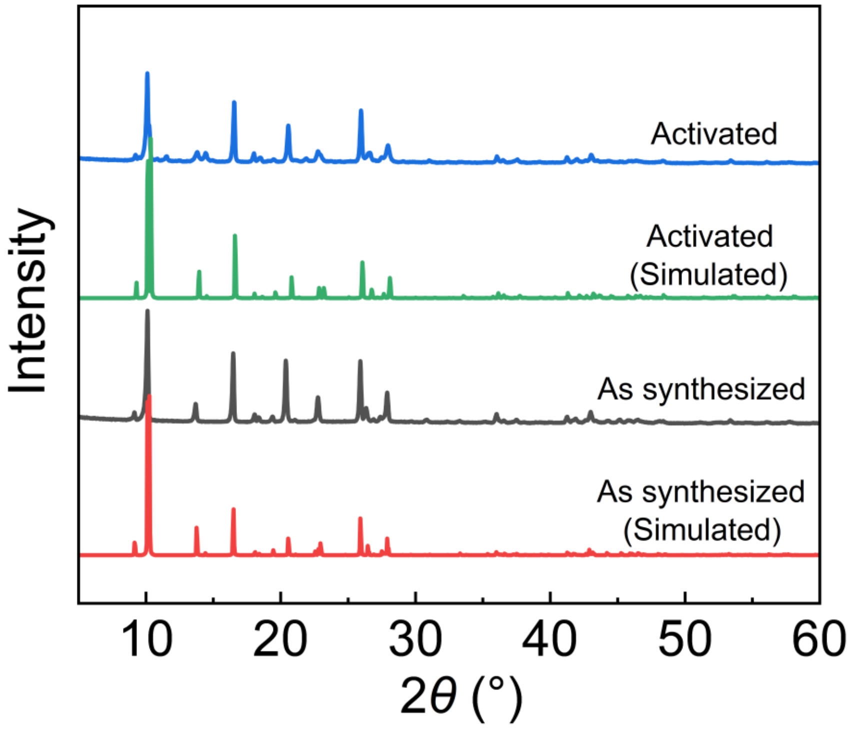

Figure S1. Powder X-ray diffraction patterns of ZUL-100.

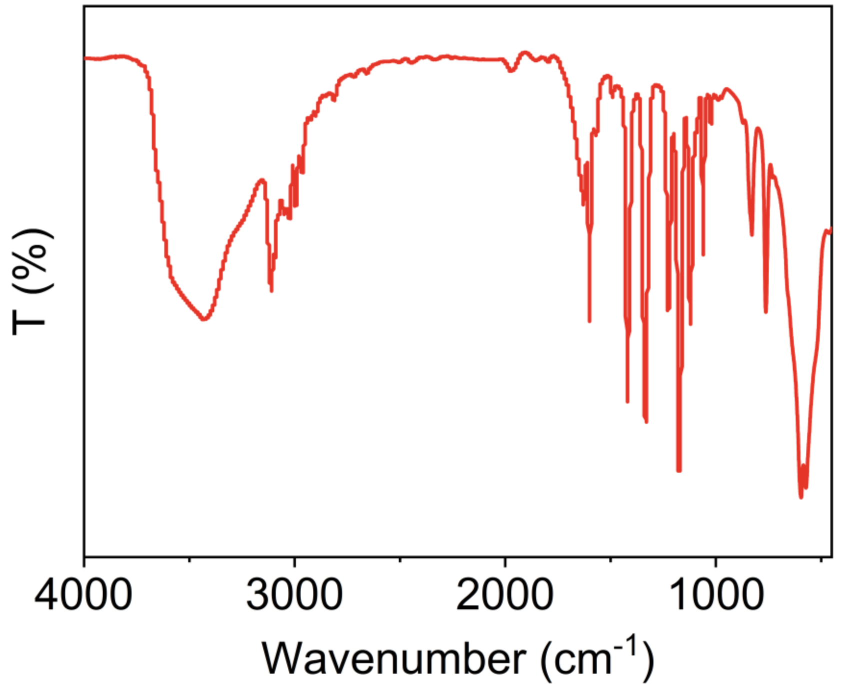

Figure S2. FTIR spectra of the activated ZUL-100 for powder samples.

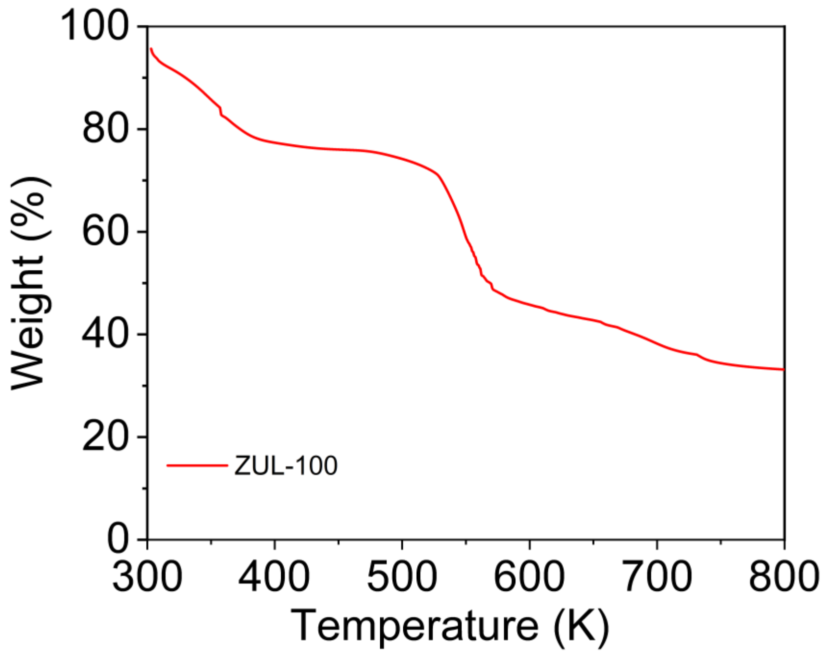

Figure S3. TGA curves of the activated ZUL-100 powder samples.

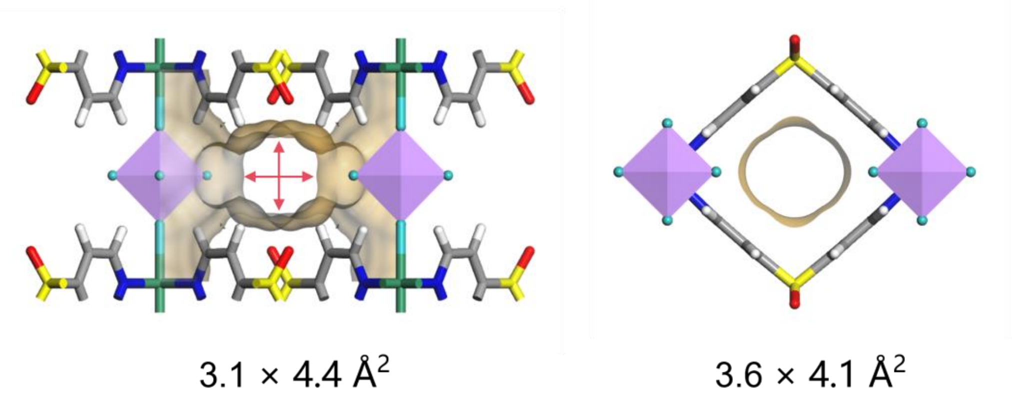

Figure S4. The activated structures of ZUL-100 (Color code: F cyan; C light gray; H white; N blue; Cu green;O red; S bright yellow; Ti purple).

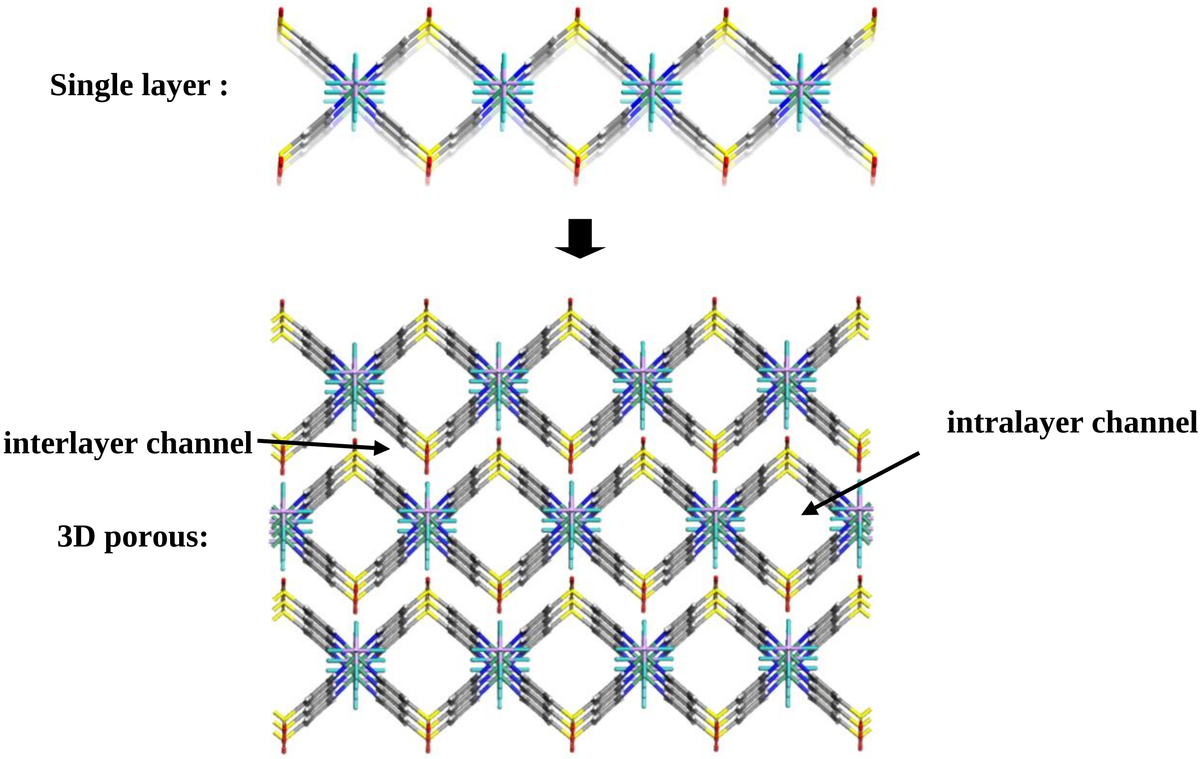

Figure S5. The activated structures of ZUL-100 (Color code: F cyan; C light gray; H white; N blue; Cu green;O red; S bright yellow; Ti purple).

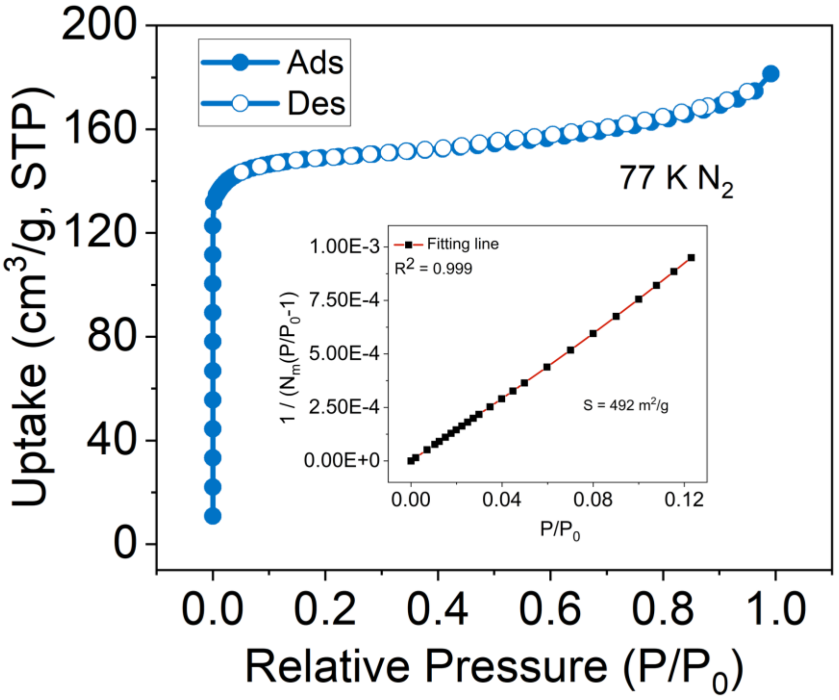

Figure S6. The N_2 adsorption isotherm on ZUL-100 at 77 K.

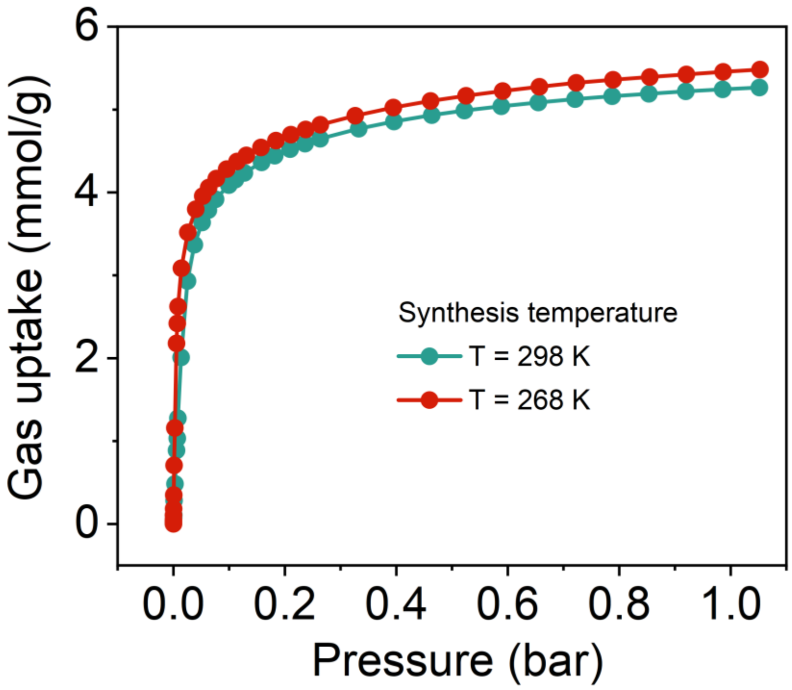

Figure S7. Adsorption isotherms of C_2H_2 on ZUL-100 with different synthesis temperature.

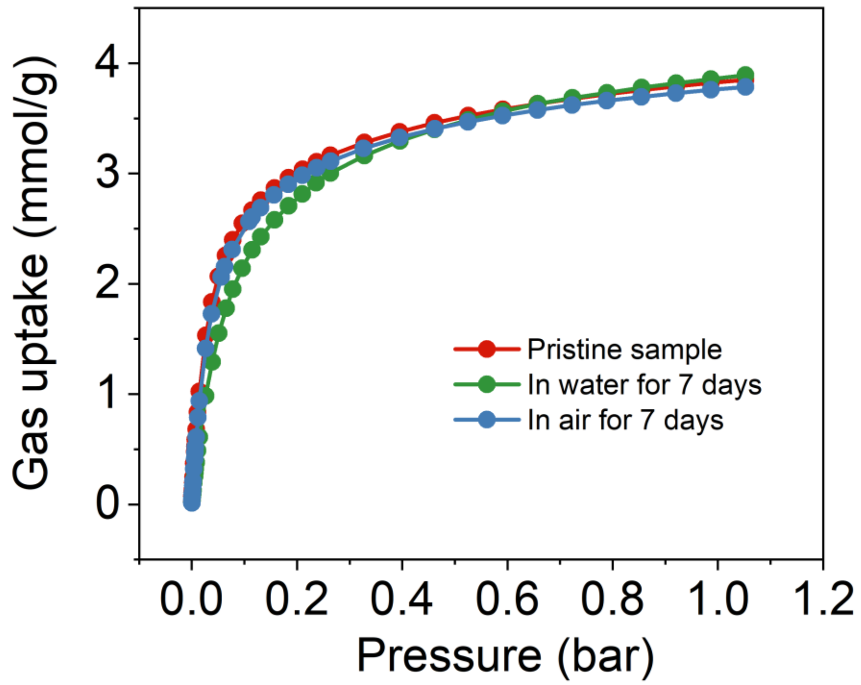

Figure S8. Adsorption isotherms of CO_2 on ZUL-100 at 298 K after exposure to air and immersing in water, respectively.

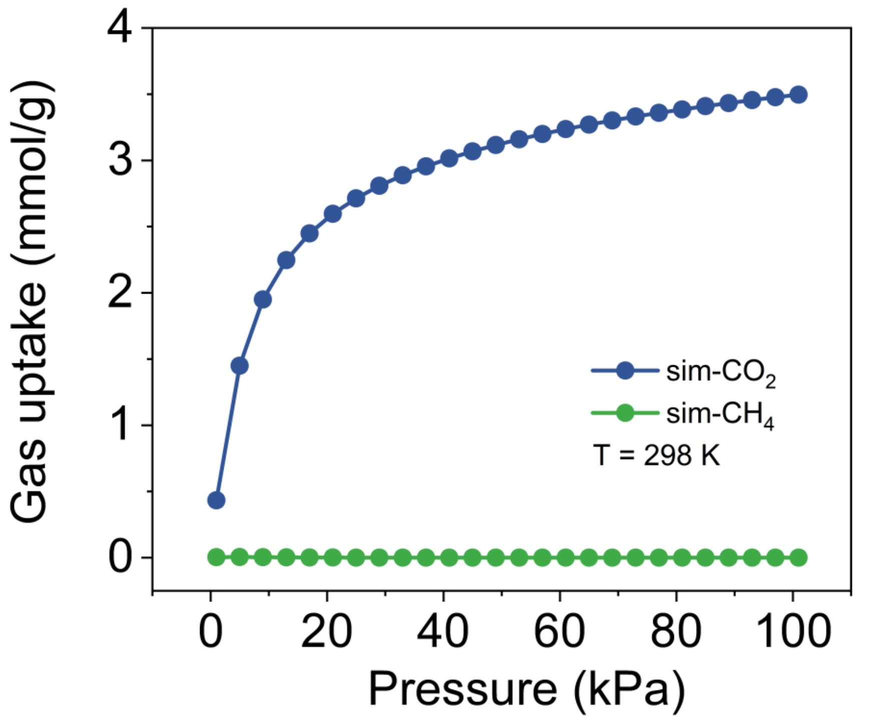

Figure S9. Competitive adsorption isotherms of CO_2 and CH_4 for CO_2/CH_4 50/50 (*v/v*) mixture on ZUL-100 at 298 K.

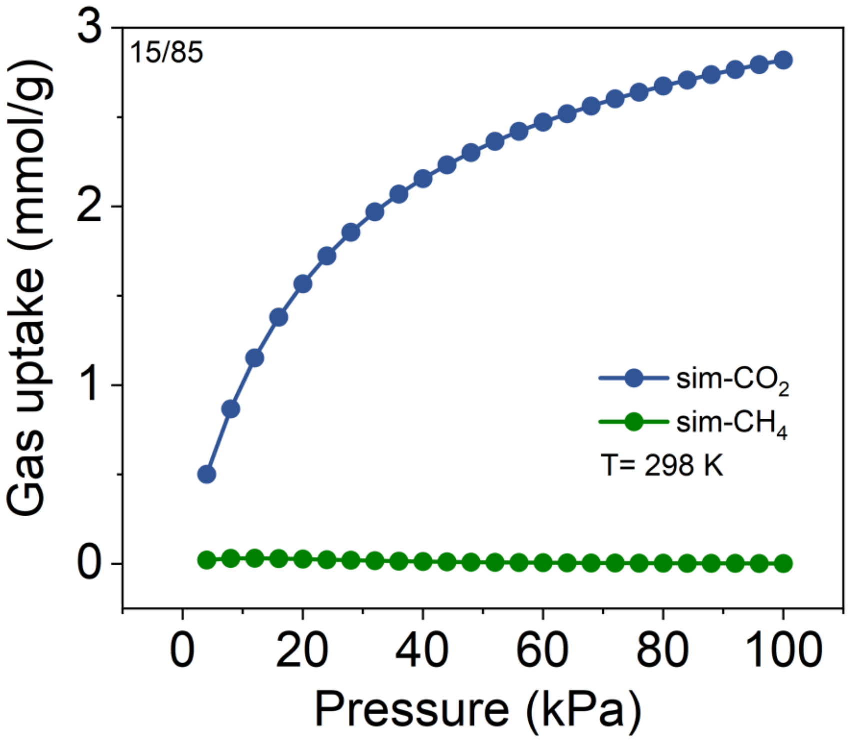

Figure S10. Competitive adsorption isotherms of CO_2 and CH_4 for CO_2/CH_4 15/85 (*v/v*) mixture on ZUL-100 at 298 K.

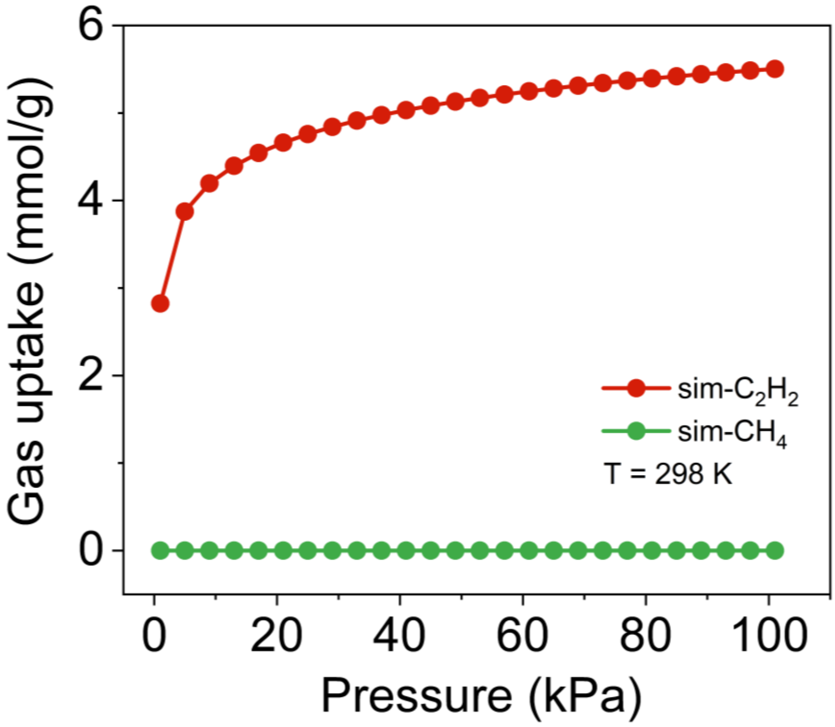

Figure S11. Competitive adsorption isotherms of C_2H_2 and CH_4 for C_2H_2/CH_4 50/50 (*v/v*) mixture on ZUL-100 at 298 K.

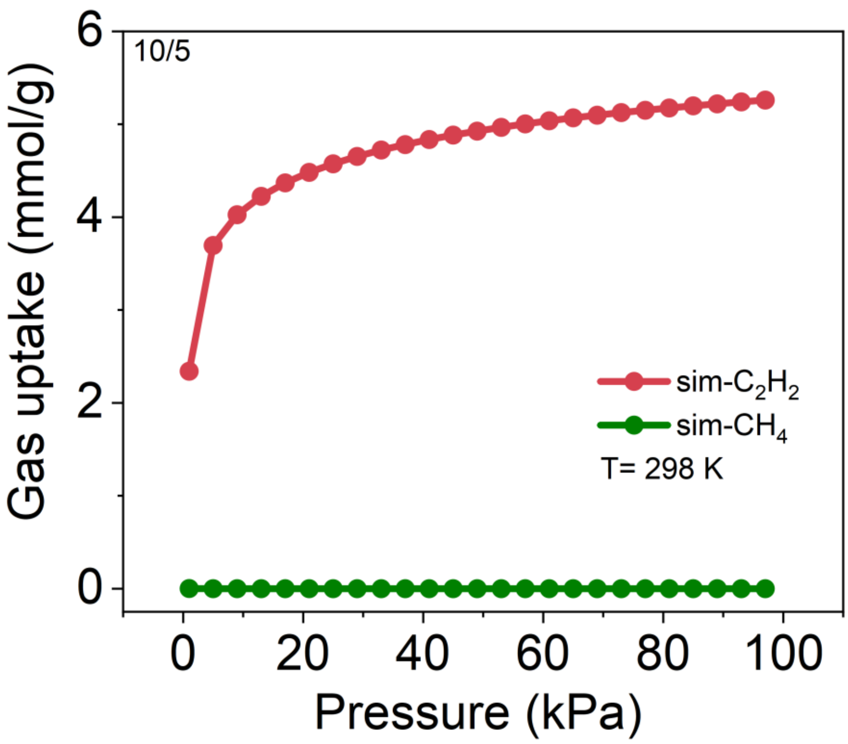

Figure S12. Competitive adsorption isotherms of C_2H_2 and CH_4 for C_2H_2/CH_4 10/5 (*v/v*) mixture on ZUL-100 at 298 K.

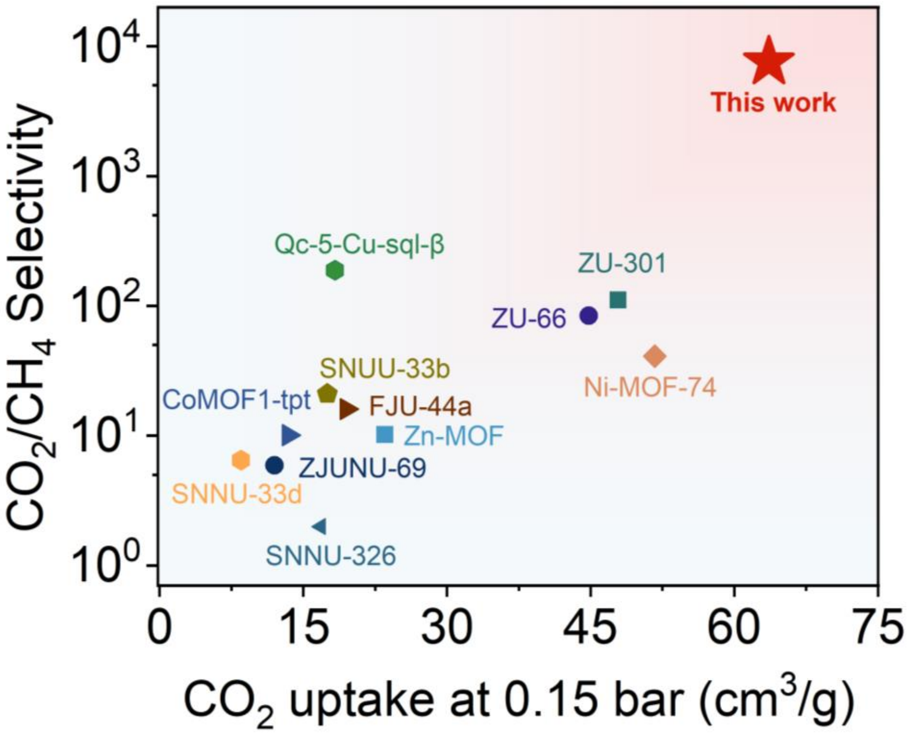

Figure S13. Comparison of the IAST selectivities and CO_2 adsorption uptakes at 0.15 bar and 298 K of ZUL-100 with representative porous materials.

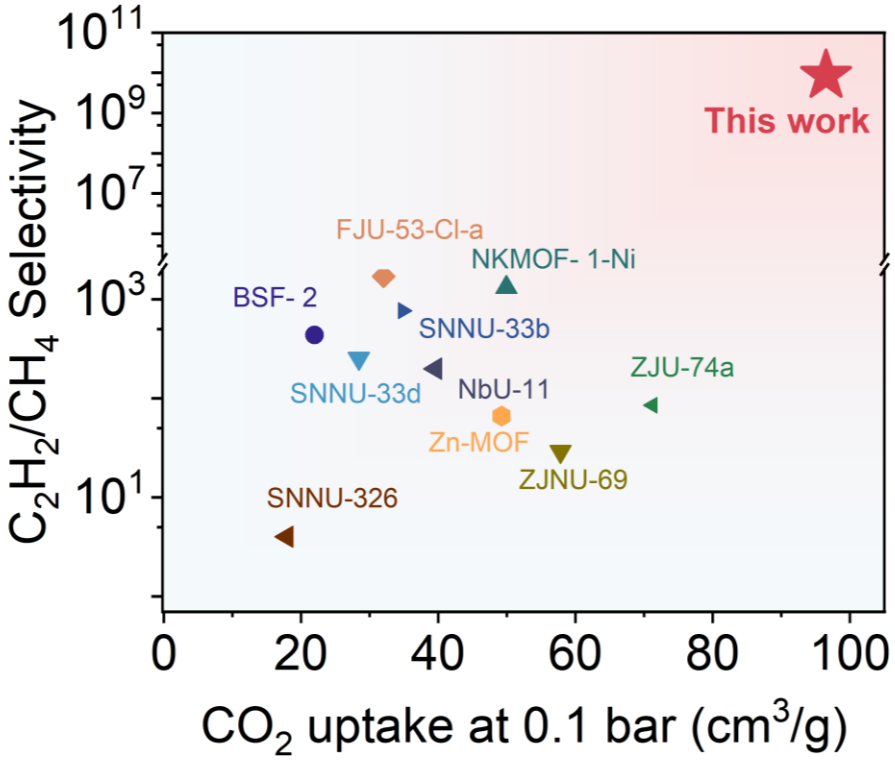

Figure S14. Comparison of the IAST selectivities and C_2H_2 adsorption uptakes at 0.1 bar and 298 K of ZUL-100 with representative porous materials.

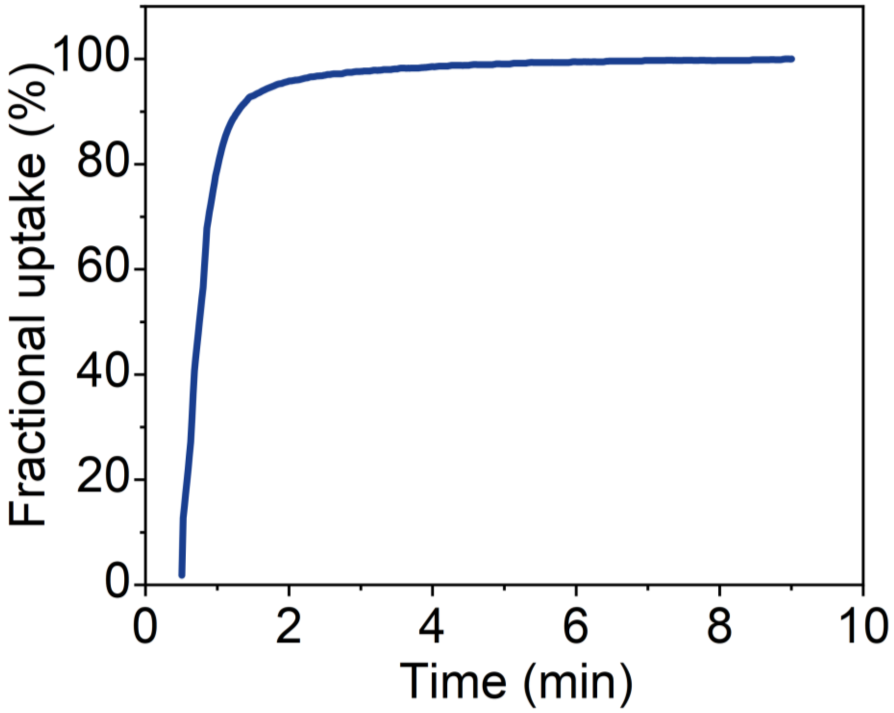

Figure S15. CO_2 kinetic adsorption curves of ZUL-100 at 0.5 bar and 298 K.

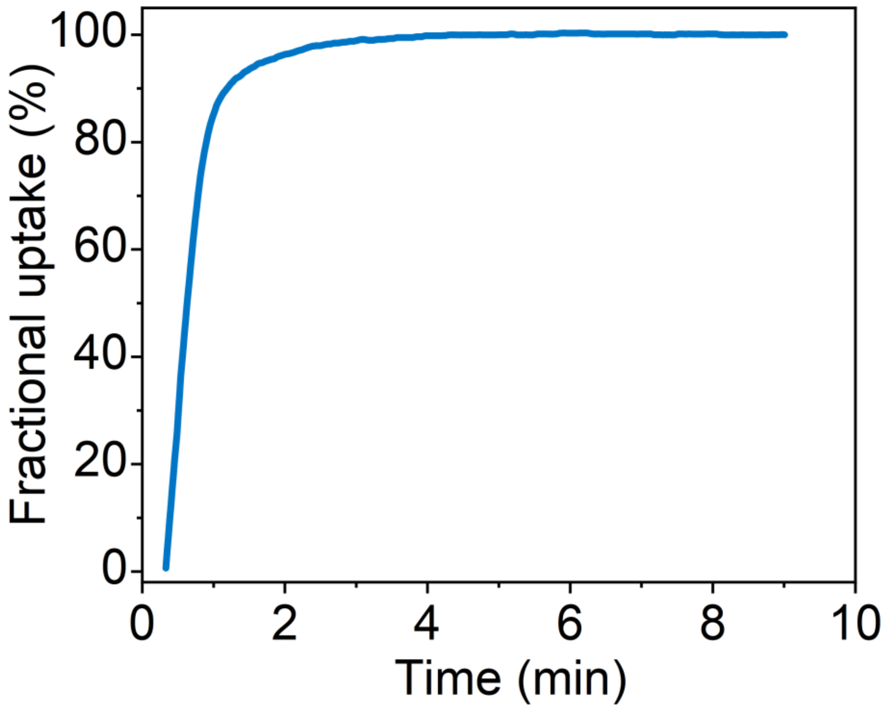

Figure S16. C_2H_2 kinetic adsorption curves of ZUL-100 at 0.5 bar and 298 K.

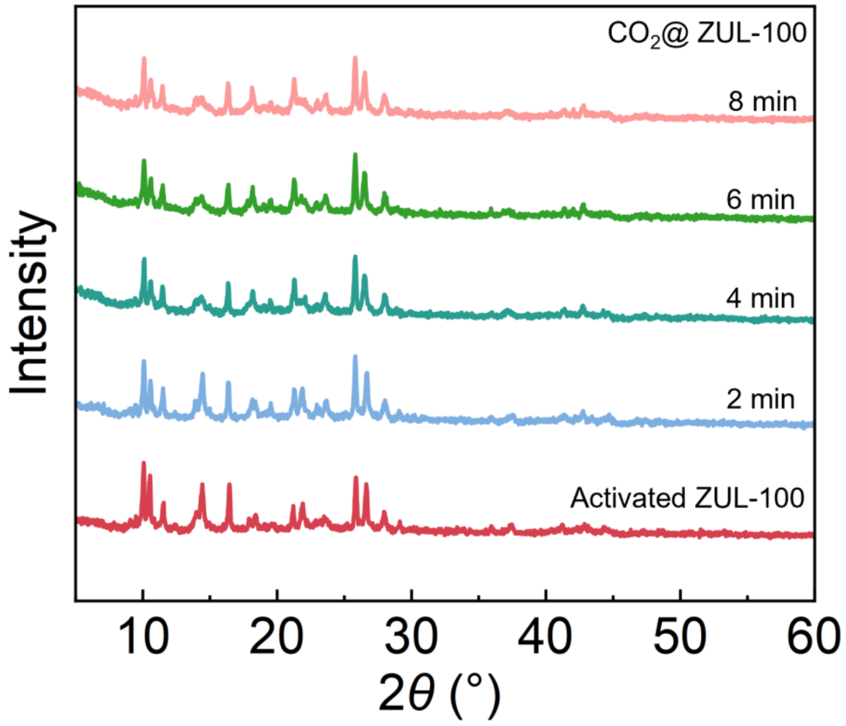

Figure 17. In-situ powder X-ray diffraction patterns of activated ZUL-100, CO_2@ZUL-100 at 1 bar.

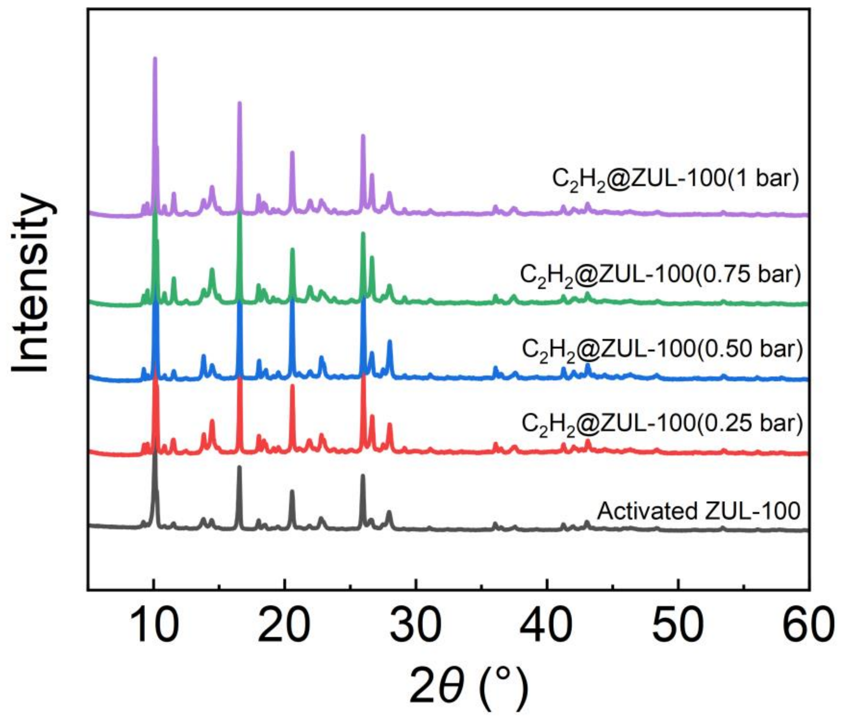

Figure 18. Powder X-ray diffraction patterns of activated ZUL-100, C_2H_2@ZUL-100 at 0.25, 0.5, 0.75 and 1 bar.

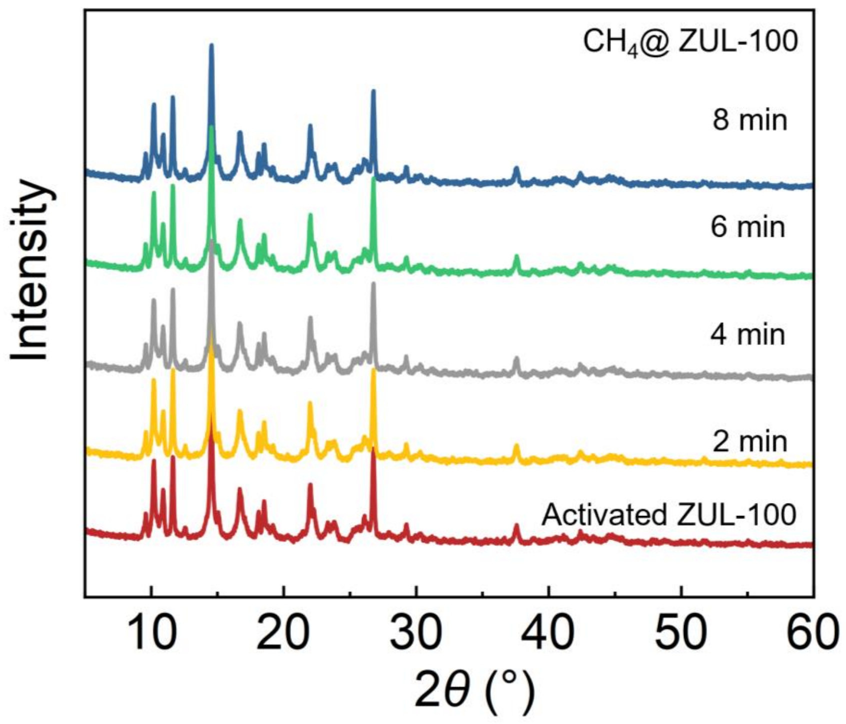

Figure S19. In-situ powder X-ray diffraction patterns of activated ZUL-100, CH_4@ZUL-100 at 1 bar.

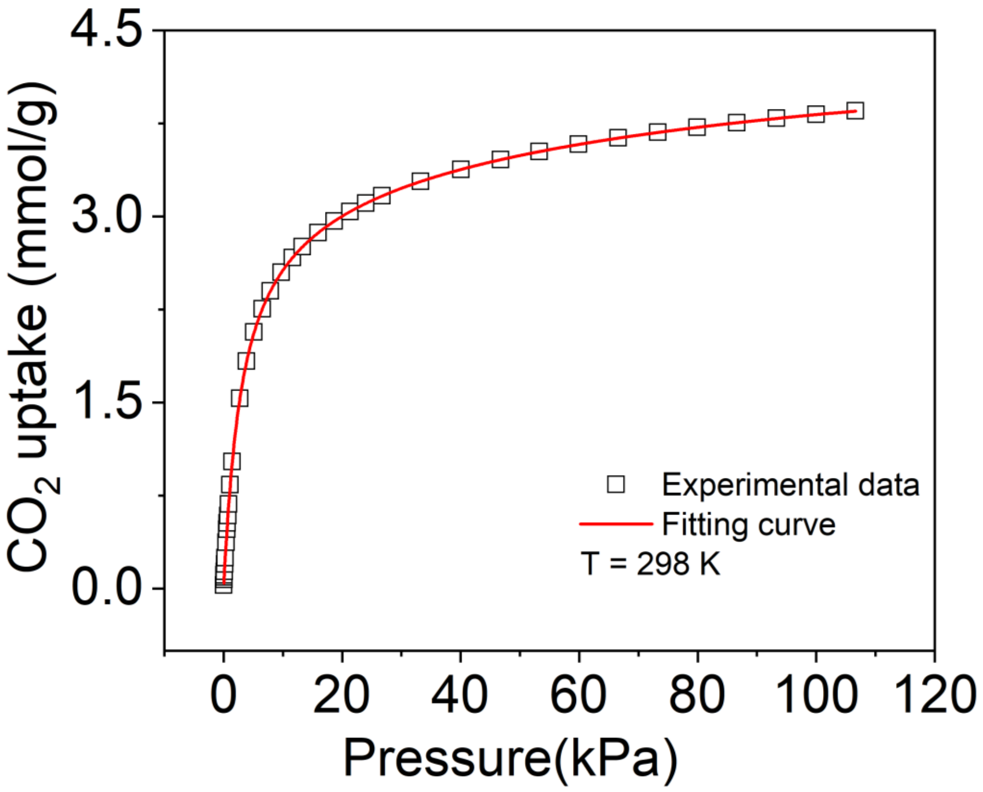

Figure S20. DSLF fitting of the CO_2 adsorption data at 298 K for ZUL-100.

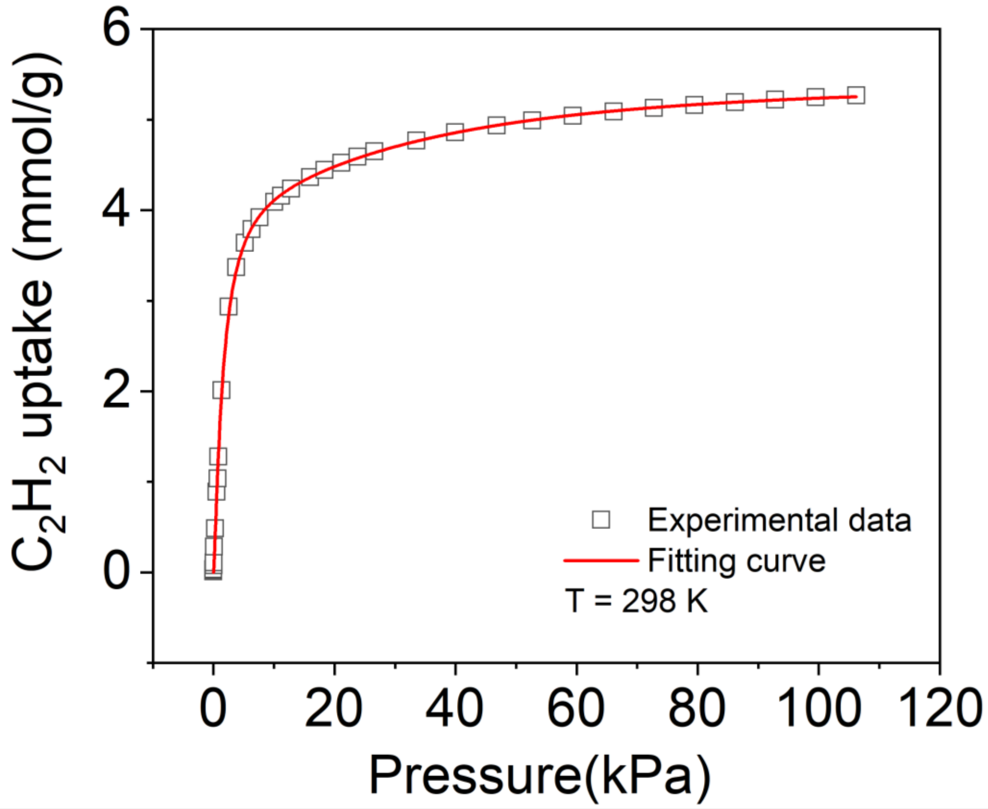

Figure S21. DSLF fitting of the C_2H_2 adsorption data at 298 K for ZUL-100.

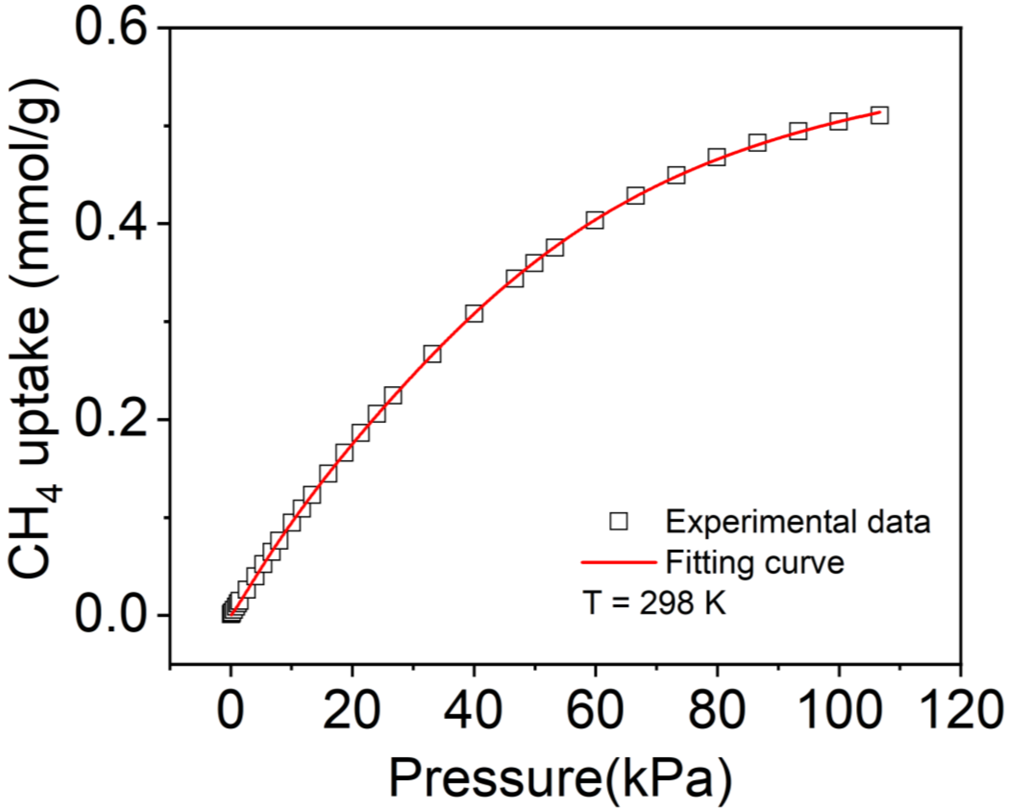

Figure S22. SSLF fitting of the CH_4 adsorption data at 298 K for ZUL-100.

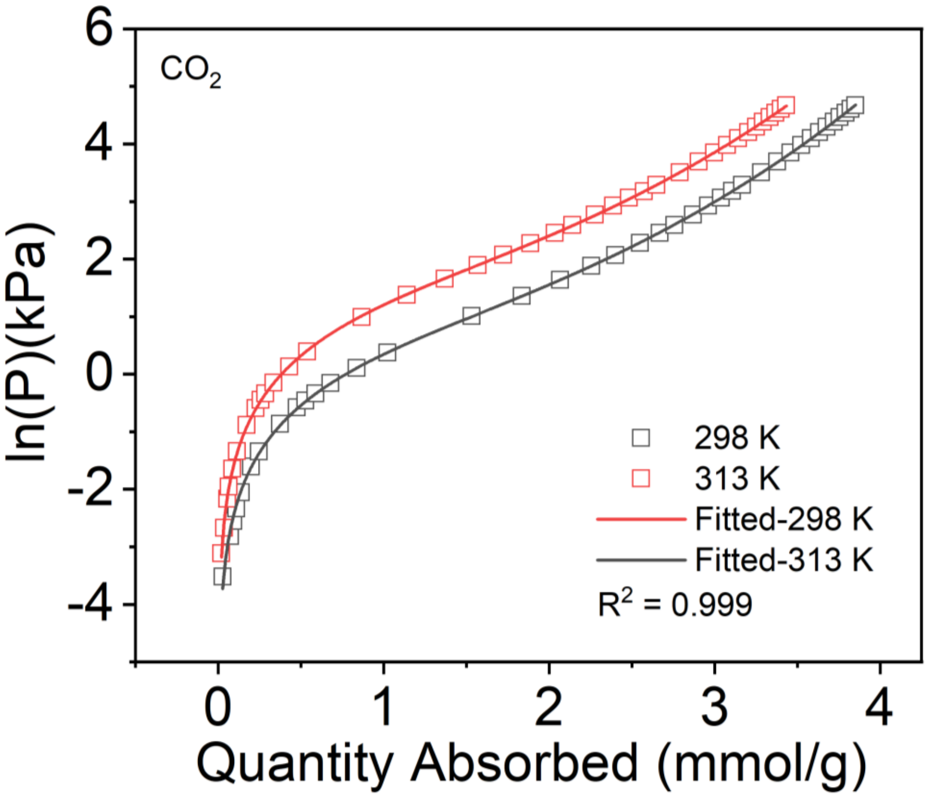

Figure S23. The Virial fitting of CO_2 adsorption data for ZUL-100.

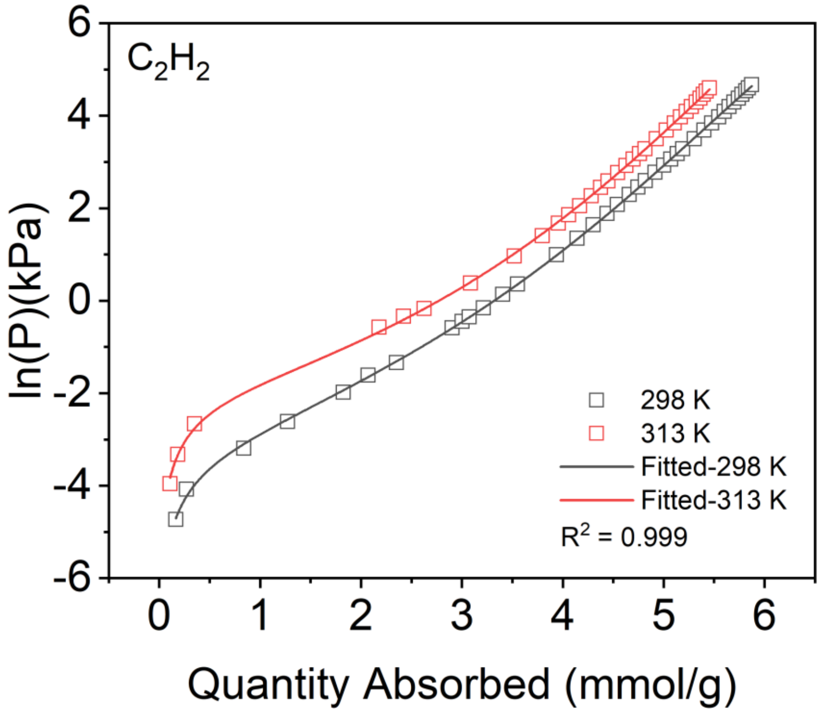

Figure S24. The Virial fitting of C_2H_2 adsorption data for ZUL-100.

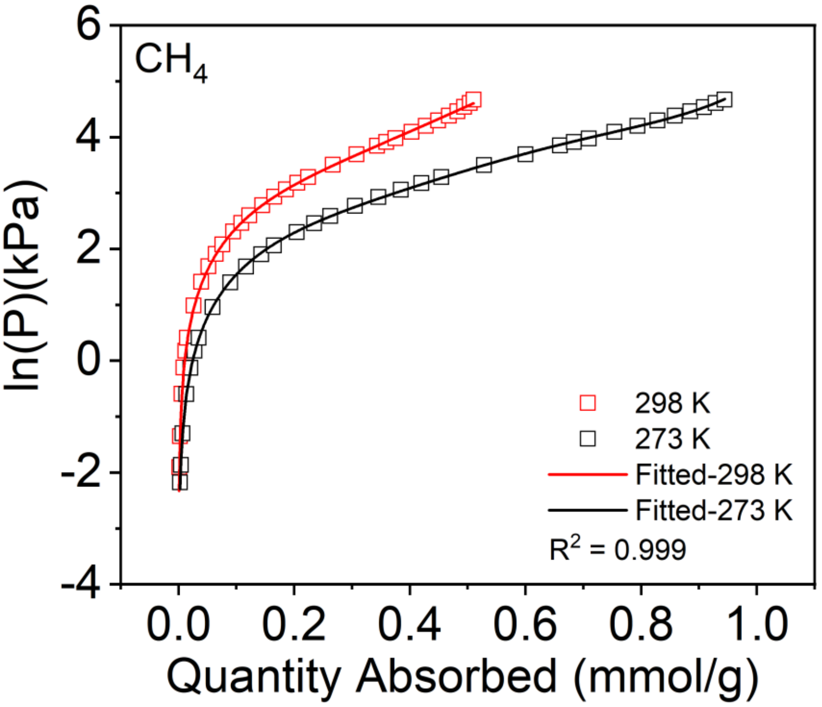

Figure S25. The Virial fitting of CH_4 adsorption data for ZUL-100.

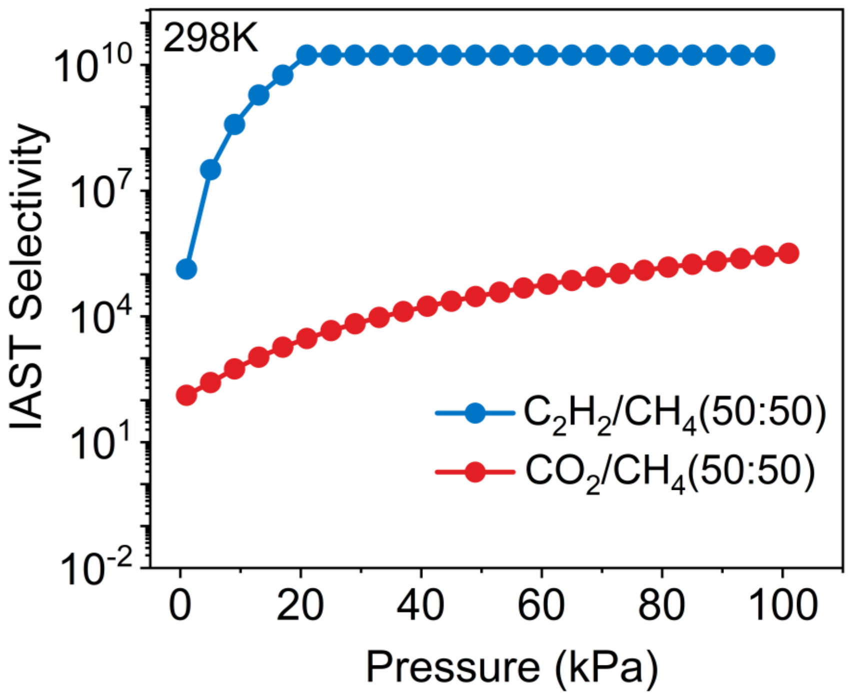

Figure S26. The IAST selectivities of ZUL-100 for CO_2/CH_4 (50/50, *v/v*) and C_2H_2/CH_4 (50/50, *v/v*) mixtures, respectively.

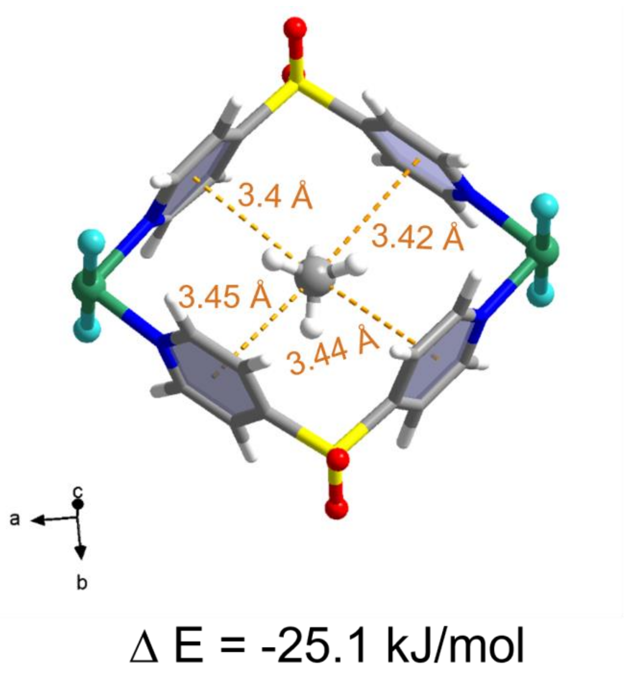

Figure S27. The DFT-D calculated binding site of CH_4 in activated ZUL-100. (Color code: F cyan; C light gray; H white; N blue; Cu green; O red; S bright yellow)

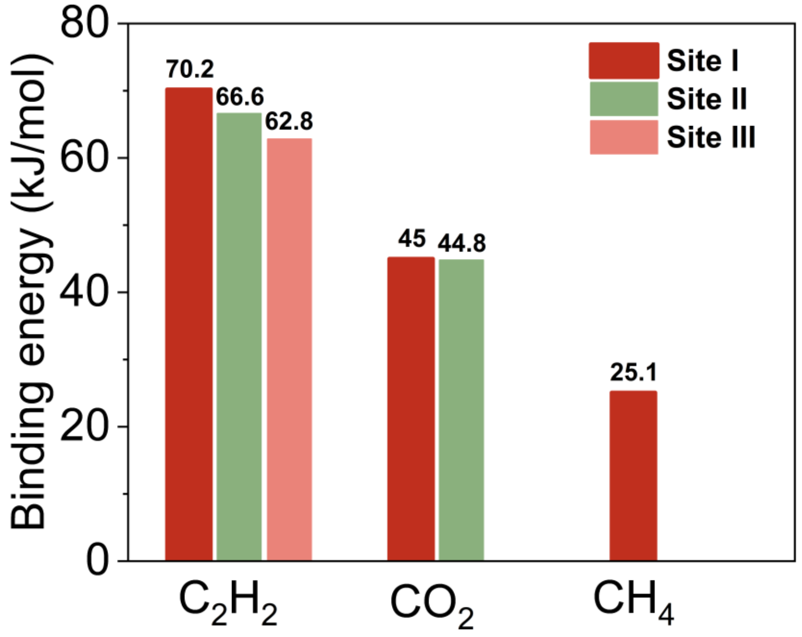

Figure S28. Binding energies of CO_2, C_2H_2 and CH_4 at different sites in activated ZUL-100.

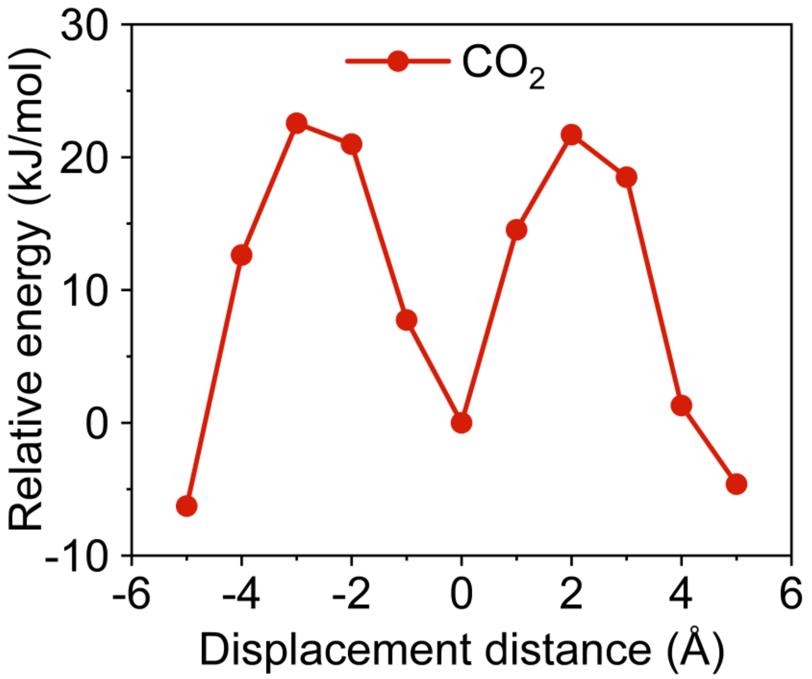

Figure S29. The relative energy change of CO_2 through the interlayer channel (within the ab plane) of ZUL-100.

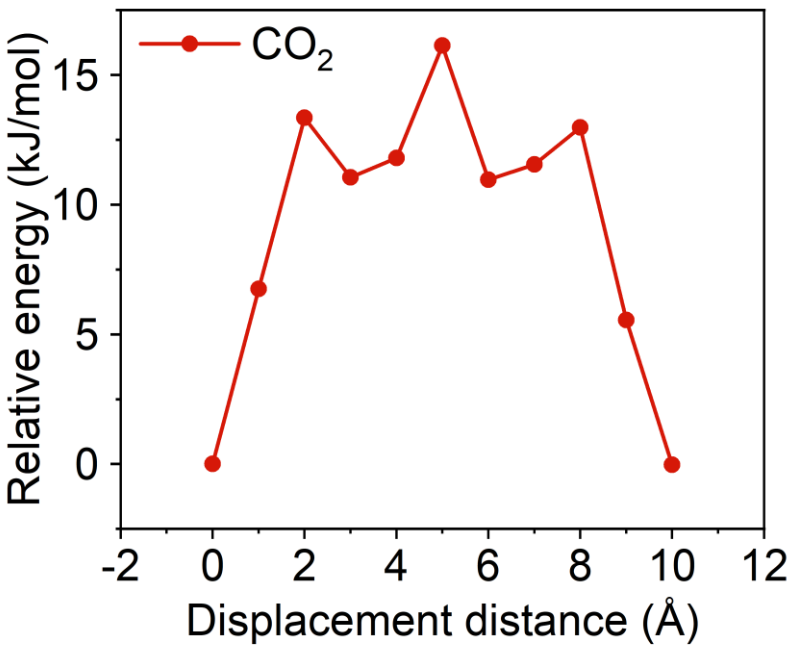

Figure S30. The relative energy change of CO_2 through the intralayer channel (along c-axis) of ZUL-100.

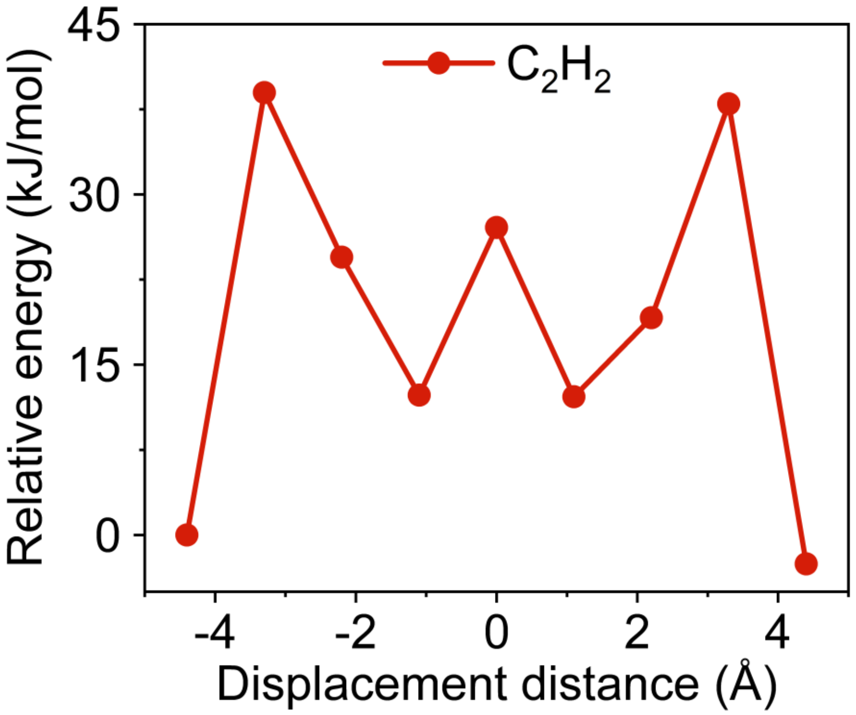

Figure S31. The relative energy change of C_2H_2 through the interlayer channel (within the ab plane) of ZUL-100.

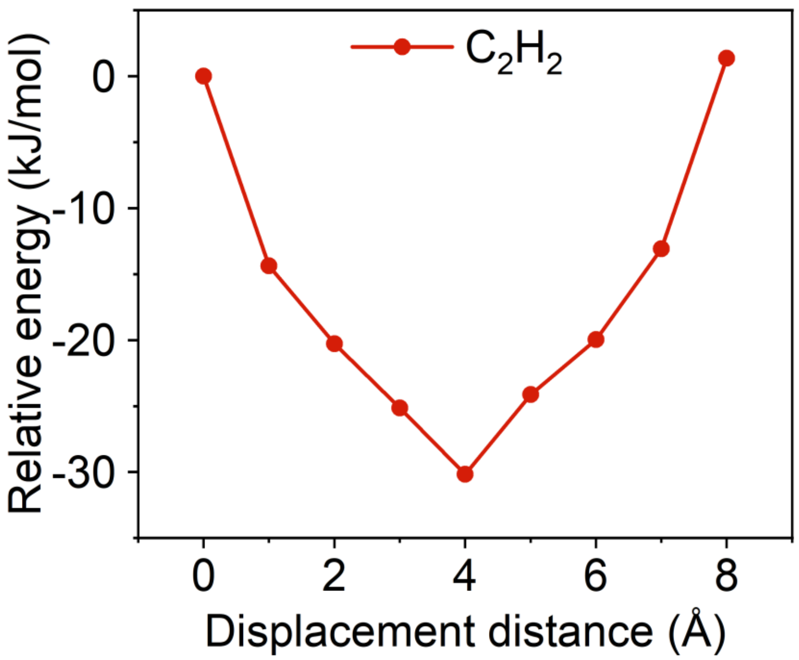

Figure S32. The relative energy change of C_2H_2 through the intralayer channel (along c-axis) of ZUL-100.

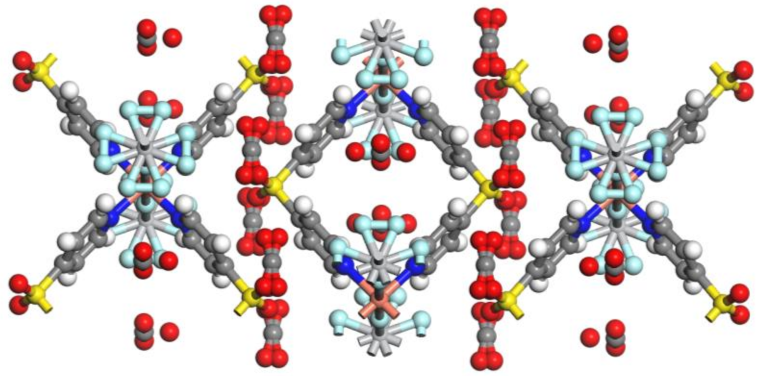

Figure S33. The single crystal structure of CO_2@ ZUL-100.

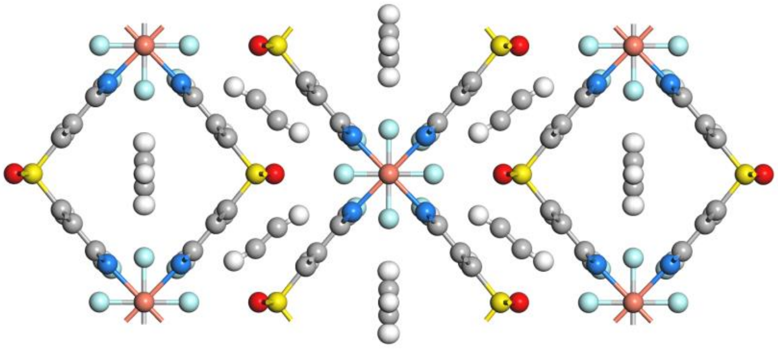

Figure S34. The single crystal structure of C_2H_2@ ZUL-100.

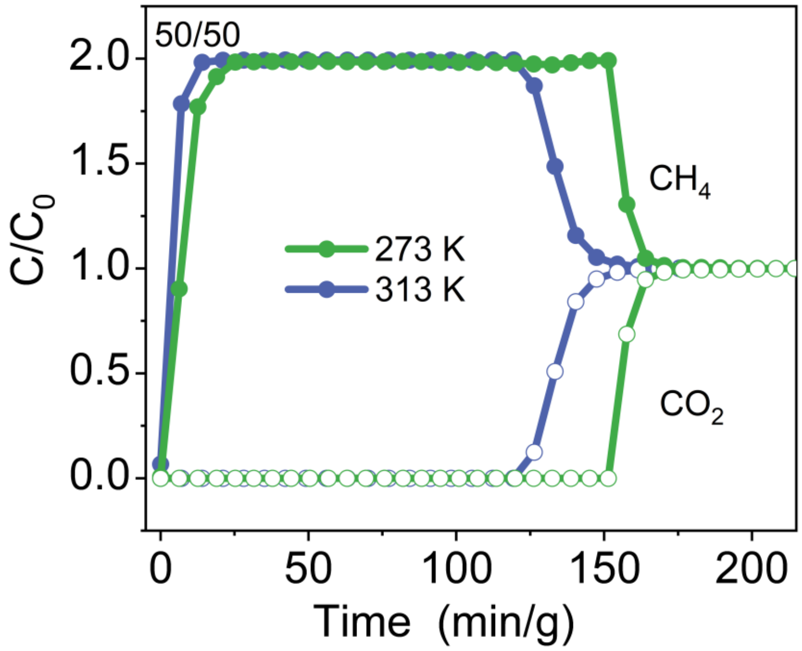

Figure S35. The experimental column breakthrough curves of 50/50 (v/v) CO_2/CH_4 gas mixture for ZUL-100 at 273/313 K and 1 bar with a flow rate of 1 mL/min.

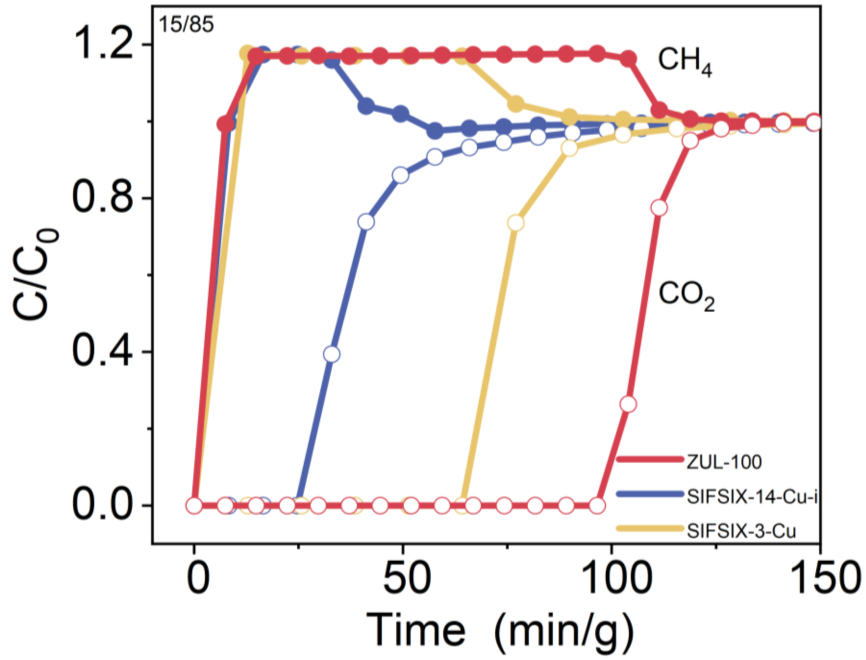

Figure S36. Breakthrough curves of CO_2/CH_4 (15/85, *v/v*) mixture on ZUL-100 (red), SIFSIX-3-Cu (yellow) and SIFSIX-14-Cu-i (blue) at 298 K with a flow rate of 4 mL/min.

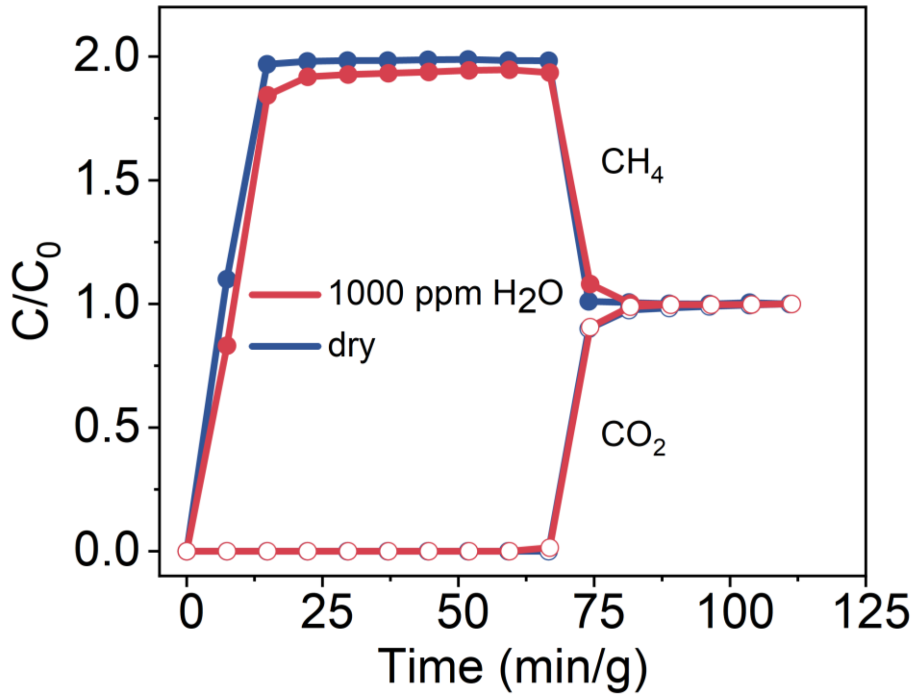

Figure S37. Breakthrough curves for ZUL-100 under flow of CO_2/ CH_4 (50/50) gas mixture in dry and humid (with 1000 ppm H_2O) conditions at 298 K and 1 bar.

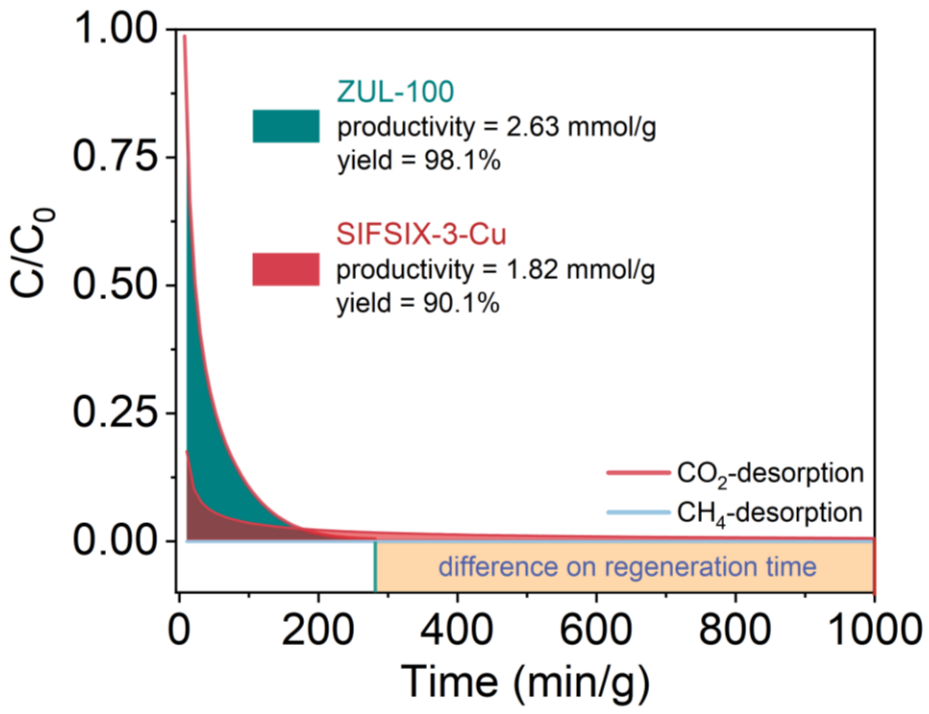

Figure S38. Desorption curves for saturated ZUL-100 and SIFSIX-3-Cu column of CO_2/CH_4 (15/85, *v*/*v*) mixture with He purge at 313 K with a flow rate of 10 mL/min.

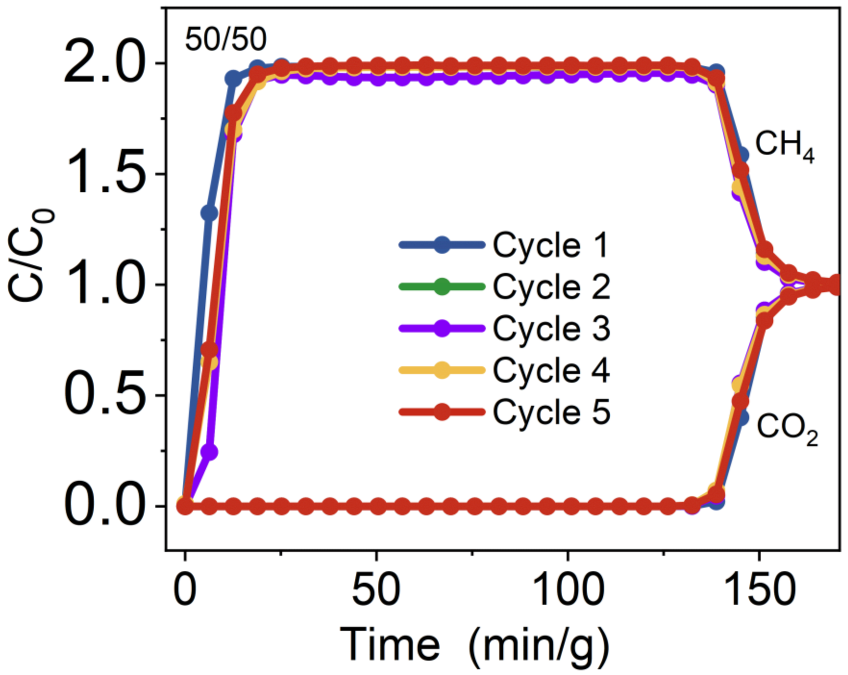

Figure S39. Cycling column breakthrough curves of 50/50 (v/v) CO_2/CH_4 gas mixture for ZUL-100 at 298 K and 1 bar with a flow rate of 1 mL/min.

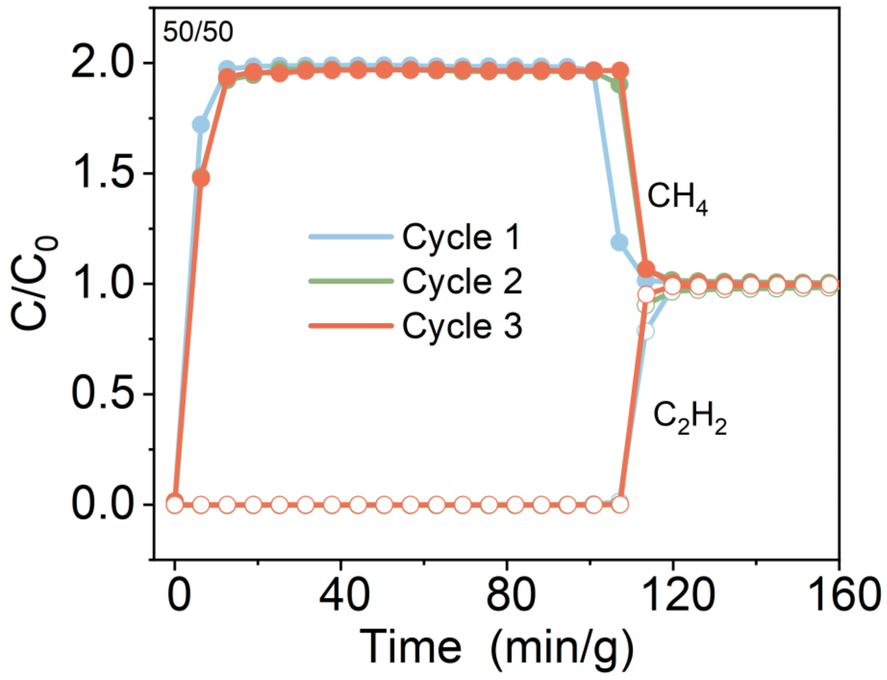

Figure S40. Cycling column breakthrough curves of 50/50 (v/v) C_2H_2/CH_4 gas mixture for ZUL-100 at 298 K and 1 bar with a flow rate of 2 mL/min.

Table S1. Property comparison of CO_2, C_2H_2 and CH_4.

Gas molecules	Kinetic Diameter (Å)	Molecular size (Å^3)	Boiling point (K)
CO_2	3.3	3.18 × 3.33 × 5.36	194.7
C_2H_2	3.3	3.32 × 3.34 × 5.70	188.1
CH_4	3.8	3.82 × 3.94 × 4.10	111.5

Table S2. Crystal data for the as-synthesized, the activated ZUL-100.

Unit cell parameters	As-synthesized ZUL-100	Activated ZUL-100
Formula sum	C_{20} H_{16} Cu Ti F_6 N_4 O_4 S_2	C_{20} H_{16} Cu Ti F_6 N_4 O_4 S_2
Formula weight	665.93	666.0
Crystal system	Orthorhombic	Orthorhombic
Space group	C m m m	C m m m
a/Å	9.8088	9.8284
b/Å	19.283	19.049
c/Å	8.6328	8.5345
Volume/Å^3	1632.83	1597.8
Z	2	2
Calc. density	1.3445 g/cm^3	1.384 g/cm^3

Table S3. Crystal data for the CO_2 and C_2H_2-loaded ZUL-100.

Unit cell parameters	The CO_2-loaded ZUL-100	The C_2H_2-loaded ZUL-100
Formula sum	$C_{21.20}$ H_{16} Cu Ti F_6 N_4 $O_{6.40}S_2$	C_{26} H_{22} Cu F_6 Ti N_4 O_4 S_2
Formula weight	718.72 g/mol	744.02 g/mol
Crystal system	Orthorhombic	Orthorhombic
Space group	C m m m	C m m m
a/Å	9.7457	9.8977
b/Å	19.293	18.8759
c/Å	8.2873	8.5038
Volume/Å^3	1558.2	1588.75
Z	2	2
Calc. density	1.532 g/cm^3	1.555

Table S4. Dual-Langmuir-Freundlich fitting parameters for C_2H_2 and CO_2 in ZUL-100 at 298 K.

298 K	Site 1			Site 2			R^2
	q_A mmol/g	b_A kPa^{-1}	v_A	q_B mmol/g	b_B kPa^{-1}	v_B	
CO_2	2.81	0.32	1.19	2.90	0.09	0.59	0.9999
C_2H_2	3.75	0.20	0.55	2.77	3.54	1.50	0.9997

Table S5. Single-Langmuir-Freundlich fitting parameters for CH_4 in ZUL-100 at 298 K.

298 K	Site 1			R^2
	q mmol/g	b kPa^{-1}	v	
CH_4	0.77	0.0096	1.15	0.9995

Table S6. Virial fitting parameters for C_2H_2 in ZUL-100 with the temperatures of 298 K and 313 K.

Equation	y=ln(x)+1/K*(a0+a1*x+a2*x^2+a3*x^3+a4*x^4+a5*x^5) + (b0+b1*x+ b2*x^2)			
Temperature	298 K		313 K	
Parameters	Value	Standard Error	Value	Standard Error
a0	-8185.56616	255.19237	-8185.56616	255.19237
a1	1745.99203	192.02748	1745.99203	192.02748
a2	-150.60283	62.55454	-150.60283	62.55454
a3	-23.86904	19.65994	-23.86904	19.65994
a4	4.36366	3.42423	4.36366	3.42423
a5	-0.31219	0.2181	-0.31219	0.2181
b0	24.62929	0.82185	24.62929	0.82185
b1	-6.25024	0.5604	-6.25024	0.5604
b2	0.91527	0.08876	0.91527	0.08876

Table S7. Virial fitting parameters for CO_2 in ZUL-100 with the temperatures of 298 K and 313 K.

Equation	y=ln(x)+1/K*(a0+a1*x+a2*x^2+a3*x^3+a4*x^4+a5*x^5) + (b0+b1*x+ b2*x^2)			
Temperature	298 K		313 K	
Parameters	Value	Standard Error	Value	Standard Error
a0	-5432.05232	123.00702	-5432.05232	123.00702
a1	347.44682	209.95606	347.44682	209.95606
a2	-256.52528	87.6912	-256.52528	87.6912
a3	78.57953	42.88429	78.57953	42.88429
a4	-12.10806	12.45187	-12.10806	12.45187
a5	0.78848	1.29023	0.78848	1.29023
b0	18.08103	0.40006	18.08103	0.40006
b1	-0.35321	0.67444	-0.35321	0.67444
b2	0.32004	0.19495	0.32004	0.19495

Table S8. Virial fitting parameters for CH_4 in ZUL-100 with the temperatures of 298 K and 313 K.

Equation	y=ln(x)+1/K*(a0+a1*x+a2*x^2+a3*x^3+a4*x^4+a5*x^5) + (b0+b1*x+ b2*x^2)			
Temperature	298 K		313 K	
Parameters	Value	Standard Error	Value	Standard Error
a0	-2820.14896	88.68387	-2820.14896	88.68387
a1	2047.93209	1003.76592	2047.93209	1003.76592
a2	-11254.49507	2303.37596	-11254.49507	2303.37596
a3	12610.57741	3724.87911	12610.57741	3724.87911
a4	-14094.05071	4675.67036	-14094.05071	4675.67036
a5	5757.15957	2065.43721	5757.15957	2065.43721
b0	13.9767	0.30878	13.9767	0.30878
b1	-4.1848	3.4426	-4.1848	3.4426
b2	23.59397	6.72853	23.59397	6.72853

Table S9. Summary of the separation performance for CO_2 and CH_4 in representative materials.

MOFs	0.5 bar CO_2 uptake (cm^3/g)	1 bar CO_2 uptake (cm^3/g)	CO_2/CH_4 selectivity (50/50) at 298 K and 1 bar	Q_{st} of CO_2 (kJ/mol)	Ref
**ZUL-100**	**78.4**	**85.6**	**3.2×10^5**	**45.2**	**This work**
SIFSIX-2-Cu-i	98.1	121.5	33	31.9	[9]
MOF 2	21.2	33.5	31.2	33.0	[10]
Ni-MOF-74	84.4	102.6	29.8	16.3	[11]
SNNU-14	21.3	33.6	18.7	42.6	[12]
FJU-44a	37.4	53.7	16	40.4	[13]
CoMOF1-tpt	35.8	63.7	14	27.6	[14]
SIFSIX-1-Cu	70.1	115.2	10.6	27	[15]
JLU-Liu66	29.2	44.8	9.4	29	[16]
JUC-141	64.1	79.9	8.72	27.9	[17]
MOF-505-K	27.8	45.0	5.5	32	[18]
zeolite 5A	89.7	93.8	23.5	-	[19]
NOTT-125	18.2	35.0	4.8	25.6	[20]
MOF 1	20.4	33.6	3.4	33.4	[10]
Qc-5-Cu-sql-β	39.2	48.59	3300	36	[21]
SIFSIX-14-Cu-i	76.6	96.1	-	37.7	[2]
SIFSIC-3-Cu	56.5	57.68	-	54	[1]
ZU-301	52.4	54.7	111	38.9	[22]
SIFSIX-3-Ni	53.5	55.1	134	48	[23]
ZU-16-Co	63.8	64.3	120	-	[24]
ZU-36-Ni	58.2	58.5	930	55.5	[25]
SNNU-33b	41.2	55.9	47	31.6	[26]
ZU-66	83.3	100.8	136	32	[27]

Table S10. Summary of the separation performance for C_2H_2 and CH_4 in representative materials.

MOFs	0.5 bar C_2H_2 uptake (cm^3/g)	1 bar C_2H_2 uptake (cm^3/g)	C_2H_2/CH_4 selectivity (50/50) at 298 K and 1 bar	Q_{st} of C_2H_2 (kJ/mol)	Ref
**ZUL-100**	**115.1**	**130.8**	**1.7×10^{10}**	**68.1**	**This work**
ZJU-74a	76.7	91.3	1312.9	45	[28]
ZJU-198a	73.5	98.4	391.1	26.1	[29]
MOF-2	59.8	79.3	332.3	49.8	[10]
Zr-SDBA	75.9	102.1	230.5	41.1	[30]
NbU-11	66.4	75.1	175.8	31.6	[31]
CuDTTA	42.3	50.3	166	39	[32]
JLU-MOF66	46.7	54.9	86.2	35.6	[16]
UTSA-50	71.0	114.3	68	39.4	[33]
BUT-70A	52.7	69.8	66.6	23.9	[34]
SNNU-5-FeTi	27.6	41.9	24.5	29.9	[35]
PCM-48	21.1	25.6	23.3	23	[36]
UTSA-200	76.8	81.5	16749114	39	[37]
SIFSIX-3-Ni	65.6	68.2	85	32	[23]
SIFSIX-2-Cu-i	82.2	90.4	124	37	[9]
SIFSIX-1-Cu	170.9	190.5	78	31	[15]
ZJNU-69	131.8	171.7	34.5	31.2	[38]
BSF-1	42.6	55	46.9	30.7	[39]
NKMOF-1-Ni	55.26	61	6409.1	60.3	[40]
BSF-2	40.64	51.2	324	37	[41]
SNNU-326	38.9	67.2	10.4	23.7	[42]
SNNU-33b	50.7	56.8	597.4	40.4	[26]
FJU-53-Cl-a	49.1	54.4	1306	38.1	[43]
$[Zn_3(SNDC)(AmTAZ)_3(H_2O)]\cdot H_2O\cdot CH_3CN$(Zn-MOF)	75.26	89.7	59	36.5	[44]

Table S11. Summary of the separation performance for CO_2 and CH_4 in representative materials.

MOFs	0.15 bar CO_2 uptake (cm^3/g)	CO_2/CH_4 selectivity (15/85) at 298 K and 1 bar	Ref
**ZUL-100**	**63.6**	**7555**	**This work**
Qc-5-Cu-sql-β	18.3	188	[21]
ZU-301	47.848	111	[22]
ZU-66	44.8	84	[27]
Ni-MOF-74	51.7	41	[11]
FJU-44a	19.5	16	[13]
CoMOF1-tpt	13.44	10.1	[14]
Zn-MOF	23.5	10.2	[44]
SNNU-326	16.8	2	[42]
SNNU-33d	8.5	6.5	[26]
SNUU-33b	17.5	21	[26]
ZJUNU-69	12	5.9	[38]

Table S12. Summary of the separation performance for C_2H_2 and CH_4 in representative materials.

MOFs	0.1 bar C_2H_2 uptake (cm^3/g)	C_2H_2/CH_4 selectivity (10/5, *v*/*v*) at 298 K and 1 bar	Ref
**ZUL-100**	**96.5**	**8.5×10^9**	**This work**
ZJU-74a	71.2	85	[28]
NKMOF- 1-Ni	49.9	1315	[40]
BSF- 2	21.952	434	[41]
FJU-53-Cl-a	32	1705	[43]
SNNU-326	17.92	4	[42]
SNNU-33b	34.8	767	[10]
SNNU-33d	28.4	253	[26]
CuDTTA	34.5	6476	[26]
Zn-MOF	49.2	66	[44]
ZJNU-69	57.8	29	[38]

Table S13. Comparison of C_2H_2 purity based on IAST simulation with ZUL-100 and representative porous materials for equimolar C_2H_2/CH_4 mixtures at 1 bar.

MOFs	C_2H_2 adsorbed phase uptake (mmol/g)	CH_4 adsorbed phase uptake (mmol/g)	Purity	Ref
**ZUL-100**	**5.5041**	**0.0000001**	**99.999**	**This work**
FJU-53-Cl-a	2.2170	0.0017	99.924	[43]
NKMOF-1-Ni	2.4893	0.0022	99.912	[40]
SNNU-33b	2.2584	0.0043	99.810	[26]
BSF-2	1.5034	0.0048	99.682	[41]
SNNU-33d	2.1202	0.0107	99.496	[26]
NbU-11	2.9788	0.0177	99.409	[31]
CuDTTA	1.8759	0.0112	99.408	[32]
SIFSIX-2-Cu-i	3.6643	0.0295	99.201	[9]
ZJU-74a	3.4051	0.0363	98.947	[28]
SIFSIX-3-Ni	2.8909	0.0340	98.839	[23]
$[Zn_3(SNDC)(AmTAZ)_3(H_2O)] \cdot H_2O \cdot CH_3CN$ (Zn-MOF)	3.3896	0.0572	98.339	[44]
SIFSIX-1-Cu	7.6349	0.1338	98.277	[15]
PCM-48	1.0864	0.0500	95.600	[36]
MOF-1	0.7504	0.0626	92.304	[10]
SNNU-326	1.6974	0.1635	91.216	[42]

Table S14. Comparison of breakthrough uptake for 50/50 CO_2/CH_4 gas mixtures among representative materials.

MOFs	CO_2 breakthrough uptake (mmol/g)	Ref
**ZUL-100**	**3.1**	**This work**
ZU-36-Ni	2.5	[25]
Qc-5-Cu-sql-β	2.2	[21]
SIFSIX-3-Cu	2.11	[1]
ZU-16-Co	1.78	[24]
ZU-301	1.76	[22]
MOF-2	1.7	[10]
SNNU-16	1.61	[12]
UTSA-120	1.61	[45]
SIXSIX-14-Cu-i	1.31	[2]
SNNU-36b	1.70	[26]
HOF-21	0.64	[46]

Table S15. Comparison of breakthrough uptake for 50/50 C_2H_2/CH_4 gas mixtures among representative materials.

MOFs	C_2H_2 breakthrough uptake (mmol/g)	Ref
**ZUL-100**	**4.79**	**This work**
MOF-2	2.23	[10]
SIFSIX-1-Cu	2.95	[15]
$[Zn_3(SNDC)(AmTAZ)_3(H_2O)]\cdot H_2O\cdot CH_3CN$ (Zn-MOF)	2.06	[44]
SIFSIX-2-Cu-i	2.02	[9]
SIFSIX-3-Ni	1.79	[23]
UTSA-200	3.35	[37]
SNNU-326	2.54	[42]
BSF-2	2.09	[41]
NKMOF-1-Ni	2.71	[40]
NbU-11	1.06	[31]
ZJU-74a	2.41	[28]
SNNU-36d	2.41	[26]
SNNU-36b	3.30	[26]

References

[1] Shekhah, O.; Belmabkhout, Y.; Chen, Z.; Guillerm, V.; Cairns, A.; Adil, K.; Eddaoudi, M. Made-to-Order Metal-Organic Frameworks for Trace Carbon Dioxide Removal and Air Capture. *Nat Commun* **2014**, *5* (1), 4228.

[2] Jiang, M.; Li, B.; Cui, X.; Yang, Q.; Bao, Z.; Yang, Y.; Wu, H.; Zhou, W.; Chen, B.; Xing, H. Controlling Pore Shape and Size of Interpenetrated Anion-Pillared Ultramicroporous Materials Enables Molecular Sieving of CO_2 Combined with Ultrahigh Uptake Capacity. *ACS Appl. Mater. Interfaces* **2018**, *10* (19), 16628–16635.

[3] Czepirski, L.; JagieŁŁo, J. Virial-Type Thermal Equation of Gas—Solid Adsorption. *Chemical Engineering Science* **1989**, *44* (4), 797–801

[4] Myers, A. L.; Prausnitz, J. M. Thermodynamics of Mixed-Gas Adsorption. *AIChE Journal* **1965**, *11* (1), 121–127.

[5] Segall, M. D.; Lindan, P. J. D.; Probert, M. J.; Pickard, C. J.; Hasnip, P. J.; Clark, S. J.; Payne, M. C. First-Principles Simulation: Ideas, Illustrations and the CASTEP Code. *J. Phys.: Condens. Matter* **2002**, *14* (11), 2717–2744.

[6] Ke, T.; Wang, Q.; Shen, J.; Zhou, J.; Bao, Z.; Yang, Q.; Ren, Q. Molecular Sieving of C2-C3 Alkene from Alkyne with Tuned Threshold Pressure in Robust Layered Metal–Organic Frameworks. *Angew. Chem. Int. Ed.* **2020**, *59* (31), 12725–12730.

[7] Lu, T.; Chen, F. Multiwfn: A Multifunctional Wavefunction Analyzer. *J Comput Chem* **2012**, *33* (5), 580–592.

[8] Humphrey, W.; Dalke, A.; Schulten, K. VMD: Visual Molecular Dynamics. *Journal of Molecular Graphics* **1996**, *14* (1), 33–38.

[9] Nugent, P.; Belmabkhout, Y.; Burd, S. D.; Cairns, A. J.; Luebke, R.; Forrest, K.; Pham, T.; Ma, S.; Space, B.; Wojtas, L.; Eddaoudi, M.; Zaworotko, M. J. Porous Materials with Optimal Adsorption Thermodynamics and Kinetics for CO_2 Separation. *Nature* **2013**, *495* (7439), 80–84.

[10] Feng, M.; Zhou, P.; Wang, J.; Wang, X.; Wang, D.; Li, C. Two Solvent-Induced In(III)-Based Metal–Organic Frameworks with Controllable Topology Performing High-Efficiency Separation of C_2H_2/CH_4 and CO_2/CH_4. *Inorg. Chem.* **2022**, *61* (29), 11057–11065.

[11] Chen, D.-L.; Shang, H.; Zhu, W.; Krishna, R. Reprint of: Transient Breakthroughs of CO_2/CH_4 and C_3H_6/C_3H_8 Mixtures in Fixed Beds Packed with Ni-MOF-74. *Chemical Engineering Science* **2015**, *124*, 109–117.

[12] Li, H.-P.; Dou, Z.-D.; Wang, Y.; Xue, Y. Y.; Li, Y. P.; Hu, M.-C.; Li, S.-N.; Jiang, Y.-C.; Zhai, Q.-G. Tuning the Pore Surface of an Ultramicroporous Framework for Enhanced Methane and Acetylene Purification Performance. *Inorg. Chem.* **2020**, *59* (22), 16725–16736.

[13] Ye, Y.; Zhang, H.; Chen, L.; Chen, S.; Lin, Q.; Wei, F.; Zhang, Z.; Xiang, S. Metal–Organic Framework with Rich Accessible Nitrogen Sites for Highly Efficient CO_2 Capture and Separation. *Inorg. Chem.* **2019**, *58* (12), 7754–7759.

[14] Gao, Q.; Zhao, X.-L.; Chang, Z.; Xu, J.; Bu, X.-H. Structural Stabilization of a Metal–Organic Framework for Gas Sorption Investigation. *Dalton Trans.* **2016**, *45* (16), 6830–6833.

[15] Burd, S. D.; Ma, S.; Perman, J. A.; Sikora, B. J.; Snurr, R. Q.; Thallapally, P. K.; Tian, J.; Wojtas, L.; Zaworotko, M. J. Highly Selective Carbon Dioxide Uptake by [Cu(Bpy-n)$_2$(SiF$_6$)] (Bpy-1 = 4,4′-Bipyridine; Bpy-2 = 1,2-Bis(4-Pyridyl)Ethene). *J. Am. Chem. Soc.* **2012**, *134* (8), 3663–3666.

[16] Kan, L.; Li, G.; Liu, Y. Highly Selective Separation of C_3H_8 and C_2H_2 from CH_4 within Two Water-Stable Zn5 Cluster-Based Metal–Organic Frameworks. *ACS Appl. Mater. Interfaces* **2020**, *12* (16), 18642–18649.

[17] Zhao, N.; Sun, F.; Li, P.; Mu, X.; Zhu, G. An Amino-Coordinated Metal–Organic Framework for Selective Gas Adsorption. *Inorg. Chem.* **2017**, *56* (12), 6938–6942.

[18] Chen, Y.; Wu, H.; Liu, Z.; Sun, X.; Xia, Q.; Li, Z. Liquid-Assisted Mechanochemical Synthesis of Copper Based MOF-505 for the Separation of CO_2 over CH_4 or N_2. *Ind. Eng. Chem. Res.* **2018**, *57* (2), 703–709.

[19] Saha, D.; Bao, Z.; Jia, F.; Deng, S. Adsorption of CO_2, CH_4, N_2O, and N_2 on MOF-5, MOF-177, and Zeolite 5A. *Environ. Sci. Technol.* **2010**, *44* (5), 1820–1826.

[20] Alsmail, N. H.; Suyetin, M.; Yan, Y.; Cabot, R.; Krap, C. P.; Lü, J.; Easun, T. L.; Bichoutskaia, E.; Lewis, W.; Blake, A. J.; Schröder, M. Analysis of High and Selective Uptake of CO_2 in an Oxamide-Containing {$Cu_2(OOCR)_4$}-Based Metal–Organic Framework. *Chemistry – A European Journal* **2014**, *20* (24), 7317–7324.

[21] Chen, K.; Madden, D. G.; Pham, T.; Forrest, K. A.; Kumar, A.; Yang, Q.; Xue, W.; Space, B.; Perry, J. J.; Zhang, J.; Chen, X.; Zaworotko, M. J. Tuning Pore Size in Square-Lattice Coordination Networks for Size-Selective Sieving of CO_2. *Angew Chem Int Ed* **2016**, *55* (35), 10268–10272.

[22] Yu, C.; Ding, Q.; Hu, J.; Wang, Q.; Cui, X.; Xing, H. Selective Capture of Carbon Dioxide from Humid Gases over a Wide Temperature Range Using a Robust Metal–Organic Framework. *Chemical Engineering Journal* **2021**, *405*, 126937.

[23] Shekhah, O.; Belmabkhout, Y.; Adil, K.; Bhatt, P. M.; Cairns, A. J.; Eddaoudi, M. A Facile Solvent-Free Synthesis Route for the Assembly of a Highly CO_2 Selective and H_2S Tolerant NiSIFSIX Metal–Organic Framework. *Chem. Commun.* **2015**, *51* (71), 13595–13598.

[24] Zhang, Z.; Ding, Q.; Cui, J.; Cui, X.; Xing, H. High and Selective Capture of Low-Concentration CO_2 with an Anion-Functionalized Ultramicroporous Metal-Organic Framework. *Sci. China Mater.* **2021**, *64* (3), 691–697.

[25] Zhang, Z.; Ding, Q.; Peh, S. B.; Zhao, D.; Cui, J.; Cui, X.; Xing, H. Mechano-Assisted Synthesis of an Ultramicroporous Metal–Organic Framework for Trace CO2 Capture. *Chem. Commun.* **2020**, *56* (56), 7726–7729.

[26] Xue, Y.; Lei, J.; Lv, H.; Liang, P.; Li, L.; Zhai, Q. Spatially Confined π-Complexation within Pore-Space-Partitioned Metal–Organic Frameworks for Enhanced Light Hydrocarbon Separation and Purification. *Small* **2024**, DOI:10.1002/smll.202311555.

[27] Yang, L.; Cui, X.; Zhang, Y.; Wang, Q.; Zhang, Z.; Suo, X.; Xing, H. Anion Pillared Metal–Organic Framework Embedded with Molecular Rotors for Size-Selective Capture of CO_2 from CH_4 and N_2. *ACS Sustainable Chem. Eng.* **2019**, *7* (3), 3138–3144.
[28] Pei, J.; Shao, K.; Wang, J.; Wen, H.; Yang, Y.; Cui, Y.; Krishna, R.; Li, B.; Qian, G.A Chemically Stable Hofmann-Type Metal−Organic Framework with Sandwich-Like Binding Sites for Benchmark Acetylene Capture. *Advanced Materials* **2020**, *32* (24), 1908275.
[29] Zhang, L.; Cui, X.; Xing, H.; Yang, Y.; Cui, Y.; Chen, B.; Qian, G. An Amino-Coordination Metal–Organic Framework for Highly Selective C_2H_2/CH_4 and C_2H_2/C_2H_4 Separations through the Appropriate Control of Window Sizes. *RSC Adv.* **2017**, *7* (34), 20795–20800.
[30] Gu, J.; Sun, X.; Kan, L.; Qiao, J.; Li, G.; Liu, Y. Structural Regulation and Light Hydrocarbon Adsorption/Separation of Three Zirconium–Organic Frameworks Based on Different V-Shaped Ligands. *ACS Appl. Mater. Interfaces* **2021**, *13* (35), 41680–41687.
[31] Wu, N.; Li, Q.; Li, J.; Wu, D.; Li, Y. 4-Connected Cobalt-Based 3D Framework with a High Affinity for Acetylene. *Inorg. Chem.* **2020**, *59* (14), 9461–9464.
[32] Wu, L.; Feng, M.; Zhang, Y.; Cao, Y.; Wang, D.; Li, C. Synergistic Effect of Active Sites and a Multiple-Micropore System for a Metal–Organic Framework Exhibiting High Separation of CO_2/CH_4 and C_2H_2/CH_4. *Inorg. Chem.* **2021**, *60* (16), 12151–12157.
[33] Xu, H.; He, Y.; Zhang, Z.; Xiang, S.; Cai, J.; Cui, Y.; Yang, Y.; Qian, G.; Chen, B. *J. Mater. Chem. A* **2012**, *1* (1), 77–81.
[34] Guo, Z.-J.; Yu, J.; Zhang, Y.-Z.; Zhang, J.; Chen, Y.; Wu, Y.; Xie, L.-H.; Li, J.-R. Water-Stable In(III)-Based Metal–Organic Frameworks with Rod-Shaped Secondary Building Units: Single-Crystal to Single-Crystal Transformation and Selective Sorption of C_2H_2 over CO_2 and CH_4. *Inorg. Chem.* **2017**, *56* (4), 2188–2197.
[35] Xue, Y.-Y.; Zhang, J.-W.; Li, Y.-P.; Li, H.-P.; Wang, Y.; Li, S.-N.; Jiang, Y.-C.; Hu, M.-C.; Zhai, Q.-G.Mimic of Ferroalloy To Develop a Bifunctional Fe–Organic Framework Platform for Enhanced Gas Sorption and Efficient Oxygen Evolution Electrocatalysis. *ACS Appl. Mater. Interfaces* **2020**, *12* (4), 4432–4442.
[36] Reynolds, J. E.; Walsh, K. M.; Li, B.; Kunal, P.; Chen, B.; Humphrey, S. M. Highly Selective Room Temperature Acetylene Sorption by an Unusual Triacetylenic Phosphine MOF. *Chem. Commun.* **2018**, *54* (71), 9937–9940.
[37] Shao, K.; Wang, J.; Pei, J.; Liu, D.; Li, B. Engineering Anion-Pillared Metal–Organic Frameworks for Record Acetylene/Methane Separation. *Zeitschrift anorg allge chemie* **2022**, *648* (18), e202200240.
[38] Chen, F.; Wang, Y.; Bai, D.; He, M.; Gao, X.; He, Y. Selective Adsorption of C_2H_2 and CO_2 from CH_4 in an Isoreticular Series of MOFs Constructed from Unsymmetrical Diisophthalate Linkers and the Effect of Alkoxy Group Functionalization on Gas Adsorption. *J. Mater. Chem. A* **2018**, *6* (8), 3471–3478.
[39] Zhang, Y.; Yang, L.; Wang, L.; Duttwyler, S.; Xing, H. A Microporous Metal-Organic Framework Supramolecularly Assembled from a CuII Dodecaborate Cluster Complex for Selective Gas Separation. *Angew. Chem. Int. Ed.* **2019**, *58* (24), 8145–8150.
[40] Peng, Y.-L.; Pham, T.; Li, P.; Wang, T.; Chen, Y.; Chen, K.-J.; Forrest, K. A.; Space, B.; Cheng, P.; Zaworotko, M. J.; Zhang, Z. Robust Ultramicroporous Metal-Organic Frameworks with Benchmark Affinity for Acetylene. *Angew. Chem. Int. Ed.* **2018**, *57* (34), 10971–10975.
[41] Zhang, Y.; Yang, L.; Wang, L.; Cui, X.; Xing, H. Pillar Iodination in Functional Boron Cage Hybrid Supramolecular Frameworks for High Performance Separation of Light Hydrocarbons. *J. Mater. Chem. A* **2019**, *7* (48), 27560–27566.
[42] Lei, J.; Zhong, Z.-L.; Yuan, W.; Zhang, P.; Wang, Y.; Zhai, Q.-G. Development of Heterometallic Annular Tetranuclear Clusters in Metal–Organic Frameworks for Methane Purification and Storage. *Chem Bio Eng.* **2024**, DOI: 10.1021/cbe.4c00009.
[43] Wang, L.; Ye, Y.; Li, Z.; Lin, Q.; Ouyang, J.; Liu, L.; Zhang, Z.; Xiang, S. Highly Selective Adsorption of C2/C1 Mixtures and Solvent-Dependent Thermochromic Properties in Metal–Organic Frameworks Containing Infinite Copper-Halogen Chains. *Crystal Growth & Design* **2017**, *17* (4), 2081–2089.
[44] Deng, Y.-X.; Yang, G.-P.; Wang, Y.-Y. Pure Separation of Acetylene Based on a Sulfonic Acid and Amino Group Functionalized Zn-MOF. *Chem. Commun.* **2024**, ***60***, 5046-5049.
[45] Wen, H.-M.; Liao, C.; Li, L.; Alsalme, A.; Alothman, Z.; Krishna, R.; Wu, H.; Zhou, W.; Hu, J.; Chen, B. A Metal–Organic Framework with Suitable Pore Size and Dual Functionalities for Highly Efficient Post-Combustion CO_2 Capture. *J. Mater. Chem. A* **2019**, *7* (7), 3128–3134.
[46] Dai, J.; Xie, D.; Liu, Y.; Zhang, Z.; Yang, Y.; Yang, Q.; Ren, Q.; Bao, Z. Supramolecular Metal–Organic Framework for CO_2/CH_4 and CO_2/N_2 Separation. *Ind. Eng. Chem. Res.* **2020**, *59* (16), 7866–7874.

Author Contributions

Q.-W.Y. and T.K. designed the conceptual approach for research; Y.-Y.J carried out the synthesis, experimental tests and computational simulations; G.-H.X contributed to computational simulations; J.-J.L contributed to synthesis and separation test;Z. J. contributed to the in-situ PXRD tests; R. F. contributed to the synthesis· and· experimental tests; Z.-B.B., Z.-G.Z., Q.-L.R., and Q.-W.Y. offered supervision and discussion; Y.-Y.J, T.K. and Q.-W.Y. wrote and revised the manuscript; All authors reviewed and approved the manuscript.
