# Supplementary material for: Ultra-High Purity and Productivity Separation of CO2 and C2H2 from CH4 in Rigid Layered Ultramicroporous Material
Source: ACS Cent Sci. 2024 Sep 20;10(10):1885–93. doi: 10.1021/acscentsci.4c01125 (PMC11503503; doi:10.1021/acscentsci.4c01125)

checkCIF/PLATON report

Structure factors have been supplied for datablock(s) 20120112zju_kt1_0m_a_sq

THIS REPORT IS FOR GUIDANCE ONLY. IF USED AS PART OF A REVIEW PROCEDURE FOR PUBLICATION, IT SHOULD NOT REPLACE THE EXPERTISE OF AN EXPERIENCED CRYSTALLOGRAPHIC REFEREE.

No syntax errors found. CIF dictionary Interpreting this report

Datablock: 20120112zju_kt1_0m_a_sq

Bond precision: C-C = 0.0075 A Wavelength=1.34139

Cell:	a=9.8284(11)	b=19.049(2)	c=8.5345(12)
	alpha=90	beta=90	gamma=90
Temperature:	193 K		

	Calculated	Reported
Volume	1597.8(3)	1597.8(3)
Space group	C m m m	C m m m
Hall group	-C 2 2	-C 2 2
Moiety formula	C20 H16 Cu F6 N4 O4 S2 Ti [+ solvent]	?
Sum formula	C20 H16 Cu F6 N4 O4 S2 Ti [+ solvent]	C20 H16 Cu F6 N4 O4 S2 Ti
Mr	665.91	665.93
Dx,g cm-3	1.384	1.384
Z	2	2
Mu (mm-1)	6.231	6.230
F000	666.0	666.0
F000’	664.00	
h,k,lmax	11,22,10	11,22,10
Nref	832	825
Tmin,Tmax	0.563,0.536	0.522,0.750
Tmin’	0.511	

Correction method= # Reported T Limits: Tmin=0.522 Tmax=0.750
AbsCorr = MULTI-SCAN

Data completeness= 0.992 Theta(max)= 53.046

R(reflections)= 0.0602(661) wR2(reflections)= 0.1655(825)

S = 1.068 Npar= 66

The following ALERTS were generated. Each ALERT has the format

test-name_ALERT_alert-type_alert-level.

Click on the hyperlinks for more details of the test.

Alert level C

```
PLAT234_ALERT_4_C Large Hirshfeld Difference Ti1_2    --F2A_2    .       0.18 Ang.
PLAT234_ALERT_4_C Large Hirshfeld Difference Ti1_2    --F3A_2    .       0.18 Ang.
PLAT242_ALERT_2_C Low    'MainMol' Ueq as Compared to Neighbors of      Ti1_2 Check
PLAT242_ALERT_2_C Low    'MainMol' Ueq as Compared to Neighbors of       C3_1 Check
PLAT250_ALERT_2_C Large U3/U1 Ratio for Average U(i,j) Tensor ....        2.1 Note
PLAT341_ALERT_3_C Low Bond Precision on  C-C Bonds ...............     0.0075 Ang.
PLAT752_ALERT_4_C Angle   Calc       90.00, Rep       90.00 ......  Senseless s.u.
              N1_1    -CU1_1   -F1_2       1.555  1.555  1.555     #   19 Check
PLAT911_ALERT_3_C Missing FCF Refl Between Thmin & STh/L=    0.596          8 Report
```

Alert level G

```
ABSMU01_ALERT_1_G  Calculation of _exptl_absorpt_correction_mu
                not performed for this radiation type.
PLAT003_ALERT_2_G Number of Uiso or Uij Restrained non-H Atoms ...         11 Report
PLAT004_ALERT_5_G Polymeric Structure Found with Maximum Dimension          2 Info
PLAT083_ALERT_2_G SHELXL Second Parameter in WGHT  Unusually Large       7.94 Why ?
PLAT178_ALERT_4_G The CIF-Embedded .res File Contains SIMU Records          1 Report
PLAT300_ALERT_4_G Atom Site Occupancy of F2_2       Constrained at        0.6 Check
PLAT300_ALERT_4_G Atom Site Occupancy of F2A_2      Constrained at        0.4 Check
PLAT300_ALERT_4_G Atom Site Occupancy of F3A_2      Constrained at        0.4 Check
PLAT301_ALERT_3_G Main Residue  Disorder ..............(Resd  1  )        20% Note
PLAT605_ALERT_4_G Largest Solvent Accessible VOID in the Structure        497 A**3
PLAT720_ALERT_4_G Number of Unusual/Non-Standard Labels ..........         14 Note
PLAT794_ALERT_5_G Tentative Bond Valency for Cu1_1     (II)      .       2.07 Info
PLAT860_ALERT_3_G Number of Least-Squares Restraints .............         66 Note
PLAT869_ALERT_4_G ALERTS Related to the Use of SQUEEZE Suppressed           ! Info
PLAT909_ALERT_3_G Percentage of I>2sig(I) Data at Theta(Max) Still        50% Note
PLAT933_ALERT_2_G Number of OMIT Records in Embedded .res File ...          1 Note
PLAT978_ALERT_2_G Number C-C Bonds with Positive Residual Density.          0 Info
```

---

0 **ALERT level A** = Most likely a serious problem - resolve or explain
0 **ALERT level B** = A potentially serious problem, consider carefully
8 **ALERT level C** = Check. Ensure it is not caused by an omission or oversight
17 **ALERT level G** = General information/check it is not something unexpected

```
 1 ALERT type 1 CIF construction/syntax error, inconsistent or missing data
 7 ALERT type 2 Indicator that the structure model may be wrong or deficient
 5 ALERT type 3 Indicator that the structure quality may be low
10 ALERT type 4 Improvement, methodology, query or suggestion
 2 ALERT type 5 Informative message, check
```

It is advisable to attempt to resolve as many as possible of the alerts in all categories. Often the minor alerts point to easily fixed oversights, errors and omissions in your CIF or refinement strategy, so attention to these fine details can be worthwhile. In order to resolve some of the more serious problems it may be necessary to carry out additional measurements or structure refinements. However, the purpose of your study may justify the reported deviations and the more serious of these should normally be commented upon in the discussion or experimental section of a paper or in the "special_details" fields of the CIF. checkCIF was carefully designed to identify outliers and unusual parameters, but every test has its limitations and alerts that are not important in a particular case may appear. Conversely, the absence of alerts does not guarantee there are no aspects of the results needing attention. It is up to the individual to critically assess their own results and, if necessary, seek expert advice.

Publication of your CIF in IUCr journals

A basic structural check has been run on your CIF. These basic checks will be run on all CIFs submitted for publication in IUCr journals (*Acta Crystallographica*, *Journal of Applied Crystallography*, *Journal of Synchrotron Radiation*); however, if you intend to submit to *Acta Crystallographica Section C* or *E* or *IUCrData*, you should make sure that full publication checks are run on the final version of your CIF prior to submission.

Publication of your CIF in other journals

Please refer to the *Notes for Authors* of the relevant journal for any special instructions relating to CIF submission.

PLATON version of 05/12/2020; check.def file version of 05/12/2020

Datablock 20120112zju_kt1_0m_a_sq - ellipsoid plot

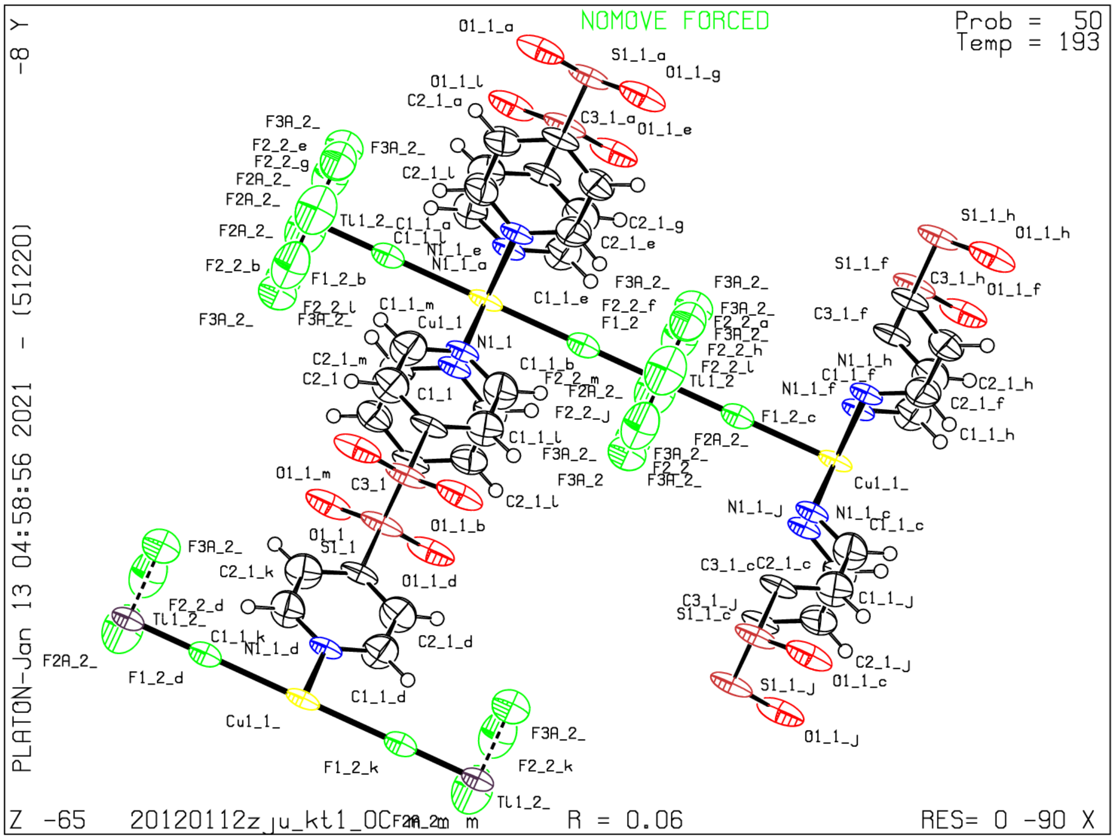

Supplement: Supplementary file 4 — oc4c01125_si_004.pdf [file oc4c01125_si_004.pdf]
