# Supplementary material for: Ultra-High Purity and Productivity Separation of CO2 and C2H2 from CH4 in Rigid Layered Ultramicroporous Material
Source: ACS Cent Sci. 2024 Sep 20;10(10):1885–93. doi: 10.1021/acscentsci.4c01125 (PMC11503503; doi:10.1021/acscentsci.4c01125)

checkCIF/PLATON report

Structure factors have been supplied for datablock(s) 240119lu_zju_kt_1_0m

THIS REPORT IS FOR GUIDANCE ONLY. IF USED AS PART OF A REVIEW PROCEDURE FOR PUBLICATION, IT SHOULD NOT REPLACE THE EXPERTISE OF AN EXPERIENCED CRYSTALLOGRAPHIC REFEREE.

No syntax errors found.   CIF dictionary   Interpreting this report

Datablock: 240119lu_zju_kt_1_0m

Bond precision:	C-C = 0.0063 A		Wavelength=0.71073
Cell:	a=9.8977(6)	b=18.8759(13)	c=8.5038(6)
	alpha=90	beta=90	gamma=90
Temperature:	193 K		

	Calculated	Reported
Volume	1588.75(18)	1588.75(18)
Space group	C m m m	C m m m
Hall group	-C 2 2	-C 2 2
Moiety formula	4(C5 H4 Cu0.25 F1.50 N O S0.50 Ti0.25), C H, 3(C H)	C20 H16 Cu F6 N4 O4 S2 Ti, 3(C2 H2)
Sum formula	C26 H22 Cu F6 N4 O4 S2 Ti	C26 H22 Cu F6 N4 O4 S2 Ti
Mr	744.02	744.03
Dx,g cm-3	1.555	1.555
Z	2	2
Mu (mm-1)	1.125	1.125
F000	750.0	750.0
F000'	752.08	
h,k,lmax	12,24,10	12,24,10
Nref	1038	1034
Tmin,Tmax	0.845,0.874	0.222,0.261
Tmin'	0.845	

Correction method= # Reported T Limits: Tmin=0.222 Tmax=0.261
AbsCorr = MULTI-SCAN

Data completeness= 0.996   Theta(max)= 27.226

R(reflections)= 0.0409(928)   wR2(reflections)= 0.1140(1034)

S = 1.165   Npar= 79

The following ALERTS were generated. Each ALERT has the format
test-name_ALERT_alert-type_alert-level.
Click on the hyperlinks for more details of the test.

Alert level C

```
PLAT042_ALERT_1_C Calc. and Reported MoietyFormula Strings  Differ     Please Check
              Calc: 4(C5 H4 Cu0.25 F1.50 N O S0.50 Ti0.25), C H, 3(C H)
              Rep.: C20 H16 Cu F6 N4 O4 S2 Ti, 3(C2 H2)
PLAT213_ALERT_2_C Atom F2              has ADP max/min Ratio .....        3.2 prolat
PLAT220_ALERT_2_C NonSolvent   Resd 1  F   Ueq(max)/Ueq(min) Range        3.8 Ratio
PLAT242_ALERT_2_C Low    'MainMol' Ueq as Compared to Neighbors of        Ti1 Check
PLAT245_ALERT_2_C U(iso) H5        Smaller than U(eq) C5        by      0.028 Ang**2
PLAT245_ALERT_2_C U(iso) H4        Smaller than U(eq) C4        by      0.021 Ang**2
PLAT260_ALERT_2_C Large Average Ueq of Residue Including        C6      0.109 Check
PLAT341_ALERT_3_C Low Bond Precision on  C-C Bonds ..............     0.00633 Ang.
PLAT906_ALERT_3_C Large K Value in the Analysis of Variance ......      2.122 Check
PLAT911_ALERT_3_C Missing FCF Refl Between Thmin & STh/L=    0.600          4 Report
                1  1  0,   2  4  0,   0  0  1,   0  0  2,
PLAT918_ALERT_3_C Reflection(s) with I(obs) much Smaller I(calc) .          1 Check
PLAT975_ALERT_2_C Check Calcd Resid. Dens.  0.89Ang From C6      .       0.82 eA-3
PLAT975_ALERT_2_C Check Calcd Resid. Dens.  0.94Ang From C6      .       0.41 eA-3
```

Alert level G

```
PLAT003_ALERT_2_G Number of Uiso or Uij Restrained non-H Atoms ...          5 Report
PLAT004_ALERT_5_G Polymeric Structure Found with Maximum Dimension          2 Info
PLAT083_ALERT_2_G SHELXL Second Parameter in WGHT  Unusually Large       5.38 Why ?
PLAT169_ALERT_4_G The CIF-Embedded .res File Contains AFIX 1 Recds          3 Report
PLAT186_ALERT_4_G The CIF-Embedded .res File Contains ISOR Records          2 Report
PLAT300_ALERT_4_G Atom Site Occupancy of F2          Constrained at       0.8 Check
PLAT300_ALERT_4_G Atom Site Occupancy of F2A         Constrained at      0.25 Check
PLAT300_ALERT_4_G Atom Site Occupancy of F2B         Constrained at      0.15 Check
PLAT300_ALERT_4_G Atom Site Occupancy of C6          Constrained at       0.5 Check
PLAT300_ALERT_4_G Atom Site Occupancy of H6B         Constrained at     0.125 Check
PLAT300_ALERT_4_G Atom Site Occupancy of C4          Constrained at       0.5 Check
PLAT300_ALERT_4_G Atom Site Occupancy of H4          Constrained at       0.5 Check
PLAT301_ALERT_3_G Main Residue  Disorder .............(Resd    1)         18% Note
PLAT302_ALERT_4_G Anion/Solvent/Minor-Residue Disorder (Resd    2)       100% Note
PLAT302_ALERT_4_G Anion/Solvent/Minor-Residue Disorder (Resd    4)       100% Note
PLAT315_ALERT_2_G Singly Bonded Carbon Detected (H-atoms Missing).         C4 Check
PLAT432_ALERT_2_G Short Inter X...Y Contact  F2B       ..C6       .      2.73 Ang.
                                                     x,y,z  =      1_555 Check
PLAT432_ALERT_2_G Short Inter X...Y Contact  C4        ..C4       .      1.26 Ang.
                                                  x,1-y,-z  =      4_565 Check
PLAT432_ALERT_2_G Short Inter X...Y Contact  C6        ..C6       .      0.99 Ang.
                                                 2-x,1-y,z  =      2_765 Check
PLAT789_ALERT_4_G Atoms with Negative _atom_site_disorder_group  #          1 Check
PLAT794_ALERT_5_G Tentative Bond Valency for Cu1       (II)       .      2.08 Info
PLAT822_ALERT_4_G CIF-embedded .res Contains Negative PART Numbers          1 Check
PLAT860_ALERT_3_G Number of Least-Squares Restraints ............          30 Note
PLAT913_ALERT_3_G Missing # of Very Strong Reflections in FCF ....          2 Note
                2  4  0,   0  0  2,
PLAT969_ALERT_5_G The 'Henn et al.' R-Factor-gap value ...........       3.49 Note
              Predicted wR2: Based on SigI**2  3.27 or SHELX Weight 10.18
PLAT978_ALERT_2_G Number C-C Bonds with Positive Residual Density.          3 Info
```

```
 0 ALERT level A = Most likely a serious problem - resolve or explain
 0 ALERT level B = A potentially serious problem, consider carefully
13 ALERT level C = Check. Ensure it is not caused by an omission or oversight
26 ALERT level G = General information/check it is not something unexpected

 1 ALERT type 1 CIF construction/syntax error, inconsistent or missing data
15 ALERT type 2 Indicator that the structure model may be wrong or deficient
 7 ALERT type 3 Indicator that the structure quality may be low
13 ALERT type 4 Improvement, methodology, query or suggestion
 3 ALERT type 5 Informative message, check
```

It is advisable to attempt to resolve as many as possible of the alerts in all categories. Often the minor alerts point to easily fixed oversights, errors and omissions in your CIF or refinement strategy, so attention to these fine details can be worthwhile. In order to resolve some of the more serious problems it may be necessary to carry out additional measurements or structure refinements. However, the purpose of your study may justify the reported deviations and the more serious of these should normally be commented upon in the discussion or experimental section of a paper or in the "special_details" fields of the CIF. checkCIF was carefully designed to identify outliers and unusual parameters, but every test has its limitations and alerts that are not important in a particular case may appear. Conversely, the absence of alerts does not guarantee there are no aspects of the results needing attention. It is up to the individual to critically assess their own results and, if necessary, seek expert advice.

Publication of your CIF in IUCr journals

A basic structural check has been run on your CIF. These basic checks will be run on all CIFs submitted for publication in IUCr journals (*Acta Crystallographica*, *Journal of Applied Crystallography*, *Journal of Synchrotron Radiation*); however, if you intend to submit to *Acta Crystallographica Section C* or *E* or *IUCrData*, you should make sure that full publication checks are run on the final version of your CIF prior to submission.

Publication of your CIF in other journals

Please refer to the *Notes for Authors* of the relevant journal for any special instructions relating to CIF submission.

PLATON version of 06/01/2024; check.def file version of 05/01/2024

Datablock 240119lu_zju_kt_1_0m - ellipsoid plot

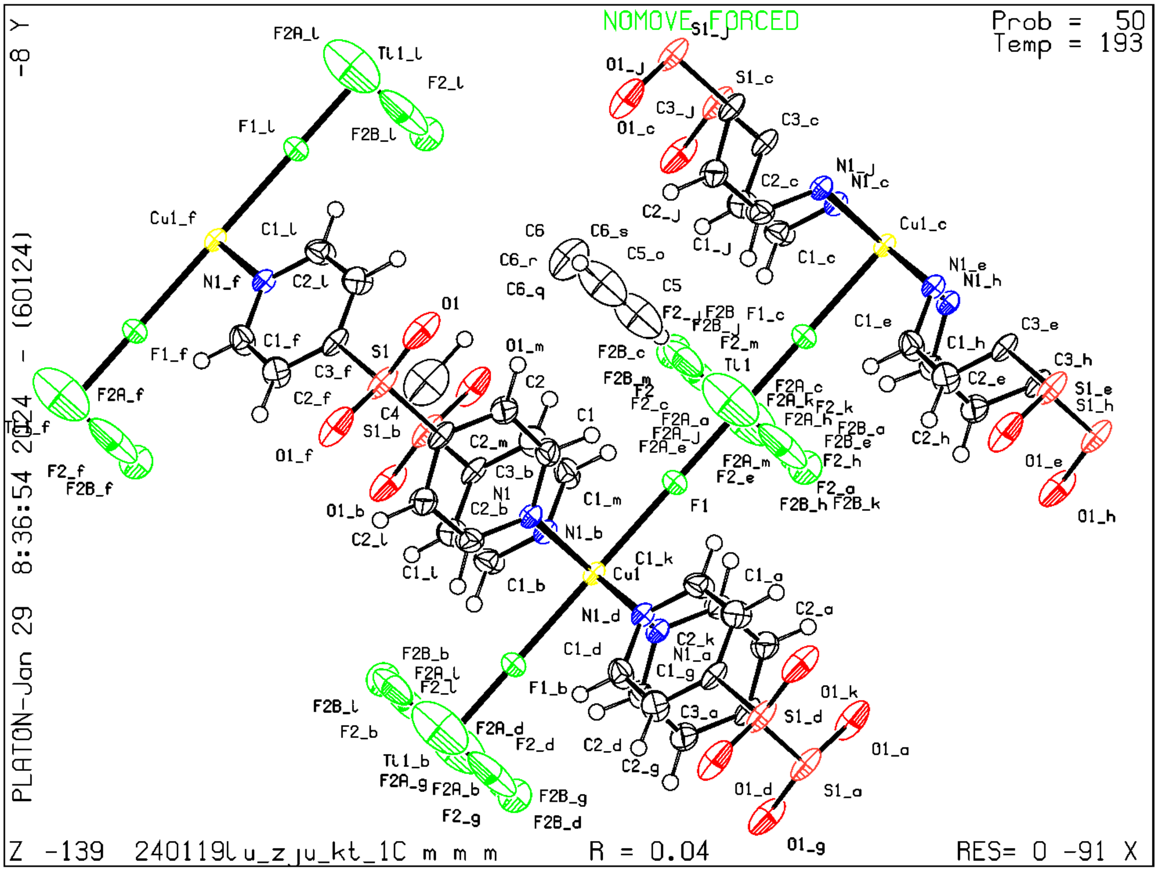

Supplement: Supplementary file 5 — oc4c01125_si_005.pdf [file oc4c01125_si_005.pdf]
