# Supplementary material for: Ultra-High Purity and Productivity Separation of CO2 and C2H2 from CH4 in Rigid Layered Ultramicroporous Material
Source: ACS Cent Sci. 2024 Sep 20;10(10):1885–93. doi: 10.1021/acscentsci.4c01125 (PMC11503503; doi:10.1021/acscentsci.4c01125)

checkCIF/PLATON report

Structure factors have been supplied for datablock(s) 240119lu_zju_kt_2_0m

THIS REPORT IS FOR GUIDANCE ONLY. IF USED AS PART OF A REVIEW PROCEDURE FOR PUBLICATION, IT SHOULD NOT REPLACE THE EXPERTISE OF AN EXPERIENCED CRYSTALLOGRAPHIC REFEREE.

No syntax errors found.   CIF dictionary   Interpreting this report

Datablock: 240119lu_zju_kt_2_0m

Bond precision:	C-C = 0.0065 A	Wavelength=0.71073	
Cell:	a=9.7457(13)	b=19.293(3)	c=8.2873(11)
	alpha=90	beta=90	gamma=90
Temperature:	193 K		

	Calculated	Reported
Volume	1558.2(4)	1558.2(4)
Space group	C m m m	C m m m
Hall group	-C 2 2	-C 2 2
Moiety formula	4(C5 H4 Cu0.25 F1.50 N O S0.50 Ti0.25), C0.10 O0.20, C0.10 O0.2	C20 H16 Cu F6 N4 O4 S2 Ti, 0.6(C2 O4)
Sum formula	C21.20 H16 Cu F6 N4 O6.40 S2 Ti	C21.20 H16 Cu F6 N4 O6.40 S2 Ti
Mr	718.72	718.74
Dx,g cm-3	1.532	1.532
Z	2	2
Mu (mm-1)	1.149	1.149
F000	718.8	719.0
F000'	720.91	
h,k,lmax	12,24,10	12,24,10
Nref	1043	1043
Tmin,Tmax	0.851,0.891	0.205,0.262
Tmin'	0.851	

Correction method= # Reported T Limits: Tmin=0.205 Tmax=0.262
AbsCorr = MULTI-SCAN

Data completeness= 1.000   Theta(max)= 27.493

R(reflections)= 0.0623(883) wR2(reflections)= 0.1750(1043)

S = 1.187 Npar= 87

The following ALERTS were generated. Each ALERT has the format
test-name_ALERT_alert-type_alert-level.
Click on the hyperlinks for more details of the test.

Alert level C

PLAT042_ALERT_1_C Calc. and Reported MoietyFormula Strings Differ Please Check
Calc: 4(C5 H4 Cu0.25 F1.50 N O S0.50 Ti0.25), C0.10 O0.20, C0.10 O
Rep.: C20 H16 Cu F6 N4 O4 S2 Ti, 0.6(C2 O4)
PLAT077_ALERT_4_C Unitcell Contains Non-integer Number of Atoms .. Please Check
PLAT094_ALERT_2_C Ratio of Maximum / Minimum Residual Density .... 2.05 Report
PLAT242_ALERT_2_C Low 'MainMol' Ueq as Compared to Neighbors of Ti1 Check
PLAT341_ALERT_3_C Low Bond Precision on C-C Bonds .... 0.0065 Ang.
PLAT752_ALERT_4_C Angle Calc 90.00, Rep 90.00 ...... Senseless s.u.
N1 -CU1 -F1 1_555 1_555 1_555 # 2 Check
PLAT906_ALERT_3_C Large K Value in the Analysis of Variance ...... 6.101 Check

---

Alert level G

PLAT002_ALERT_2_G Number of Distance or Angle Restraints on AtSite 7 Note
PLAT003_ALERT_2_G Number of Uiso or Uij Restrained non-H Atoms ... 7 Report
PLAT004_ALERT_5_G Polymeric Structure Found with Maximum Dimension 2 Info
PLAT068_ALERT_1_G Reported F000 Differs from Calcd (or Missing)... Please Check
PLAT083_ALERT_2_G SHELXL Second Parameter in WGHT Unusually Large 11.00 Why ?
PLAT169_ALERT_4_G The CIF-Embedded .res File Contains AFIX 1 Recds 2 Report
PLAT176_ALERT_4_G The CIF-Embedded .res File Contains SADI Records 3 Report
PLAT186_ALERT_4_G The CIF-Embedded .res File Contains ISOR Records 3 Report
PLAT300_ALERT_4_G Atom Site Occupancy of F2 Constrained at 0.5 Check
PLAT300_ALERT_4_G Atom Site Occupancy of F2A Constrained at 0.5 Check
PLAT300_ALERT_4_G Atom Site Occupancy of O3 Constrained at 0.1 Check
PLAT300_ALERT_4_G Atom Site Occupancy of C5A Constrained at 0.1 Check
PLAT300_ALERT_4_G Atom Site Occupancy of O3A Constrained at 0.1 Check
PLAT300_ALERT_4_G Atom Site Occupancy of C5 Constrained at 0.1 Check
PLAT300_ALERT_4_G Atom Site Occupancy of O2 Constrained at 0.25 Check
PLAT300_ALERT_4_G Atom Site Occupancy of O2A Constrained at 0.25 Check
PLAT300_ALERT_4_G Atom Site Occupancy of C4 Constrained at 0.5 Check
PLAT301_ALERT_3_G Main Residue Disorder ....(Resd 1) 15% Note
PLAT302_ALERT_4_G Anion/Solvent/Minor-Residue Disorder (Resd 2) 100% Note
PLAT302_ALERT_4_G Anion/Solvent/Minor-Residue Disorder (Resd 3) 100% Note
PLAT302_ALERT_4_G Anion/Solvent/Minor-Residue Disorder (Resd 4) 100% Note
PLAT432_ALERT_2_G Short Inter X...Y Contact F2A ..C4 . 2.76 Ang.
x,y,z = 1_555 Check
PLAT432_ALERT_2_G Short Inter X...Y Contact O1 ..C4 . 2.90 Ang.
3/2-x,1/2-y,-1+z = 6_654 Check
PLAT794_ALERT_5_G Tentative Bond Valency for Cu1 (II) . 2.11 Info
PLAT811_ALERT_5_G No ADDSYM Analysis: Too Many Excluded Atoms .... ! Info
PLAT860_ALERT_3_G Number of Least-Squares Restraints .... 44 Note
PLAT969_ALERT_5_G The 'Henn et al.' R-Factor-gap value .... 4.35 Note
Predicted wR2: Based on SigI**2 4.03 or SHELX Weight 15.40
PLAT978_ALERT_2_G Number C-C Bonds with Positive Residual Density. 1 Info

```
 0 ALERT level A = Most likely a serious problem - resolve or explain
 0 ALERT level B = A potentially serious problem, consider carefully
 7 ALERT level C = Check. Ensure it is not caused by an omission or oversight
28 ALERT level G = General information/check it is not something unexpected

 2 ALERT type 1 CIF construction/syntax error, inconsistent or missing data
 8 ALERT type 2 Indicator that the structure model may be wrong or deficient
 4 ALERT type 3 Indicator that the structure quality may be low
17 ALERT type 4 Improvement, methodology, query or suggestion
 4 ALERT type 5 Informative message, check
```

It is advisable to attempt to resolve as many as possible of the alerts in all categories. Often the minor alerts point to easily fixed oversights, errors and omissions in your CIF or refinement strategy, so attention to these fine details can be worthwhile. In order to resolve some of the more serious problems it may be necessary to carry out additional measurements or structure refinements. However, the purpose of your study may justify the reported deviations and the more serious of these should normally be commented upon in the discussion or experimental section of a paper or in the "special_details" fields of the CIF. checkCIF was carefully designed to identify outliers and unusual parameters, but every test has its limitations and alerts that are not important in a particular case may appear. Conversely, the absence of alerts does not guarantee there are no aspects of the results needing attention. It is up to the individual to critically assess their own results and, if necessary, seek expert advice.

Publication of your CIF in IUCr journals

A basic structural check has been run on your CIF. These basic checks will be run on all CIFs submitted for publication in IUCr journals (*Acta Crystallographica*, *Journal of Applied Crystallography*, *Journal of Synchrotron Radiation*); however, if you intend to submit to *Acta Crystallographica Section C* or *E* or *IUCrData*, you should make sure that full publication checks are run on the final version of your CIF prior to submission.

Publication of your CIF in other journals

Please refer to the *Notes for Authors* of the relevant journal for any special instructions relating to CIF submission.

PLATON version of 06/01/2024; check.def file version of 05/01/2024

Datablock 240119lu_zju_kt_2_0m - ellipsoid plot

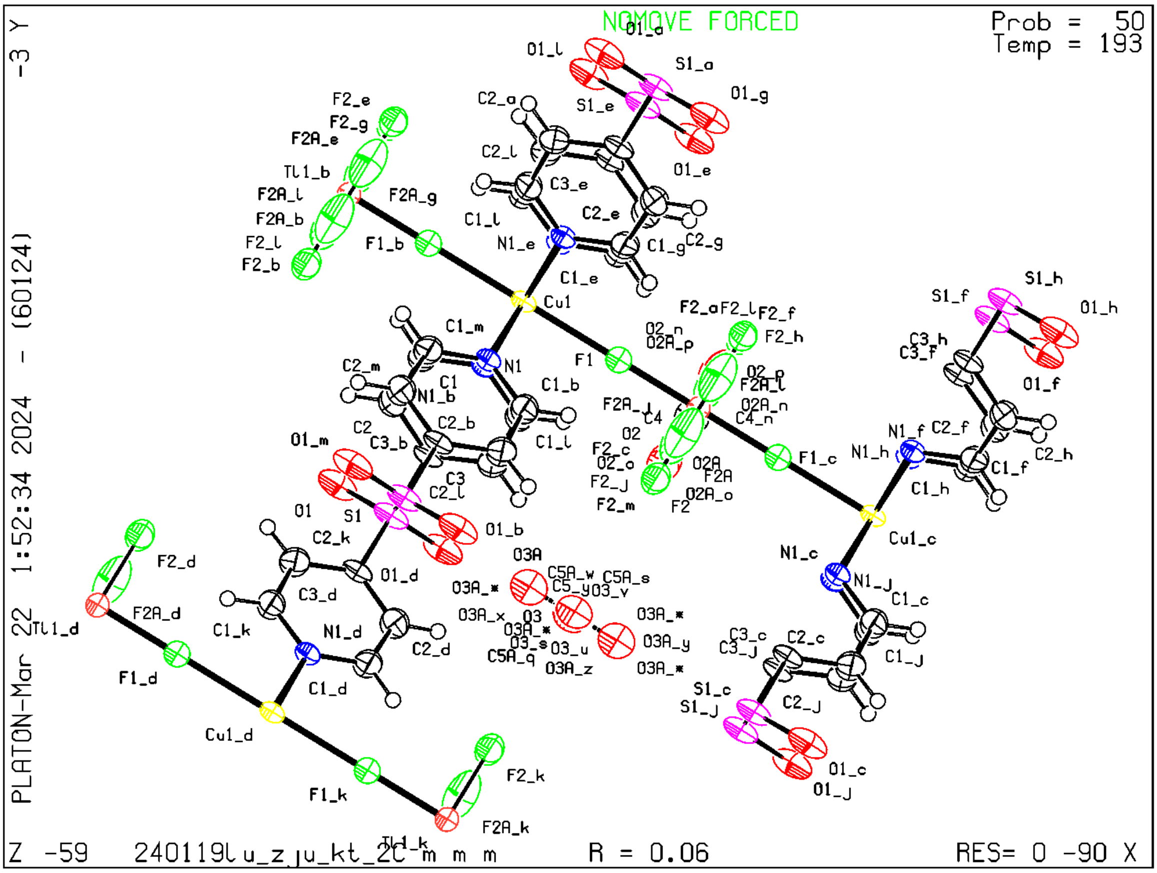

Supplement: Supplementary file 6 — oc4c01125_si_006.pdf [file oc4c01125_si_006.pdf]
