# Supplementary material for: Ultra-High Purity and Productivity Separation of CO2 and C2H2 from CH4 in Rigid Layered Ultramicroporous Material
Source: ACS Cent Sci. 2024 Sep 20;10(10):1885–93. doi: 10.1021/acscentsci.4c01125 (PMC11503503; doi:10.1021/acscentsci.4c01125)
Supplement: Supplementary file 8 — oc4c01125_si_008.pdf [file oc4c01125_si_008.pdf]

oc-2024-01125e.R1

Name: Peer Review Information for "Ultra-High Purity and Productivity Separation of CO_2 and C_2H_2 from CH_4 in Rigid Layered Ultramicroporous Material"

First Round of Reviewer Comments

Reviewer: 1

Comments to the Author

This is a very nice work, demonstrating another example of MOFs for these important gas separations. The comparision of the IAST selectivities and gas adsorption uptakes at 0.5 bar and 298 K might be not reasonable, because CO_2/CH_4 and C_2H_2/CH_4 separations are not carried out at 50%:50% gas ratio, so need to be recalcuated based on the pratical separation conditions (for example, flue gas separation is about 15% CO_2...), overclaiming the results are not good.

Reviewer: 2

Comments to the Author

The authors reported in this manuscript a robust 2D layered ultramicroporous framework (ZUL-100) featuring synergistic ligand/anion binding mode and multiple diffusion pathways for efficient CO_2/CH_4 and C_2H_2/CH_4 separation. This study is very systematic, comprehensive, and insightful warranting publication in ACS Central Science. This reviewer only has some minor comments as listed below.

1. It is suggested the authors conduct breakthrough experiments under certain humidities.

2. It is suggested that the authors measure the kinetic profiles for the adsorption of CO_2, C_2H_2, and CH_4 by ZUL-100.

3. To monitor the possible structural transformations of ZUL-100 when adsorbing gases, it is suggested that in-situ PXRD patterns of ZUL-100 loaded with CO_2, C_2H_2, CH_4 at different partial pressures (e.g. 0.25 bar, 0.5bar, 1bar) be collected.

Author's Response to Peer Review Comments:

38 Zheda Road
Hangzhou 310027, China

ZHEJIANG UNIVERSITY

Fax: +86-571-87951225

College of Chemical and Biological Engineering

Email: yangqw@zju.edu.cn

Dear Editor Sept 5, 2024

Senior Editor, ACS Central Science

Thank you very much for your letter date Aug 20, 2024 and forwarding the referees' comments regarding our manuscript entitled "Ultra-High Purity and Productivity Separation of CO_2 and C_2H_2 from CH_4 in Rigid Layered Ultramicroporous Material" (oc-2024-01125e). We are pleased that the two referees have offered such constructive comments on our results and given us very important suggestions for improving the scientific findings. According to the referees' comments, we have made modifications and corrections. In addition, extra data have been supplemented to make our results convincing. We believe that our revisions to the manuscript and SI, which are highlighted in yellow, fully address the comments of all referees. Point-by-point changes and/or replies to the comments of referees are detailed as follows:

Comments from Reviewer 1:

This is a very nice work, demonstrating another example of MOFs for these important gas separations.

Response: Thanks for the reviewer's positive comment.

Comment. The comparisons of the IAST selectivities and gas adsorption uptakes at 0.5 bar and 298 K might be not reasonable, because CO_2/CH_4 and C_2H_2/CH_4 separations are not carried out at 50%:50% gas ratio, so need to be recalculated based on the practical separation conditions (for example, flue gas separation is about 15% CO_2), overclaiming the results are not good.

Response:

Thanks for the reviewer's constructive comment. For the CO_2/CH_4 system, the CO_2 content of the natural gas (NG) feedstock varies significantly depending on its source. In some instances, the CO_2 content is lower than that of CH_4, while in other cases, it exceeds CH_4 levels. Therefore, we chose a 50/50 ratio in the previous manuscript because a 50/50 ratio is representative of an average condition. In addition, this ratio was commonly used in the literature on this field for separation studies and allowed for a more comprehensive comparison of separation performance under the same conditions. Nevertheless, as pointed out by the reviewer, a 15% CO_2 concentration is also a critical separation scenario. Accordingly, in the revised version, we have included the CO_2 uptake

data at 15 kPa (corresponding to a 15% CO_2 concentration at atmospheric pressure) for ZUL-100 and various literature adsorbents. At the same time, we recalculated the IAST selectivity for the 15/85 CO_2/CH_4 mixture on these materials. The results showed that ZUL-100 achieved superior CO_2 uptake (64.5 cm^3/g at 15 kPa, Figure S13) and CO_2/CH_4 selectivity (15/85, 7555, Figure S13). The IAST predicted uptakes of CO_2 and CH_4 on ZUL-100 for the adsorption of CO_2/CH_4 15/85 (*v/v*) mixtures at 298 K were close to their static uptakes, while the CH_4 uptake for the CO_2/CH_4 mixture is only 0.002 mmol/g (Figure S10) due to the strong competitive adsorption effects of CO_2 and CH_4. We also performed breakthrough experiments using the same 15/85 CO_2/CH_4 mixture on ZUL-100 as well as two benchmark MOFs (SIFSIX-14-Cu-i and SIFSIX-3-Cu) for comparison. The highpurity CH_4 productivity (14.6 mmol/g, Figure S36) was still significantly higher than that of SIFSIX-14-Cu-i and SIFSIX-3-Cu, at reduced CO_2 concentration. In addition, ZUL-100 exhibited a 70% reduction in desorption time compared to SIFSIX-3-Cu (Figure S38).

For the C_2H_2/ CH_4 system, it is noted that in the C_2H_2 crude gas derived from NG partial oxidation, the concentration of C_2H_2 (7%-10%) closely matches that of CH_4 (~5%) (Chemical Engineering Journal, **2021**, *426*, 130871; Acetylene. Ullmann's Encyclopedia of Industrial Chemistry **2011**, *1*, 277−323), but both were present in relatively dilute concentrations. Therefore, we also included the C_2H_2 uptake data at 10 kPa, added the IAST uptakes (Figure S12) of C_2H_2 and CH_4 on ZUL-100 for the adsorption of C_2H_2/CH_4 10/5 (*v/v*) mixtures at 298 K, recalculated and compared the IAST selectivity for C_2H_2/CH_4 under a 10/5 ratio for ZUL-100 and literature adsorbents. Excitingly, the results also showed that ZUL-100 still exhibited superior C_2H_2 uptake (96.5 cm^3/g at 10 kPa, Figure S14) and C_2H_2/CH_4 selectivity at low C_2H_2 concentrations compared to those of the literature adsorbents.

Modification:

(1) Main text, page 4, line 32 in the left column, the sentence "At the same time, the IAST selectivities for the CO_2/CH_4 and C_2H_2/CH_4 mixtures at different ratios (relevant to different industrial scenarios) remained excellent on ZUL-100 (Figures S13 and S14)" was added.

(2) Main text, page 6, line 8 in the left column, the sentence "The dynamic CO_2/CH_4 separation performance of these three materials was also evaluated with a 15/85 CO_2/CH_4 ratio, and the results showed that ZUL-100 still exhibited much higher CO_2 uptake (2.68 mmol/g) and CH_4 productivity (14.6 mmol/g) than SIFSIX-14-Cu-i (0.64 mmol/g/3.64 mmol/g) and SIFSIX-3-Cu (2.02 mmol/g/10.1 mmol/g) despite the lower CO_2 concentration." was added.

(3) Figures S10, S12-14 and Tables S11-12 have been added into the Supporting Information:

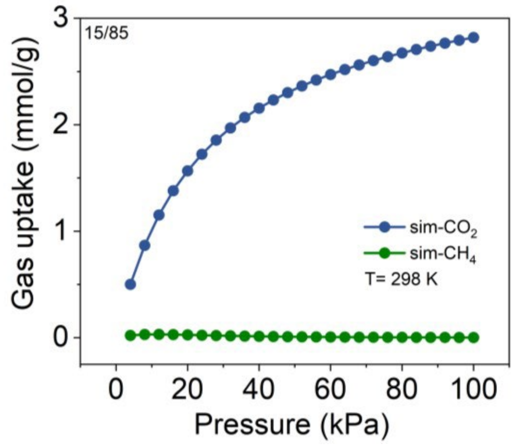

Figure S10. Competitive adsorption isotherms of CO_2 and CH_4 for CO_2/CH_4 15/85 (*v/v*) mixture on ZUL-100 at 298 K.

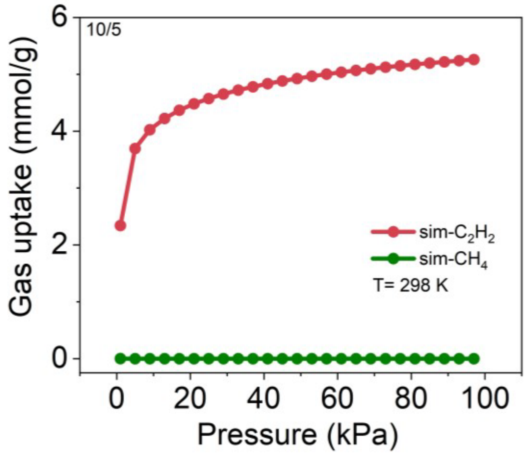

Figure S12. Competitive adsorption isotherms of C_2H_2 and CH_4 for C_2H_2/CH_4 10/5 (*v/v*) mixture on ZUL-100 at 298 K.

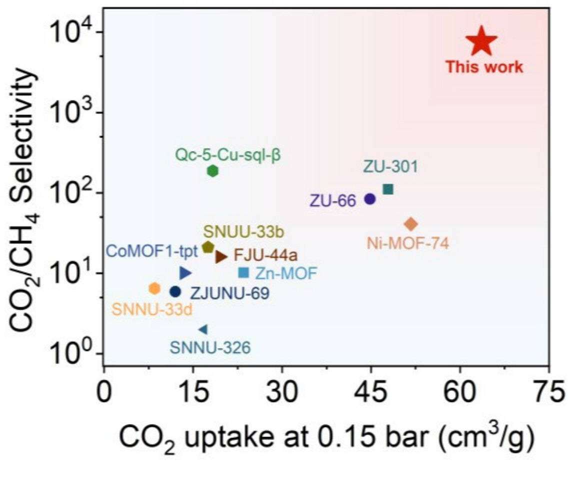

Figure S13. Comparison of the IAST selectivities and CO_2 adsorption uptakes at 0.15 bar and 298 K of ZUL-100 with representative porous materials.

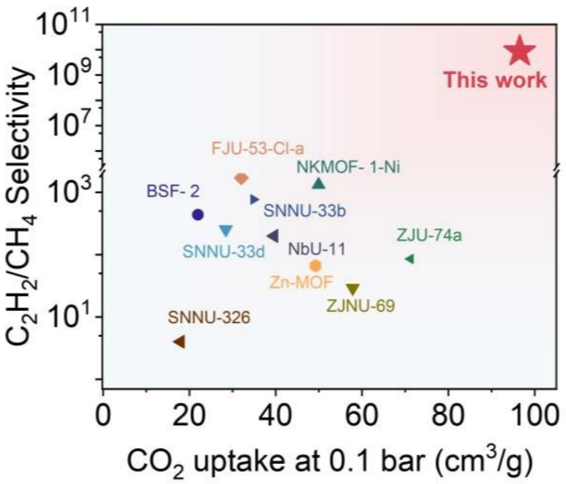

Figure S14. Comparison of the IAST selectivities and C_2H_2 adsorption uptakes at 0.1 bar and 298 K of ZUL-100 with representative porous materials.

Table S11. Summary of the separation performance for CO_2 and CH_4 in representative materials.

MOFs	0.15 bar CO_2 uptake (cm^3/g)	CO_2/CH_4 selectivity (15/85) at 298 K and 1 bar	Ref
**ZUL-100**	**63.6**	**7555**	**This work**
Qc-5-Cu-sql-β	18.3	188	[21]
ZU-301	47.848	111	[22]
ZU-66	44.8	84	[27]
Ni-MOF-74	51.7	41	[11]
FJU-44a	19.5	16	[13]
CoMOF1-tpt	13.44	10.1	[14]
Zn-MOF	23.5	10.2	[44]
SNNU-326	16.8	2	[42]
SNNU-33d	8.5	6.5	[26]
SNUU-33b	17.5	21	[26]
ZJUNU-69	12	5.9	[38]

Table S12. Summary of the separation performance for C_2H_2 and CH_4 in representative materials.

MOFs	0.1 bar C_2H_2 uptake (cm^3/g)	C_2H_2/CH_4 selectivity (10/5, *v*/*v*) at 298 K and 1 bar	Ref
**ZUL-100**	**96.5**	**8.5×10^9**	**This work**
ZJU-74a	71.2	85	[28]
NKMOF- 1-Ni	49.9	1315	[40]

BSF- 2	21.952	434	[41]
FJU-53-Cl-a	32	1705	[43]
SNNU-326	17.92	4	[42]
SNNU-33b	34.8	767	[10]
SNNU-33d	28.4	253	[26]
CuDTTA	34.5	6476	[26]
Zn-MOF	49.2	66	[44]
ZJNU-69	57.8	29	[38]

(4) Supporting Information, page 4, section 'Breakthrough tests': the sentence "Moreover, 2 mL/min for CO_2/CH_4 50/50 (*v/v*) mixture containing 1000 ppm H_2O and 4 mL/min for CO_2/CH_4 15/85 (*v/v*) mixture." has been added.

(5) Figures S36 and S38 have been supplemented into the Supporting Information:

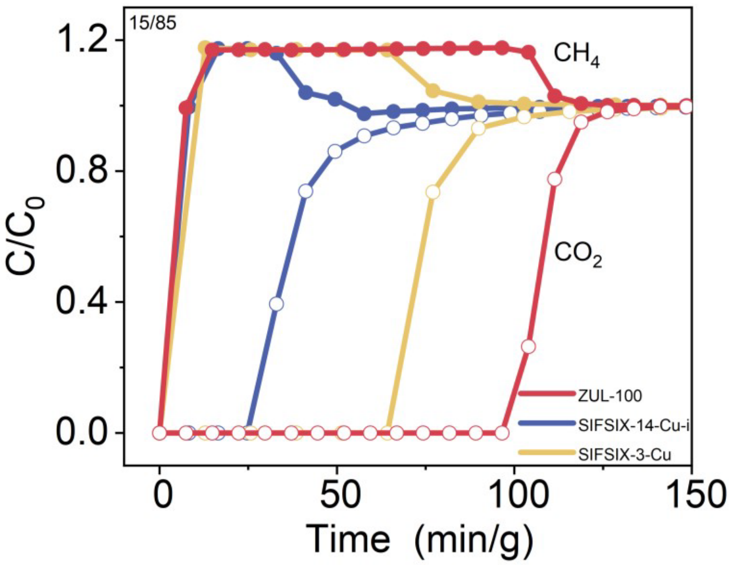

Figure S36. Breakthrough curves of CO_2/CH_4 (15/85, *v/v*) mixture on ZUL-100 (red), SIFSIX-3-Cu (yellow) and SIFSIX-14Cu-i. (blue) at 298 K with a flow rate of 4 mL/min.

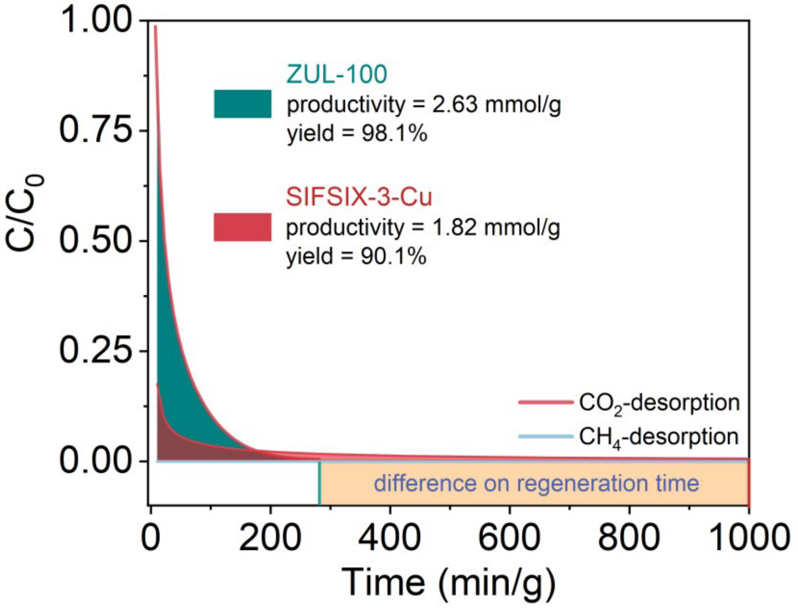

Figure S38. Desorption curves for saturated ZUL-100 and SIFSIX-3-Cu column of CO_2/CH_4 (15/85, *v*/*v*) mixture with He purge at 313 K with a flow rate of 10 mL/min.

Comments from Reviewer 2:

The authors reported in this manuscript a robust 2D layered ultramicroporous framework (ZUL100) featuring synergistic ligand/anion binding mode and multiple diffusion pathways for efficient CO_2/CH_4 and C_2H_2/CH_4 separation. This study is very systematic, comprehensive, and insightful warranting publication in ACS Central Science.

Response: Thanks for the reviewer's positive comment.

Comment 1. It is suggested the authors conduct breakthrough experiments under certain humidities.

Response: Thanks for the reviewer's comment. Accordingly, we conducted dynamic breakthrough experiment on ZUL-100 for CO_2/CH_4 (50/50, v/v) mixture containing 1000 ppm H_2O. The result showed that the separation performance of CO_2/CH_4 on ZUL-100 under humid condition was almost the same as that under the original condition, which was consistent with our previous water stability test by isotherm (Figure S37).

Modification:

(1) Main text, page 5, line 89 in the right column, the sentence "At the same time, the CO_2/CH_4 separation performance was not affected under 1000 ppm H_2O (Figure S37)." was added.

(2) Figure S37 have been added into the Supporting Information:

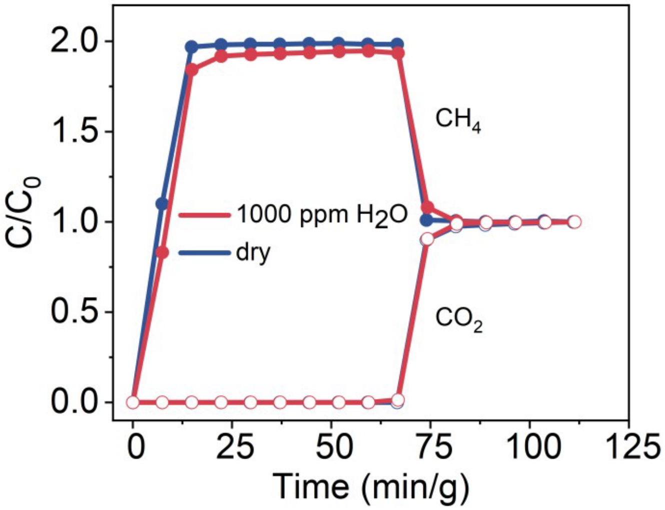

Figure S37. Breakthrough curves for ZUL-100 under flow of CO_2/ CH_4 (50/50) gas mixture in dry and humid (with 1000 ppm H_2O) conditions at 298 K and 1 bar.

Comment 2. It is suggested that the authors measure the kinetic profiles for the adsorption of CO_2, C_2H_2, and CH_4 by ZUL-100.

Response: Thanks for the reviewer's insightful comment. We have added the corresponding tests of the kinetic profiles of CO_2, C_2H_2 on ZUL-100 at 0.5 bar. The results (Figures S15 and S16) showed that ZUL-100 reached adsorption equilibrium in both CO_2 and C_2H_2 within 2 min at 0.5 bar, demonstrating its excellent mass transfer properties. Meanwhile, given that the ZUL-100's very low CH_4 adsorption capacity and affinity (8.1 cm^3/g at 0.5 bar, static uptake; 1×10^{-6}~1.1×10^{-5} cm^3/g at 0.5 bar, IAST uptake in 50/50 CO_2/CH_4 and C_2H_2/CH_4 mixtures), the kinetic factor may have a negligible influence on the separation performance, so we did not include the related tests in the revised version.

Modification:

(1) Main text, page 1, line 18, the phrase "kinetic tests" was added.

(2) Main text, page 2, line 56 in the right column, the phrase "kinetic tests" was added.

(3) Main text, page 5, line 33 in the left column, the sentence "Kinetic adsorption tests of CO_2 and C_2H_2 on ZUL-100 at 298 K were performed. The results (Figures S15 and S16) showed that ZUL-100 reached adsorption equilibrium in both two gases within 2 min at 0.5 bar, demonstrating its excellent mass transfer rate." was added.

(4) Supporting Information, page 3, the sentences "The adsorption kinetics curves at 298 K for ZUL-100 were measured by the Vacuum Vapor/Gas Sorption Analyzer (BDS-VVS, Beishide Instrument Technology (Beijing) Co., Ltd). The material was evacuated by vacuum heating at 100 ℃ overnight before measurement. The assessment criteria for the adsorption equilibrium was set at 0.1 mg/60 min" were added in the section of 'Adsorption kinetics tests'.

(5) Figures S15 and S16 have been added into the Supporting Information:

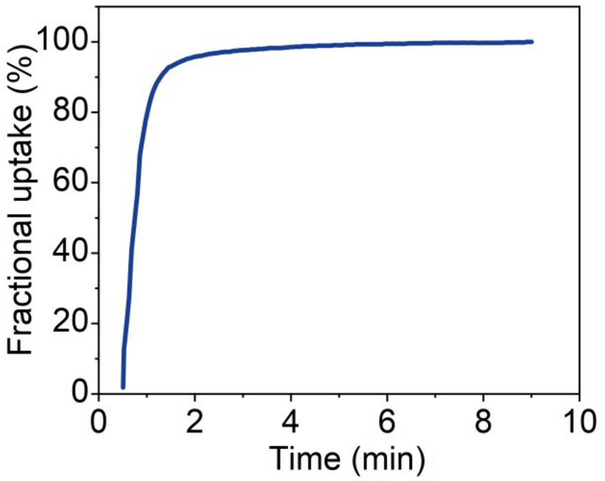

Figure S15. CO_2 kinetic adsorption curves of ZUL-100 at 0.5 bar and 298 K.

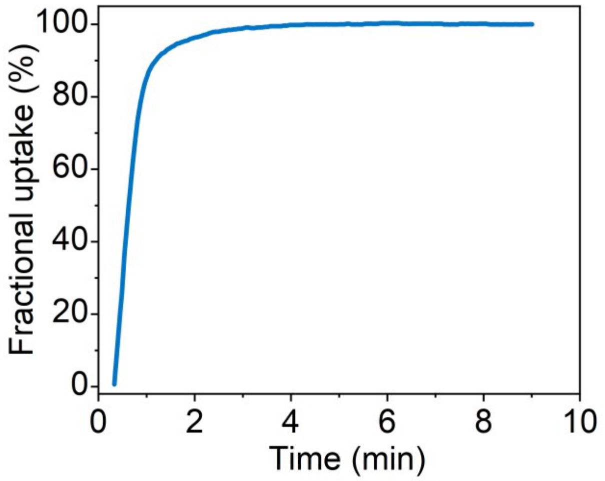

Figure S16. C_2H_2 kinetic adsorption curves of ZUL-100 at 0.5 bar and 298 K.

Comment 3. To monitor the possible structural transformations of ZUL-100 when adsorbing gases, it is suggested that in-situ PXRD patterns of ZUL-100 loaded with CO_2, C_2H_2, CH_4 at different partial pressures be collected.

Response: Thanks for the reviewer's important comment. In-situ PXRD patterns of ZUL-100 loaded with CO_2, CH_4 have been added in the revised version. The results showed that the PXRD patterns were almost the same under different gas loadings, indicating the structural rigidity of the ZUL-100, which well supports our previous structural analysis results. At the same time, we also tried to perform the same test for C_2H_2, but it was difficult to find a qualified test platform due to the dangerous nature of the experiment, as an alternative, we collected the PXRD of ZUL-100 after loading of C_2H_2 at 0.25, 0.5, 0.75 and 1 bar, and the result similarly indicated that the ZUL-100 maintained its structure with the different C_2H_2 loading amount.

Modification:

(1) Main text, page 5, line 37 in the left column, the sentence "In addition, in-situ PXRD patterns of ZUL-100 loaded with CO_2, CH_4 showed that the PXRD patterns were almost the same under different gas loadings, while similar phenomenon was observed in the PXRD pattern of ZUL100 after loading of C_2H_2 at 0.25, 0.5, 0.75 and 1 bar, indicating the structural rigidity of the ZUL-100." was added.

(2) Supporting Information, page 2, section "Powder X-ray diffraction (PXRD)" has been updated: Powder X-ray diffraction and C_2H_2-loaded data of the samples were collected on a SHIMADZU XRD-6000 diffractometer (Cu Kαλ = 1.540598 Å) with an operating power of 40 KV, 30 mA and a scan speed of 4.0°/min. The range of 2θ was from 5° to 60°. CO_2 and CH_4 atmosphere insitu X-ray powder diffraction experiments were conducted on the same instrument using corresponding gases at a rate of 2 mL/min and a scan speed of 30°/min.

(3) Figures S17-19 have been added into the Supporting Information:

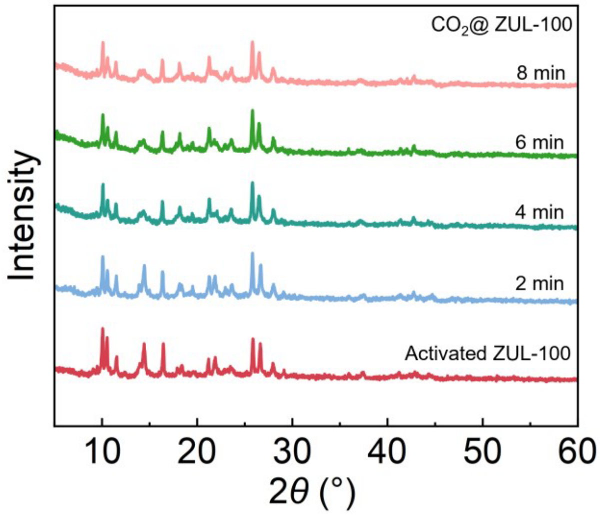

Figure S17. In-situ powder X-ray diffraction patterns of activated ZUL-100, CO_2@ZUL-100 at 1 bar.

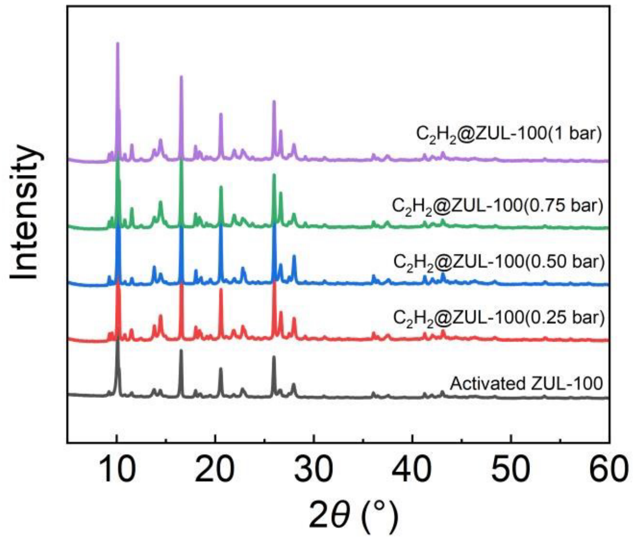

Figure S18 Powder X-ray diffraction patterns of activated ZUL-100, C_2H_2@ZUL-100 at 0.25, 0.5, 0.75 and 1 bar.

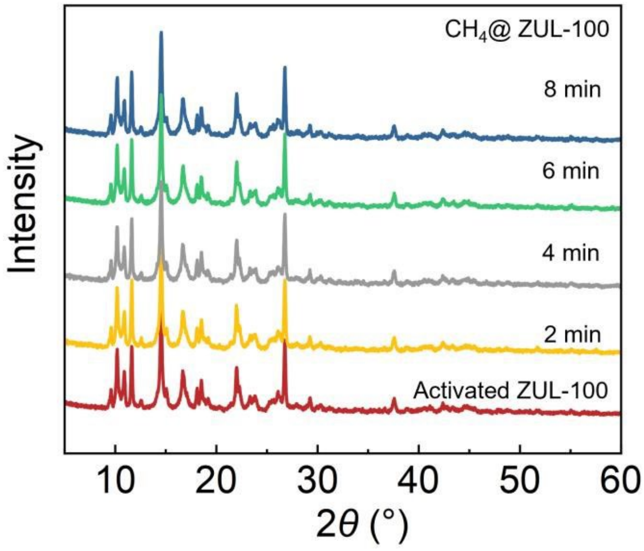

Figure S19 In-situ powder X-ray diffraction patterns of activated ZUL-100, CH_4@ZUL-100 at 1 bar.

We believe that we have satisfactorily addressed all comments and that the manuscript is thereby improved. We thank you in advance for your consideration of this revised manuscript.

It should be noted that, the names of the co-authors have been slightly changed, Zhixin Jiang, Rongrong Fan are added as the fifth and sixth co-author for their contribution to the supplemental experiments in the revised version (Z. J. contributed to the in-situ PXRD tests; R. F. contributed to the synthesis·and·experimental tests). The arrangement is passed by all co-authors including them.

Yours sincerely Best regards,

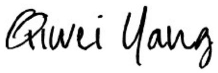

Qiwei Yang, Ph.D.
Professor
College of Chemical and Biological Engineering
Zhejiang University, Hangzhou 310027, China
Email: yangqw@zju.edu.cn
